# Replication origin firing capacity indicates ATR inhibitor sensitivity

**DOI:** 10.1038/s41467-026-74588-7

**Published:** 2026-06-19

**Authors:** A. Lumeau, P. L. Pfuderer, J. A. Scarth, E. Maniati, M. A. Guscott, N. Shaikh, F. B. Copley, H. Gerdes, S. De Angelis, E. L. Alard, J. Wang, P. R. Cutillas, F. K. Mardakheh, M. A. Boemo, J. V. Forment, S. E. McClelland

**Affiliations:** 1 Centre for Cancer Evolution, Barts Cancer Institute, Queen Mary University of London, London, UK; 2 Department of Pathology, University of Cambridge, Cambridge, UK; 3 Cancer Research UK Cambridge Centre, Li Ka Shing Centre, Cambridge, UK; 4 Centre for Haemato-Oncology, Barts Cancer Institute, Queen Mary University of London, London, UK; 5 Centre for Cell and Molecular Biology, Barts Cancer Institute, Queen Mary University of London, London, UK; 6 Department of Biochemistry, University of Oxford, Oxford, UK; 7 Department of Genetics, University of Cambridge, Cambridge, UK; 8 DDR Biology, Bioscience, Oncology R&D, AstraZeneca, Cambridge, UK

**Keywords:** Targeted therapies, Origin firing, Origin firing, DNA damage checkpoints

## Abstract

Inhibitors of ATR, a central kinase controlling DNA replication origin firing and cellular checkpoints, are undergoing clinical trials, yet mechanisms underpinning sensitivity to ATR inhibitors (ATRi) and patient stratification biomarkers are lacking. Here, we perform in parallel, proteomics, transcriptomics and functional analyses and demonstrate that sensitive cancer cell lines have higher expression of DNA replication initiation factors, and exhibit higher origin firing, increased pan-nuclear γH2AX signals and cell death upon ATRi treatment. ATRi sensitivity is causally associated with origin firing rates, since we could modulate ATRi sensitivity by either up- or down-regulating origin firing capacity using CDC7 inhibition, CDK2 inhibition or CDC45 overexpression in both breast and colorectal cancer cells. High expression of replication initiation factors predicts ATRi sensitivity across cell lines from multiple cancer types and acute myeloid leukemia patient samples. This study reveals a contribution of lethal origin firing capacity to ATR sensitivity, providing key steps towards developing a multimodal clinically applicable biomarker.

## Abstract (web summary)

ATR inhibitors target cancer replication stress but lack accompanying clinical biomarkers. Here the authors discover DNA replication origin firing capacity as a predictor of ATR inhibitor sensitivity indicated with protein and RNA readouts.

## Introduction

Replication stress (RS) is defined as the slowing or stalling of DNA replication forks, associated with oncogene activation across several cancer types^1,2^. RS is a known driver of chromosomal instability (CIN), continuous gains and losses of whole or parts of chromosomes during cell division, in multiple cancer types^3–5^. Cell cycle checkpoints, the RS response and DNA repair pathways are crucial for alleviating RS in normal cells, ensuring fidelity during cell division to limit CIN. Thus, an emerging concept in cancer therapeutic strategies is to capitalize on the high levels of RS, genomic instability and/or cell cycle checkpoint defects in cancer cells by inhibiting these protective pathways^6^. Accordingly, several RS response inhibitors are in clinical trials based on the principle that higher underlying rates of RS will sensitize tumours to a further loss of key RS responses or checkpoints^6,7^.

ATR (Ataxia telangiectasia and Rad3 related) is a protein kinase that maintains genomic integrity by regulating origin firing during normal S-phase, and by acting at cell cycle checkpoints to limit entry into mitosis in the presence of RS or DNA damage^8^. ATR inhibition therefore has two major consequences for the cell: unscheduled origin firing and abrogation of the intra-S-phase checkpoint induces widespread RS^9–11^, and cells with damaged genomes can continue into mitosis, as the G2/M checkpoint is weakened, potentially causing unrestrained genomic instability^12^.

ATR kinase inhibitors (ATRi) are an emerging new class of anti-cancer treatment being developed to exploit elevated RS, DNA damage response deficiencies and/or to promote anti-tumour immunity in cancer^12,13^. Multiple ATR inhibitors are in clinical trials as monotherapy or in combination with a diverse range of partner agents and across multiple cancer types (reflecting the complex biology of ATR), although none are yet approved. To date, clinical monotherapy efficacy has been modest and can be associated with dose-limiting toxicities, particularly haematological. Encouraging clinical activities have been recently reported for ATRi in combination with other therapies; for example, Phase 2 clinical studies combining ceralasertib with the poly (ADP-ribose) polymerase (PARP) inhibitor (PARPi) olaparib in PARPi-resistant ovarian cancer patients^14^ or with the anti-PD-L1 immune checkpoint inhibitor durvalumab in immunotherapy-resistant non-small cell lung cancer patients^15^, but no standout patient selection biomarker has been identified^12,16^. It is therefore crucial to improve our understanding of the mechanism of action of ATRi, to identify biomarkers to predict patient response, as well as revealing more effective therapy combinations.

In this study, we aimed to understand the mechanisms contributing to ATRi sensitivity and to explore multimodal biomarkers that could be used in future clinical trials involving ATRi. We confirmed a general link between increased RS and ATRi sensitivity in non-transformed cell lines and demonstrated that sensitive breast cancer cell lines trend towards slower replication fork speeds. All cell lines experienced similar increases in RS and CIN upon ATR inhibition, though sensitive lines exhibited a higher micronucleus rate, suggesting a potential contribution of excessive micronuclei rates to ATRi sensitivity. However, none of these RS nor CIN markers could predict the response of breast cancer cell lines to ATRi, prompting us to explore additional molecular mechanisms responsible for cell death in sensitive cell lines. We performed an unbiased approach, using proteomics and gene expression analyses and discovered that sensitive breast cancer cell lines expressed higher levels of replication initiation factors. Functionally, sensitive cell lines exhibited hyper-increases in origin firing upon ATRi treatment, and ATRi sensitivity was reproducibly reduced by lowering origin firing capacity by inhibiting either CDC7 or CDK2 in two cancer types, breast and colorectal cancer. Increasing origin firing in ATRi-resistant cell lines could also partly sensitise to ATRi. ATRi-sensitive cell lines from multiple cancer types and patient-derived primary cell lines exhibited significantly enriched expression of DNA replication initiation factors, with receiver operating characteristic (ROC) analyses highlighting the potential of this approach to predict ATRi sensitivity in multiple cancer types.

## Results

### ATRi induces RS and CIN in all cell lines, regardless of sensitivity status

**Fig. 1:**
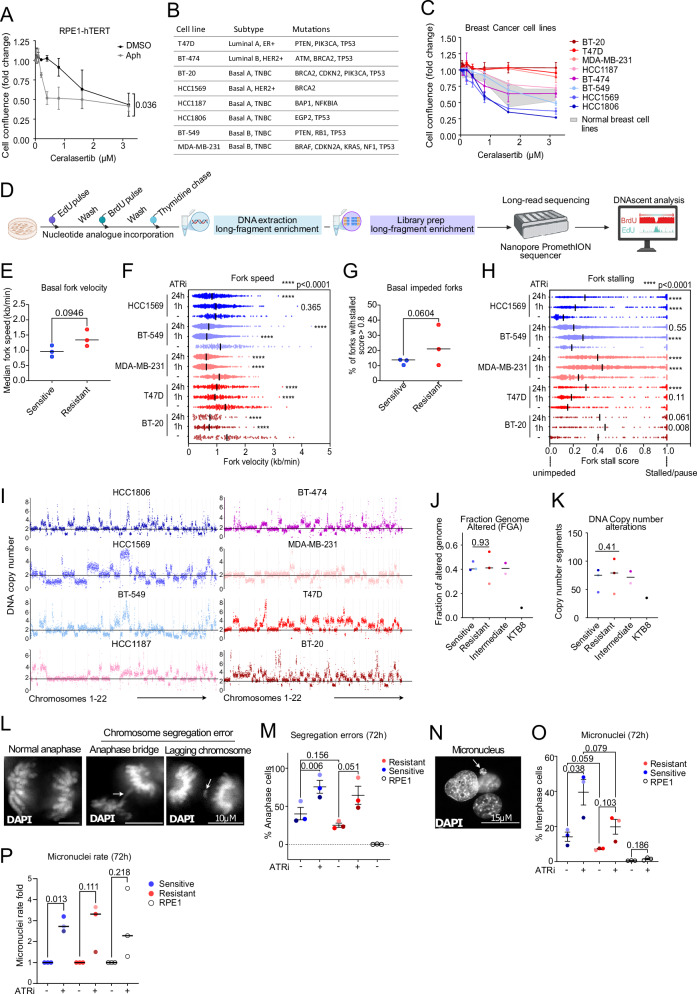
ATRi induces RS and CIN in all cell lines, regardless of sensitivity status. **A** RPE1-hTERT cell confluence after 72 h treatment with ceralasertib (ATRi) and DMSO or Aphidicolin 0.4 µM. Two-way ANOVA analysis. **B** Molecular-genetic features of breast cancer cell lines used herein. **C** Cell confluence after 72 h ceralasertib (ATRi) in breast cancer cell lines. For (**A**, **C**), fold confluence of ATRi-treated samples was measured relative to DMSO at 72 h, data are mean +/− SEM of ≥ three independent biological replicates. **D** Schematic representation of DNAscent. Created in BioRender. Lumeau, A. (2026) https://BioRender.com/yilmlym. **E**, **F** Median fork velocity (kb/min) in sensitive and resistant cell lines at basal (**E**) or after DMSO or 24 h ATRi (**F**). **G**, **H** Proportion of impeded forks (stall score probability above 0.8) in sensitive and resistant cell lines untreated (**G**) or in 24 h DMSO or ATRi (**H**). Statistical tests in (**E**, **G**): two-sided unpaired *t* tests between three sensitive and three resistant cell lines, one biological replicate. Statistical tests in (**F**, **H**): two-sided Mann–Whitney analyses between ATRi and DMSO conditions. Note only the DMSO condition was performed for HCC1806. **I** Genome alteration plots from long-read nanopore sequencing data. **J**, **K** Fraction of genome altered (**J**) and number of copy number segments (**K**) of breast cancer cell lines. Two-sided unpaired *t* test analysis between three sensitive and three resistant cell lines, one biological replicate. **L** Representative images of normal anaphase, anaphase bridge and lagging chromosome. Chromatin visualized with DAPI. **M** Quantification of segregation error rate after DMSO or ATRi 0.5 µM for 72 h. **N** Representative image of interphase cell with a micronucleus. **O**, **P** Micronucleus rate (**O**) and fold change in micronuclei rate (**P**) in cells treated with DMSO or ATRi 0.5 µM for 72 h. Data in (**M**, **O**, **P**) are mean +/− SEM of three sensitive vs three resistant cell lines, in two (HCC1569, BT-549, MDA-MN-231, T47D, BT-20) or three (HCC1806, HCC1569) biological replicates. Statistical tests: two-sided paired *t* test analysis. Source data are provided as a Source data file.

First, to validate the association between RS and ATRi sensitivity, we used two h-TERT immortalized, non-transformed diploid cell lines: RPE1 (retinal pigment epithelial cells) and FNE1 (fallopian tube epithelial cells)^17^. RPE1 cells treated with low doses of the DNA polymerase poison aphidicolin that nonetheless permit cell cycle progression^18^ showed elevated DNA damage in replicating cells (as measured by phosphorylation of histone variant H2AX on Ser-139, also known as γH2AX; Supplementary Fig. 1a), increased micronuclei resulting from CIN (Supplementary Fig. 1b) and were sensitized to ATRi treatment (Supplementary Fig. 1c and Fig. 1A). FNE1 cells treated with aphidicolin, or after induction of *CCNE1* (Cyclin E1) overexpression (Supplementary Fig. 1d), previously implicated as an oncogenic driver of RS^19–21^, were also sensitized to ATRi (Supplementary Fig. 1e), thus confirming a relationship between RS and ATRi sensitivity.

Since ATR inhibitors are currently in clinical trials in many cancer types, including breast cancer, we chose to test the effects of the ATRi ceralasertib (AZD6738) on a panel of breast cancer cell lines. There is no known link between breast cancer subtypes and ATRi sensitivity, so our panel included CCLE (Cancer Cell Line Encyclopedia) cell lines from luminal and basal breast cancer subtypes with different genetic alterations (Fig. 1B). The panel exhibited a range of ATRi sensitivities showed by confluence assays (Fig. 1C and Supplementary Fig. 1c, f), viability assays (Supplementary Fig. 1g) and clonogenic assays (Supplementary Fig. 1h–j), facilitating the discovery of differences between sensitive and resistant lines. As a tissue-specific comparative control, we also assessed the ATRi sensitivity of a panel of three non-transformed breast cell lines (KTB6, KTB8 and KTB34)^22^ (Supplementary Fig. 1k, l). Based on these data, we classified three breast cancer cell lines as more resistant (BT-20, T47D, MDA-MB-231) and three as more sensitive (HCC1569, HCC1806, BT-549) to ATRi than the non-transformed KTB panel, with the remaining two (HCC1187, BT-474) showing an intermediate sensitivity phenotype (Fig. 1C). Previous work has reported that ATRi sensitivity is associated with *CCNE1* (cyclin E) amplification in different cellular models^21,23^. However, using publicly available transcriptomic data and sensitivity to ceralasertib of breast cancer cell lines, we found no correlation between ATRi sensitivity and expression of *CCNE1*, *CCNE2* or *MYC* (all potential oncogenic drivers of RS) (Supplementary Fig. 1m).

RS is a driver of CIN; incomplete DNA replication, mitotic DNA breakage of fragile sites, and a multitude of other mechanisms can all drive the generation of chromosome segregation errors and micronuclei^4,18,24,25^. Therefore, either RS or the resulting increase in CIN may be responsible for ATRi sensitivity. We hypothesized that the cell lines most sensitive to ATRi would demonstrate the highest levels of RS (and thus the slowest replication fork rates^1,26^). To directly assess RS levels, we examined DNA replication dynamics using a long-read sequencing method, DNAscent, which detects replication fork speed and stall frequency using machine learning to call base analogues in recently replicated DNA^27^ (Fig. 1D). In baseline conditions (without ATRi treatment), we observed a lower fork speed in sensitive cell lines (especially HCC1806 and HCC1569) and a higher rate of impeded forks in resistant cell lines (especially MDA-MB-231 and BT-20), though not reaching significance (Fig. 1E, G), and no differences in S-phase γH2AX foci, a global mark of replication-associated DNA damage (Supplementary Fig. 1n). Since ATRi impacts both origin firing rates and RS response signalling, we performed DNAscent after 1 or 24 h ATRi treatment to capture both the initial and longer-term impact of ATR inhibition on DNA replication kinetics. In response to ATRi, all cell lines showed a significant decrease in fork speed, an increase in fork stalling, or both, demonstrating increased RS regardless of sensitivity status (Fig. 1F, H). Overall, therefore, canonical biological markers of RS could not robustly distinguish ATRi-sensitive cell lines.

We next determined whether rates of RS-induced CIN were different between sensitive and resistant lines. We analysed DNA copy number alterations (CNAs) derived from Oxford Nanopore long-read whole genome sequencing and observed that all lines exhibited chaotic genomes with gains and losses of whole and partial chromosomes (Fig. 1I). Neither the fraction of genome altered nor the number of CNAs differed between sensitive and resistant lines (Fig. 1J, K). We then measured ongoing CIN by scoring the rates of chromosome mis-segregation and micronuclei before and after treatment with ATRi. Baseline rates of CIN were not significantly different between the sensitive and resistant lines (Fig. 1L–O and Supplementary Fig. 1o), and ATRi treatment caused similar relative increases in segregation errors and micronuclei across all cell lines (Fig. 1P). The universal increase in CIN following ATRi treatment also excludes differences in drug uptake or efflux as a cause of differential ATRi sensitivity. We noted that *absolute* micronuclei rates were significantly higher after ATRi in sensitive cells (Fig. 1L and Supplementary Fig. 1o–q), potentially contributing to ATRi sensitivity in sensitive breast cancer cell lines. However, overall, baseline rates of CIN or RS could not readily predict the differential ATRi sensitivity of this panel of breast cancer cell lines.

We and others have previously shown that supplementing cells with exogenous nucleosides can reduce RS and CIN^24,25,28^. In line with the absence of strong associations between RS or CIN levels and ATRi sensitivity in these cell lines, supplementing cells with nucleosides only partly rescued the sensitivity in HCC1806 (Supplementary Fig. 1r), suggesting other underlying mechanisms of sensitivity to ATRi.

### Increased origin firing capacity renders sensitive cell lines more vulnerable to cell death following ATR inhibition

**Fig. 2:**
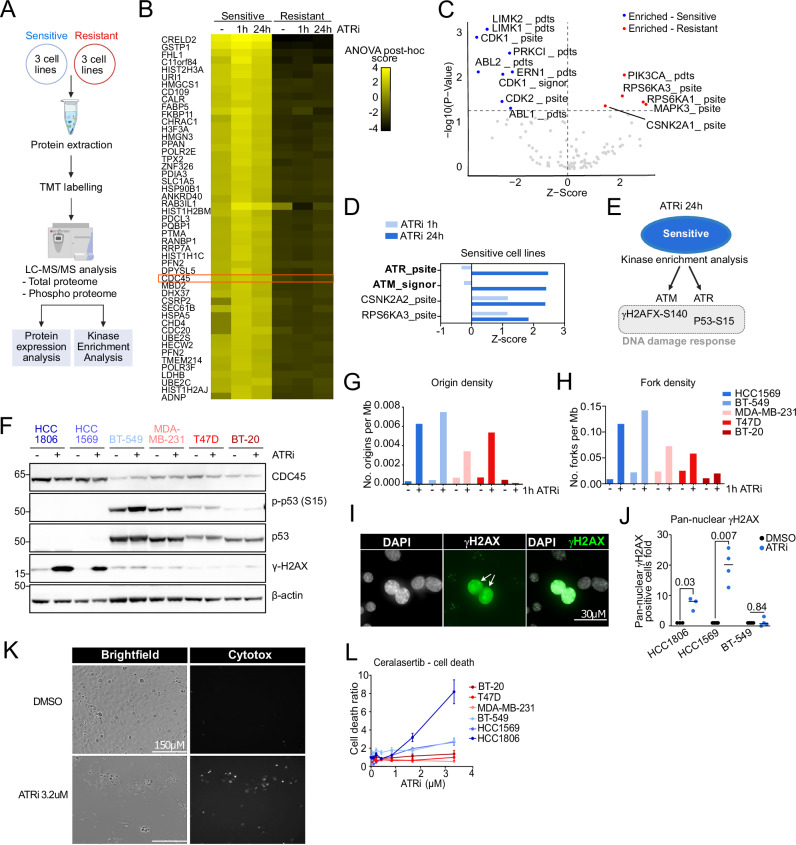
Increased origin firing capacity renders cancer cell lines more vulnerable to ATR inhibition. **A** Schematic representation of mass spectrometry method to obtain total proteome and phosphoproteomes of breast cancer cell lines. Created in BioRender. Lumeau, A. (2026) https://BioRender.com/a0768dq. **B** Heatmap showing the top hit significantly enriched proteins in sensitive compared to resistant cell lines. Orange box indicates CDC45 protein. Significantly altered proteins were identified using a one-way ANOVA, with post hoc score calculation (FDR < 0.05). **C** Volcano plot of kinase activities most enriched in sensitive and resistant breast cancer cell lines, obtained by kinase enrichment analysis of phosphoproteomics data. Two-sided Kolmogorov–Smirnov. **D** Quantification of kinase activities enriched after 24 h of 0.5 µM ATRi in sensitive cell lines compared to resistant, *Z*-scores higher than 1.5. **E** Schematic representation of the enrichment of kinases ATM and ATR via activation of substrates γH2AX-Ser140 and p53-Ser15. Created in BioRender. Lumeau, A. (2026) https://BioRender.com/skz4ixc. **F** Western blotting for CDC45, p53-Ser15, p53 and H2AX-Ser139 after 24 h DMSO or 0.5 µM ATRi in breast cancer cell lines. Beta-actin was used as a loading control. **G**, **H** Quantification of replication origins (**G**) and fork density per megabase (**H**) in breast cancer cell lines treated with DMSO or 0.5 µM ATRi for 1 h. Data obtained from R9 long-read DNA sequencing in one biological replicate (see “Methods”). **I** Example of pan-nuclear γH2AX staining in the cell population. **J** Percentage of cells with pan-nuclear γH2AX staining in three sensitive cell lines treated with DMSO or 0.5 µM ATRi for 24 h. Data are mean +/− SEM of three (HCC1806, BT-549) or four (HCC1569) independent replicates, two-sided paired *t* test analysis. **K** Example of brightfield and green cytotox fluorescence images of DMSO and 3.2 µM ATRi-treated HCC1806. **L** Cell death ratio obtained by measuring green cytotox fluorescence in breast cancer cell lines treated with DMSO or 72 h ATRi (0.4, 0.8, 1.6, 3.2 µM). Data are mean +/− SEM of two (HCC1569, BT-549, BT-20) or three (HCC1806, T47D, MDA-MB-231) independent replicates. Source data are provided as a Source data file.

To search in an unbiased manner for the specific mechanisms causing differential ATRi sensitivity, we performed total and phosphoproteomic analyses using tandem mass tagging (TMT) mass spectrometry (MS) to allow quantitative comparisons between sensitive and resistant cell lines (Fig. 2A). For this, we treated the three most resistant or sensitive breast cancer cell lines with dimethyl sulfoxide (DMSO) or ATRi for 1 or 24 h before TMT, detecting 5773 phosphosites across 5000 proteins. Post-hoc scores were measured using an analysis of variance (ANOVA) across all conditions. Under baseline (untreated) conditions, CDC45, a key regulator of replication origin firing^11,29^, was among the top 50 enriched proteins in sensitive compared to resistant lines (Fig. 2B). We therefore searched for additional proteins in the origin firing pathway using the 37 gene “DNA replication initiation” gene ontology (GO) term. Of the 20 members detected in our dataset, three additional origin firing proteins (ORC1, KAT7, WRNIP1) were found to be enriched in sensitive versus resistant cell lines (Supplementary Fig. 2a). We also confirmed the increase of *CDC45* expression in sensitive cell lines at the mRNA level using publicly available data (Supplementary Fig. 2b) and at the protein level by western blot in HCC1806 and HCC1569 (Supplementary Fig. 2c, d). Turning next to the phosphoproteome, we searched for differences in the cellular signalling consequences of ATRi treatment between the sensitive and resistant lines. Unfortunately, we could not directly identify several key phosphorylation sites of interest, including the CHK1-Ser345 phosphorylation site, a direct substrate of ATR^30^. We therefore used a Kinase Enrichment Analysis Method (KSEA), which allows the inference of activated kinases from the presence of downstream target phosphorylation events^31^. Using this analysis, we noted that CDK2 and CDC7 signalling pathways, which are linked to DNA replication, were enriched under baseline conditions in sensitive cell lines compared to resistant cell lines (Fig. 2C and Supplementary Fig. 2e). In addition, only sensitive cell lines exhibited evidence of increased DNA damage signalling upon 24 h of ATRi treatment, with increased ATR and ATM pathway activation, exemplified by the specific phosphorylation of H2AX and p53 (Fig. 2D, E and Supplementary Fig. 2f). Note that using this analysis approach, ATR signaling pathways are detected as active despite the presence of ATRi due to the overlap in substrates between the ATR and ATM signaling pathways^32^. To deconvolute the contribution of these alterations from individual cell lines, we performed western blot experiments in cells treated with ATRi for 24 h and showed that the sensitive cell lines with high CDC45 expression (HCC1806, HCC1569) have a strong activation of γH2AX, but do not express p53 (Fig. 2F and Supplementary Fig. 2g, h). In contrast, BT-549 exhibited a strong increase in p53-Ser15 phosphorylation upon ATRi, but no change in γH2AX. These results are in line with the MS data showing activation of γH2AX and p53-Ser15 in response to ATRi in sensitive cells as a collective, but additionally reveal the cell line-specific contributions to these signals.

To determine whether the high origin firing factor expression levels in sensitive cell lines led to an elevated increase in replication origin firing upon ATRi, we analysed the frequency of DNA replication origins per megabase of DNA (origin density) in our DNAscent data. No difference in origin density was detected between sensitive and resistant cells in untreated conditions, though it is likely that the limit of detection for this assay precludes this observation (Fig. 2G). As expected, ATRi treatment for 1 h resulted in increased origin firing in most of the cell lines, in line with the known role for ATR in suppressing origin firing^9^. However, the increase in origin and fork densities was overall higher in the sensitive cell lines tested (BT-549, HCC1569) compared to resistant cell lines (MDA-MB-231, T47D, BT-20) (Fig. 2G, H and Supplementary Fig. 2i).

We reasoned that hyper-increased origin firing could render cells more sensitive to ATRi through exhaustion of DNA replication factors, leading to replication failure and cell death^33,34^. Accordingly, total γH2AX intensity per cell in S-phase was higher in two sensitive cell lines (HCC1806, HCC1569), compared to resistant ones (Supplementary Fig. 2j), and increased proportions of interphase cells exhibited pan-nuclear γH2AX staining, an indicator of replication catastrophe^34,35^ (Fig. 2I, J and Supplementary Fig. 2k). This could contribute to the observed increased cell death upon ATRi treatment (Fig. 2K, L). Increased proportions of pan-nuclear γH2AX-positive cells upon ATRi were not observed in resistant cell lines or in non-transformed cell lines (Supplementary Fig. 2k, l). Overall, these data suggest that an increased capacity for origin firing in sensitive cell lines promotes lethal levels of origin firing, catastrophic RS and subsequent cell death upon ATRi treatment.

### Modulating origin firing influences sensitivity to ATR inhibition

**Fig. 3:**
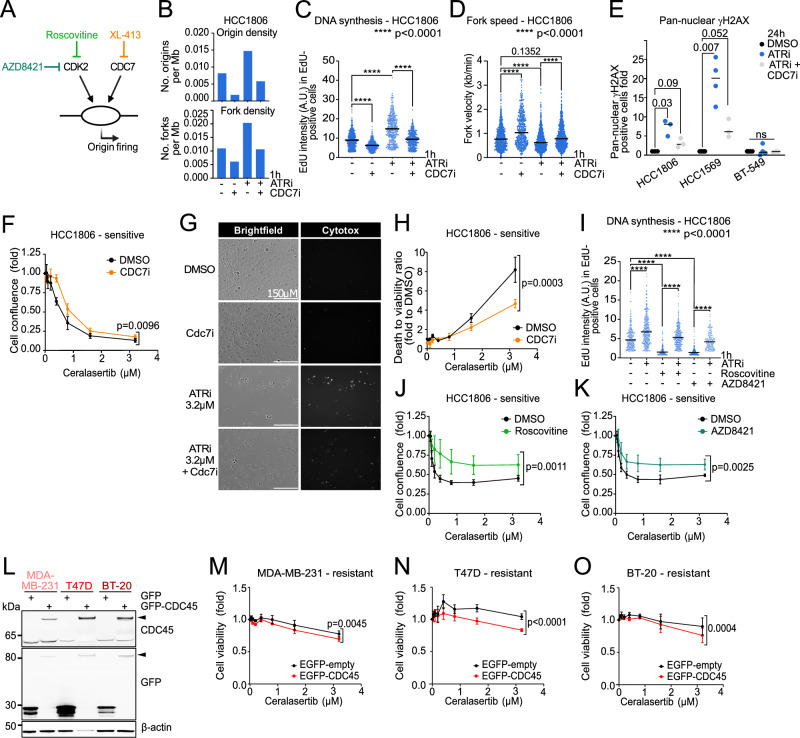
Modulating origin firing influences sensitivity to ATR inhibition. **A** Schematic of strategies to inhibit origin firing. Created in BioRender. Lumeau, A. (2026) https://BioRender.com/ajmdcrv. **B** Replication origin and fork density/Mb, with DMSO, 0.5 µM ATRi and 1 µM CDC7i as indicated for 1 h, from R10.4.1 long-read DNA sequencing in one biological replicate (“Methods”). **C** Mean EdU intensity in replicating (EdU-positive) cells treated with DMSO, 0.5 µM ATRi and 1 µM CDC7i as indicated for 1 h, mean +/− SD of 250–500 EdU-positive cells per condition. Two-sided Mann–Whitney test. **D** Fork velocity (kb/min) in cells treated with DMSO, 0.5 µM ATRi and 1 µM CDC7i as indicated for 1 h, from R10.4.1 long-read DNA sequencing (“Methods”), mean +/− SD with two-sided Mann–Whitney analysis. **E** Pan-nuclear γH2AX staining after 24 h DMSO, 0.5 µM ATRi, 1 µM CDC7i as indicated, mean +/− SEM of three (HCC1806, BT-549) or four (HCC1569) independent biological replicates, two-sided paired *t* test analysis. **F** Fold-change in cell confluence in ATRi, DMSO or 1 µM CDC7i as indicated, two-way ANOVA analysis. **G** Representative image from Cytotox analysis in DMSO, ATRi 3.2 µM and CDC7i 1 µM as indicated. **H** Fold-change in cell death after DMSO or 1 µM CDC7i, mean +/− SEM of three independent biological replicates, two-way ANOVA analysis. **I** EdU intensity in replicating (EdU-positive) cells treated with DMSO, 10 µM roscovitine, 1 µM AZD8421 and 0.5 µM ATRi as indicated for 1 h, two independent replicates, 250–500 EdU-positive cells per condition, two-sided Mann–Whitney test analysis. **J**, **K** Cell confluence fold-change in ATRi combined with DMSO and 10 µM roscovitine (**J**) or 1 µM AZD8421 (**K**), mean +/– SEM of three independent biological replicates, two-way ANOVA analysis. **L** Western blotting for GFP and CDC45 48 h post-transfection with empty-EGFP or EGFP-CDC45 plasmids. Beta-actin is a loading control. Blot representative of three independent experiments. **M**–**O** Cell viability fold-change of resistant MDA-MB-231 (**M**), T47D (**N**) and BT-20 (**O**) in ATRi, combined with DMSO or 1 µM CDC7i, mean +/− SEM of three independent biological replicates, two-way ANOVA analysis. Source data are provided as a Source data file.

To test whether hyper-increased ATRi-induced origin firing was responsible for ATRi sensitivity, we first sought to manipulate origin firing rates (Fig. 3A). We used XL-413, an inhibitor of CDC7, a positive regulator of origin firing^11^, henceforth termed ‘CDC7i’. In the absence of ATRi, CDC7i reduced origin firing, as measured by DNAscent origin density and imaging EdU incorporation, and increased fork speeds in sensitive HCC1806 cells, as expected (Fig. 3B–D)^36^. Importantly, CDC7i rescued the ATRi-dependent effects on origin firing and replication fork speed in HCC1806 cells (Fig. 3B–D).

To determine whether modulating origin firing could influence the sensitivity of breast cancer cell lines to ATRi, we analysed pan-nuclear γH2AX quantification and cell viability in response to ATRi treatments with and without CDC7i. Remarkably, CDC7i largely rescued the levels of ATRi-induced pan-nuclear γH2AX in HCC1806 and HCC1569 (Fig. 3E and Supplementary Fig. 3a). This suggests that ATRi-induced pan-nuclear γH2AX is indeed due to excessive origin firing likely leading to replication failure and cell death.

Importantly, CDC7i reduced the anti-proliferative effect of ATRi in two sensitive cell lines (HCC1806 and HCC1569) (Fig. 3F and Supplementary Fig. 3b), whereas in the two resistant cell lines tested for cell confluence assays there was either no effect, or in fact a sensitization to ATRi (Supplementary Fig. 3c). Further cell viability and cytotoxic assays confirmed these results (Supplementary Fig. 3d, e and Fig. 3G, H).

We performed similar assays to inhibit origin firing via inhibition of CDK2, another key activator of the pathway, using two independent inhibitors: roscovitine, well described as a cell cycle and origin firing inhibitor^37–39^, and the more recently developed and more potent molecule AZD8421^40^. Both CDK2 inhibitors induced a drastic and expected reduction of origin firing in HCC1806 breast cells, exemplified by the decrease of EdU incorporation (Fig. 3I) and partially suppressed the ATRi-dependent increase of origin firing (Fig. 3I). Importantly, CDK2 inhibition efficiently desensitized cell lines to ATRi in terms of both cell proliferation (Fig. 3J, K) and cell viability (Supplementary Fig. 3f, g), without impacting cell proliferation or cell viability when applied alone (Supplementary Fig. 3h). Altogether, these results indicate that reducing origin firing by either CDC7 or CDK2 inhibition limits the anti-proliferative and cytotoxic effects of ATRi specifically in sensitive cell lines.

We next reasoned that, if resistant cell lines are able to escape ATRi-mediated cell death by limiting excessive origin firing, then inducing *higher* origin firing rates in ATRi-resistant cells might reverse ATRi resistance. To investigate this, and given that CDC45 is already highly expressed in sensitive cell lines (Fig. 2B and Supplementary Fig. 2a–d), we transiently overexpressed CDC45 (as done previously to increase origin firing^11^) in the three resistant breast cancer cell lines MDA-MB-231, T47D and BT-20. Western blot assays confirmed the increase of expression of CDC45 protein levels in all three cell lines (Fig. 3L). Interestingly, CDC45 overexpression led to a mild but significant sensitization to ATRi in all three resistant cell lines (Fig. 3M–O), while having no impact on cell viability in the absence of ATRi (Supplementary Fig. 3i). This demonstrates that origin firing must be tightly regulated in cancer cell lines to resist ATRi, since decreasing origin firing (with CDC7i or CDK2i) or increased origin firing (with overexpression of CDC45) could both reduce the viability of cell lines initially resistant to ATRi, particularly exemplified by T47D which was sensitive to both manipulations (Supplementary Fig. 3c, d and Fig. 3O). Altogether, our results provide evidence that origin firing capacity is an important factor in determining sensitivity to ATRi and that manipulating origin firing can modulate the ATRi response in both directions.

### A transcriptomic signature of DNA replication initiation correlates with sensitivity to ATRi across cell lines from different cancer types

**Fig. 4:**
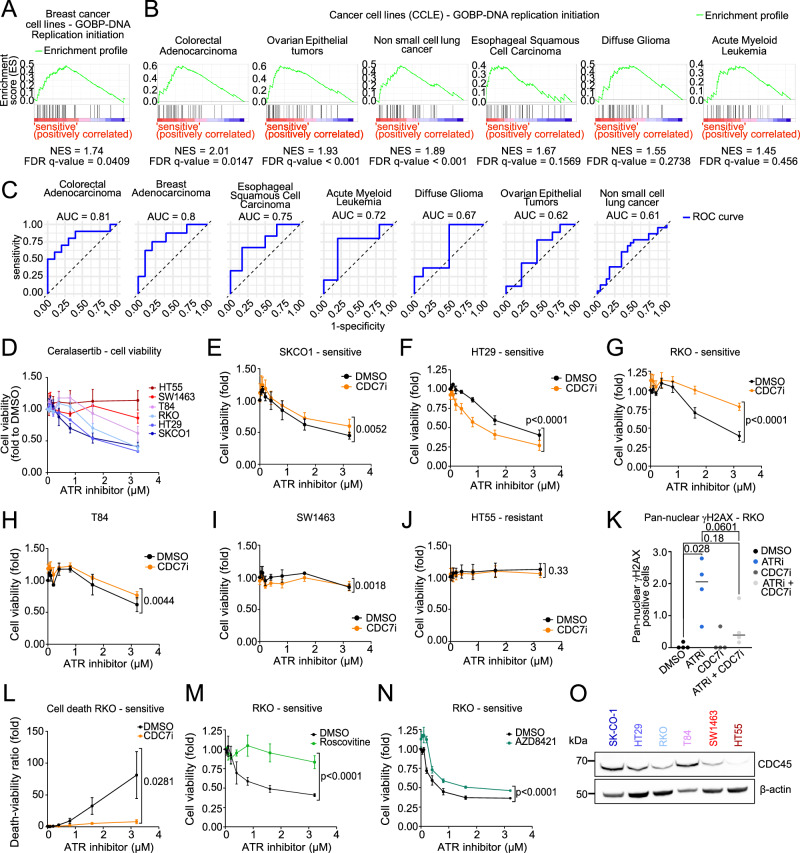
Transcriptomic signature of DNA replication initiation correlates with sensitivity to ATRi across cell lines from different cancer types. **A**, **B** Gene Set Enrichment Analysis (GSEA) of publicly available transcriptomic data showing enrichment of DNA replication initiation signature (GO-terms biological processes) in sensitive compared to resistant breast cancer cell lines (**A**) and different cancer type cell lines from CCLE (**B**). Sensitivity and resistance were determined using the top and bottom 25% of ceralasertib IC50 from the GDSC2 dataset. Normalized enrichment scores (NES) and FDR *q*-values obtained through the GSEA analysis, nominal *p* values derived empirically from permutation testing, and multiple-testing correction was performed using the FDR method implemented in GSEA. **C** ROC curve analysis of CCLE datasets demonstrates performance of the DNA replication initiation pathway in predicting ATRi sensitivity. Receiver operating characteristic (ROC) curves and area under the curve (AUC) were generated following linear discriminant analysis (LDA) with leave-one-out cross-validation. Cell lines used: *n* = 10 per group (colorectal cancer) *n* = 10 in sensitive, *n* = 8 in resistant group (breast cancer); *n* = 6 per group (oesophageal squamous cancer), *n* = 5 per group (AML cell lines), *n* = 8 per group (diffuse glioma), *n* = 9 per group (ovarian cancer), *n* = 23 per group (non-small cell lung cancer). **D**–**J** Cell viability fold-change after 72 h ATRi in six colorectal cancer cell lines (**D**) and in DMSO and CDC7i in SKCO1 (**E**), HT29 (**F**), RKO (**G**), T84 (**H**), SW1463 (**I**), HT55 (**J**), mean +/− SEM of three independent biological replicates, two-way ANOVA analysis. **K** Frequency of pan-nuclear γH2AX staining in RKO cells treated with DMSO, 0.5 µM ATRi, 1 µM CDC7i as indicated for 24 h, mean +/− SEM of four independent replicates. Two-sided paired *t* test analysis. **L** Cell death ratio in RKO cells in ATRi, DMSO or 1 µM CDC7i as indicated. **M**, **N** Fold-change in cell confluence in ATRi, and DMSO or 1 µM roscovitine (**M**) or 1 µM AZD8421 (**N**), mean +/− SEM of three independent biological replicates. Two-way ANOVA analysis. **O** Western blotting for CDC45 in six colorectal cancer cell lines. Beta-actin is loading control. Blot representative of three independent experiments. Source data are provided as a Source data file.

We next explored whether we could predict ATRi sensitivity based on increased origin firing capacity markers in a larger panel of breast cancer cell lines, taking advantage of publicly available breast cancer CCLE transcriptomic data and previously measured IC50 values of ceralasertib from the genomics of drug sensitivity in cancer dataset (GDSC2)^41^. We identified the groups of most sensitive (lower quartile) and most resistant cell lines (upper quartile) based on division of the dataset into IC50 quartiles (Supplementary Fig. 4a). We then performed a Gene Set Enrichment Analysis (GSEA) to compare ceralasertib-sensitive and resistant breast cancer cell lines. Among the 200 most enriched pathways from gene ontology biological process (GO-BP) terms, we counted the number of pathways related to each category and measured the average normalized enrichment score (NES). In sensitive cell lines, we found enrichment of mitosis-related, and DNA replication pathways (Supplementary Fig. 4b). Given our findings regarding origin firing capacity above, we also analysed the enrichment of the ‘DNA replication initiation’ signature which was highly enriched in breast cancer (NES = 1.74, adjusted false discovery rate (FDR) *q*-value = 0.0409) (Fig. 4A). This suggests that there is a gene expression footprint of higher origin firing capacity in sensitive cell lines.

Clinical trials involving ATRi are being carried out in a wide range of cancer types^12^. We therefore explored whether gene expression signatures could also be indicative of ATRi sensitivity in additional cancer types. We performed a GSEA of sensitive and resistant cancer cell lines from CCLE using publicly available RNA-sequencing data and IC50 from GDSC2, as we did for breast cancer cell lines. In this pan-cancer analysis, the highest NES across multiple cancers was for the DNA replication-related pathways (Supplementary Fig. 4c). Moreover, we found that the more specific “DNA replication initiation” signature was significantly enriched in ceralasertib-sensitive cell lines from multiple cancer types with a particularly high enrichment in colorectal adenocarcinoma and ovarian epithelial tumours (Fig. 4B).

To further evaluate whether the DNA replication initiation signature could be indicative of sensitivity to ATRi, we performed linear discriminatory analysis (LDA) with leave-one-out cross-validation (LOOCV) and generated ROC curves assessing sensitivity and specificity of predicting response for each dataset^42^. Colorectal adenocarcinoma, breast adenocarcinoma, oesophageal squamous cell carcinoma and acute myeloid leukaemia (AML) cell lines showed a fair (>0.7) to good (>0.8) area under the curve (AUC). The DNA replication initiation signature showed poorer classification performance for diffuse glioma, ovarian epithelial tumour and non-small cell lung cancer cell lines (Fig. 4C). These results suggest that the potential for the DNA replication initiation signature to predict the sensitivity to ATRi is higher in colorectal, breast, oesophageal squamous cancers and AML.

Focusing on colorectal adenocarcinoma, which had the highest NES and AUC scores in our above analyses, we next performed a series of assays to investigate whether high origin firing was also a functional modulator of ATRi sensitivity in this cancer type. We first evaluated the sensitivity of a panel of six colorectal cell lines to ATRi through viability assays, identifying one resistant cell line (HT55), three sensitive cell lines (SKCO1, HT29 and RKO) and two intermediate cell lines (T84, SW1463) (Fig. 4D). We then assessed whether limiting origin firing using CDC7i and CDK2i as above for the breast cancer lines could rescue sensitivity of these six cell lines in response to ATRi. CDC7i efficiently rescued the effects of ATRi on cell viability in four out of the five sensitive/intermediate cell lines (SKCO1, RKO, T84 and SW1463, Fig. 4E–I), and had no impact on HT55 cells, the most resistant to ATRi (Fig. 4J). Moreover, the proportion of pan-nuclear γH2AX and cell death also decreased following CDC7i treatment in sensitive cell line RKO (Fig. 4K, L). Finally, we observed that CDK2i treatment could also rescue ATRi-dependent increased origin firing and decreased cell proliferation and viability in sensitive RKO cells (Fig. 4M, N and Supplementary Fig. 4d), again not impacting cell proliferation or viability alone (Supplementary Fig. 4e).

As we observed high CDC45 protein expression in the breast cancer cell lines sensitive to ATRi, we also analysed protein levels of CDC45 in the colorectal cell line panel. This revealed that although CDC45 protein levels were somewhat variable between colorectal cell lines, the most resistant cell line HT55 displayed markedly reduced CDC45 expression (Fig. 4O). We also observed an increase of γH2AX in response to ATRi in all colorectal cell lines except for the resistant HT55 (Supplementary Fig. 4f). Altogether, these data support that the correlation between levels of origin firing factors and sensitivity to ATRi we discovered in breast cancer can be extended to a second major cancer type: colorectal cancer.

### High origin firing capacity as a predictive marker of response to ATR inhibition in pancreatic ductal adenocarcinoma (PDAC) patient-derived primary cells and AML patient samples

**Fig. 5:**
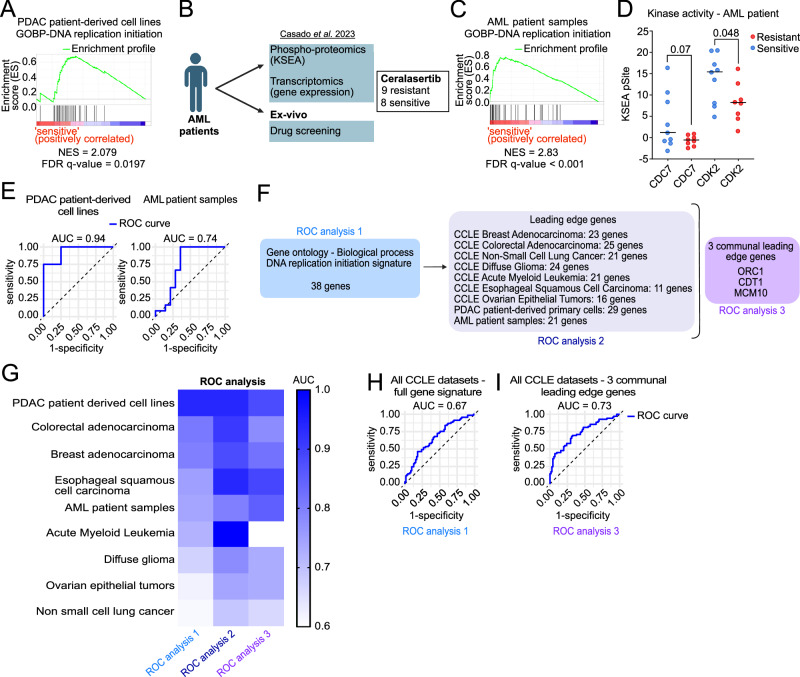
Exacerbated origin firing as a predictive marker of response to ATR inhibitor in cancer samples. **A** Gene Set Enrichment Analysis of the GO-BP DNA replication initiation signature in ATRi-sensitive PDAC patient-derived primary cell lines from ref. ^43^. Sensitivity and resistance were determined using the top and bottom 25% of ceralasertib IC50, normalized enrichment scores (NES) and FDR *q*-values were measured through the GSEA analysis. **B** Schematic workflow of AML patient cells data used from Casado et al. for analysis^59^. Sensitivity to ceralasertib obtained via ex vivo drug screening. Created in BioRender. Lumeau, A. (2026) https://BioRender.com/aw9hdx1. **C** GSEA analysis of DNA replication initiation GO signature in AML patient responders versus non-responders to ceralasertib (NES = normalized enrichment score, FDR *q*-value). Nominal *p* values derived empirically from permutation testing, multiple-testing correction performed using FDR method implemented in GSEA. **D** Kinase Enrichment Analysis (KSEA) in AML patients showing enrichment of CDC7 and CDK2 activities in nine good responders versus eight non-responders to ATRi. Two-sided unpaired *t* test analysis. **E** ROC curve analysis demonstrates performance of DNA replication initiation pathway in predicting ATRi sensitivity on patient-derived datasets. ROC curves and AUC were generated following LDA with leave-one-out cross-validation. For PDAC patient-derived primary cell lines, *n* = 4 per group and for AML patient samples, *n* = 12 in the sensitive, and *n* = 14 in the resistant group. **F** Schematic representation of the three different ROC analyses performed using (i) the whole DNA replication initiation signature of 38 genes, (ii) leading-edge genes of the signature per dataset, identified through GSEA analysis (Supplementary Data 21–24) and (iii) the restricted signature of three communal leading-edge genes in all datasets (ORC1, CDT1, MCM10). Created in BioRender. Lumeau, A. (2026) https://BioRender.com/iksecug. **G** Heatmap of AUC values (white poor and blue excellent) measured via ROC analysis in all datasets using three different gene expression signatures, as explained in (**F**). **H**, **I** ROC curve analysis of the full 38 gene signature (**H**) and the three communal leading-edge genes of DNA replication initiation (**I**) in all seven CCLE datasets combined, *n* = 71 in the sensitive, and *n* = 69 in the resistant group. Source data are provided as a Source data file.

Finally, we investigated whether our newly identified transcriptomic and proteomic determinants of ATRi sensitivity could be validated in cancer patient samples. We identified a study of PDAC patient-derived cell lines with transcriptomic and ceralasertib IC50 data^43^. We performed GSEA on the sensitive and resistant IC50 quartiles as before. This revealed a highly significant enrichment of the DNA replication initiation signature (NES = 2.079, adjusted FDR *q*-value < 0.0197) in the sensitive patient-derived primary cells (Fig. 5A). Moving to patient samples, we analysed data from a recent study that performed transcriptomic and proteomic analyses from AML patient samples, also deriving primary cell lines which were subject to a drug screen that included ceralasertib^31^. Using this data, we identified nine resistant and eight sensitive primary cell lines based on IC50 quartiles as before and investigated the related patient data for gene expression and kinase activities (Fig. 5B). We first performed unbiased GSEA in AML patients and found that even though mitosis-related pathways were most represented (more than 40 pathways among 200), the DNA replication-related pathways were the most enriched in sensitive samples (average NES = 2.2 vs NES = 2 for mitosis-related pathways; Supplementary Fig. 5a). Additionally, we found that the “DNA replication initiation” signature was even further enriched (adjusted FDR *q*-value < 0.001, NES = 2.83) (Fig. 5C). We next analysed the phosphoproteomics data from the AML patient samples and discovered an enrichment of the kinase activities of CDC7 and CDK2, notably involved in origin firing^44^, in ATRi-sensitive samples (Fig. 5D). Altogether, our AML patient data analyses recapitulate the ATRi sensitivity predictors discovered in our cancer cell lines: the elevated gene expression of the “DNA replication initiation” signature, and increased activity of kinases involved in origin firing.

We then performed LDA as described earlier and generated ROC curves of both PDAC patient-derived primary cell lines and AML patient samples to evaluate whether the DNA replication initiation signature could be indicative of sensitivity to ATRi, as done above for CCLE cell lines. We found a fair AUC value for the AML patient samples (>0.7) and an excellent value for the PDAC patient-derived primary cell lines (<0.9) (Fig. 5E), confirming the potential of the DNA replication initiation signature to predict sensitivity to ATRi in patient samples from these cancer types.

As the specificity and sensitivity of the signature to predict ATRi response is variable among different datasets and cancer types, e.g. excellent in PDAC patient-derived cell lines and poor in non-small cell lung cancer, we investigated whether a subset of genes within the DNA replication initiation signature would be sufficient or more efficient at predicting ATRi sensitivity in a pan-cancer manner. For that, we performed and compared the following ROC analyses: analysis 1 using the full GO-term biological process (BP) DNA replication initiation signature of 38 genes; analysis 2 using the leading-edge genes in each dataset; analysis 3 using the leading-edge genes common between all the datasets (Fig. 5F). The leading-edge genes per dataset were extracted from the GSEA analysis and corresponded to the genes that contributed to the enrichment of the DNA replication initiation pathway in the sensitive samples versus resistant (Supplementary Data 22–25). We observed between 11 and 29 leading-edge genes per dataset. When examining the overlap of these, we found three communal genes consistently in the leading edge across the datasets (hypergeometric test, *p* value = 0.0005): *ORC1*, *CDT1* and *MCM10* (Fig. 5F). The three ROC analyses allowed us to compare the efficiency of each signature to predict sensitivity to ATRi (Fig. 5F, G). As expected, we found that ROC analysis 2 using the cancer type-specific leading-edge genes showed higher and better predictive AUC scores across all datasets (Fig. 5G and Supplementary Fig. 5b). For non-small cell lung cancer cell lines, none of the signatures provided good predictive value despite the enriched gene expression signature in sensitive samples (Fig. 4B), suggesting low specificity. Finally, when performing the ROC analyses on all seven CCLE datasets combined, we found that the full signature did not give a good predictive AUC value (0.67, Fig. 5H) whereas the three common gene signatures gave an improved predictive AUC value (0.73, Fig. 5I). Altogether, these data suggest that despite the DNA replication initiation gene expression signature having good-to-excellent predictive AUC values for some cancers, it is not currently possible to define a single gene expression signature to accurately predict patient sensitivity to ATRi in a pan-cancer manner, thus cancer type-specific signatures are likely required for optimal biomarker development.

Overall, these analyses reveal that gene expression of origin firing factors may provide a determinant of ATRi sensitivity in several cancer types including breast and colorectal cancers that could be exploited to predict ATRi sensitivity in a clinical setting.

## Discussion

In this study, we set out to discover the mechanistic origin of differential ATRi sensitivity and have identified a determinant of sensitivity to ATR inhibition: exacerbated levels of origin firing.

In non-transformed cells (RPE1, FNE1), increasing RS rates with aphidicolin or cyclin E overexpression induced CIN and sensitivity to ATRi supporting the concept that RS and/or CIN could represent useful biomarkers for ATRi efficacy in cancer. However, ATRi sensitivity was not obviously correlated with simplistic measures of RS or CIN in cancer cell lines or samples (although *CCNE1* amplification has been described in the sensitive line HCC1806)^45^, likely due to pre-existing CIN and complex genetic backgrounds. Indeed, ATRi treatment induced RS and CIN in all breast cancer cell lines tested regardless of sensitivity. Moreover, it was previously shown that nucleoside supplementation can dampen RS^28^ and CIN^24^, but this only mildly rescued ATRi sensitivity in HCC1806. Thus, we investigated additional features of RS to understand the differences in sensitivity between the breast cancer cell lines.

We identified a correlation between slow replication fork velocity and sensitivity to ATR inhibition, though this was insufficient to predict sensitivity to ATRi. ATRi-sensitive cell lines had high expression and activity of origin firing factors, which correlated with hyper-increased origin firing in response to ATR inhibition. It has been well described before that ATRi induces excessive origin firing^9,46^ but here we suggest that the capacity for increased origin firing can influence sensitivity to ATRi. Furthermore, it is known that fork progression is slowed in the context of excessive origin firing^36^, potentially explaining the slower fork speed in sensitive cells. Reducing origin firing using CDC7 inhibition alleviated ATRi sensitivity, in line with previous work showing competing kinase activities of ATR and CDC7^47^. Similar results were obtained using CDK2 inhibitors to lower origin firing. Excessive origin firing appears to be a key driver of cell death in sensitive cell lines, while resistant cells restrain levels of origin firing at rates that do not affect their cell proliferation. It is possible that the phenomenon of “replication catastrophe” links excessive origin firing, pan-nuclear γH2AX staining, and cell death; however, robustly detecting cell death by replication catastrophe is difficult; while we use pan-nuclear γH2AX staining as an indicator, the exact mechanisms are not well known, making an unambiguous marker elusive. It would be interesting to understand whether, in our cell lines, replication catastrophe is caused by replication factor exhaustion following excessive origin firing as previously shown^33,34^.

Interestingly, BT-549, the least sensitive of the three sensitive cell lines, did not have a high-level of pan-nuclear γH2AX induced by ATRi when compared to HCC1569 and HCC1806. BT-549 also exhibits a fork velocity similar to the resistant cell line panel, low CDC45 expression and an active p53 pathway, suggesting these cells might not have reached the excess of origin firing required to induce cell death. Interestingly, we noted that this cell line demonstrated activation of p53 in contrast to the other two sensitive cell lines. It has been shown that p53 plays a role in preventing the accumulation of double-strand breaks at stalled forks, which limits the consequences of RS^48^. We speculate that p53-positive ATRi-sensitive cell lines might be protected against hyper-active origin firing-induced replication catastrophe and cell death through pan-nuclear γH2AX and are likely sensitive to ATRi through another mechanism. It would be interesting to further investigate whether wild-type p53 could play a protective role from lethal levels of origin firing upon ATRi, and whether functional p53 status could be included in further DNA replication initiation signature-based biomarker development.

We sought to determine if we could leverage our increased mechanistic understanding of ATRi sensitivity as a potential biomarker to predict ATRi sensitivity more widely. We performed transcriptomic analysis and found recurrent pathways enriched in sensitive vs resistant cancer cell lines related to mitosis, DNA replication, immune response and cell metabolism. Although we focused in this study on the RS-related pathways, we also noted in our MS that CDK1 activity was elevated in sensitive breast cancer cell lines and that mitosis-related pathways were highly enriched in sensitive breast and oesophageal squamous cancer cell lines, as well as mildly enriched in the other cancer types and in AML patient samples. The relationship between CDK1 activity, mitotic processes and sensitivity to ATR inhibition is not the subject of this study but could lead to further investigation as it could help increase the understanding of ATRi biology and unravel determinants of sensitivity. Based on the data gathered in this study, we closely examined DNA replication pathways and found that ATRi sensitivity in cell lines across different cancer types and AML patients mirrored what we observed in our breast cancer cell line panel: high expression of origin firing factors correlated with increased ATRi sensitivity. Interestingly, we also found evidence of higher origin firing kinase activities (CDC7, CDK2 specific activities) from our proteomic analyses and from AML patient samples sensitive to ATRi, which combined with transcriptomic signature analyses could provide a stronger sensitivity predictive tool in the future. Remarkably, we found that in breast and colorectal cancer cell lines, CDC45 protein expression correlated with sensitivity to ATRi. We also showed that overexpressing CDC45 in three different ATRi-resistant breast cancer cell lines mildly sensitizes them to ATRi, confirming the relationship between origin firing factors and sensitivity to ATRi.

To further explore the predictive potential of the DNA replication initiation signature, we performed ROC analyses to evaluate the sensitivity and specificity of the signature to predict the response to ATRi across the different datasets used in this study^42^. We confirmed the predictive potential of the DNA replication initiation signature in colorectal adenocarcinoma, breast adenocarcinoma, oesophageal squamous cell carcinoma and AML cell lines. We also investigated the pan-cancer predictive capacity of the signature by merging the seven cell line datasets, and revealed that the AUC, used as a prediction performance value, was not sufficiently strong. Notably, the predictive value of the three gene signature *ORC1–CDT1–MCM10* was higher and fair but still failed to predict sensitivity efficiently in some cancer types, suggesting that the full or reduced signature could be used only in certain cancer types with high predictive values (breast and colorectal cancer, oesophageal squamous cell carcinoma) or that cancer-type-specific signatures are required.

The mechanistic insight of our study provides a first step in developing multimodal biomarkers for ATRi based on increased origin firing capacity and provides information to assist in designing combination treatment strategies. For example, it may be possible to sensitize ATRi-resistant tumours by manipulating origin firing capacity, as we showed above using cell biology experiments. Interestingly, it was previously shown that the combination of CDC7i and ATRi has a synergistic effect in hepatocellular carcinoma^49^, by decreasing cancer cell viability and mouse tumour growth, suggesting this combination might be useful to sensitize resistant tumours. It has also been shown that ATRi and CDC7i can have either synergistic or antagonist effects depending on their levels of inhibition^50^ and that combining these two inhibitors might be efficient but would require optimization of inhibitor doses and monitoring of toxicity in organs with high cellular proliferation rates^51^. Of note, we found that four cell lines across our breast and colorectal cancer cell line panels were sensitized to ATRi upon CDC7 inhibition (T47D, BT-20, HT29 and SW1463). This suggests that sensitive cell lines with high origin firing capacity could respond to single agent ATRi, while resistant cell lines may be targeted using a combination of ATRi and CDC7i. Further studies are necessary to investigate which doses of inhibitors would be optimal to prove a synergistic effect on cancer cell viability, and this could be guided by predictive biomarkers of origin firing capacity.

In this study, we were limited to the few available datasets that included RNA-sequencing, MS and sensitivity (IC50) data. Therefore, future investigations on larger-scale multi-omics datasets linked to ATRi efficacy are needed to translate this knowledge into a potential biomarker suitable for clinical applications.

## Methods

### Cell culture

All cell lines were grown in their recommended media supplemented with 1× Anti-Anti (Gibco™, 15240-062) and 10% heat inactivated Fetal Bovine Serum (Gibco™, 10082147) and were incubated at 37 °C with 5% CO_2_ supply. HCC1569, HCC1806 and HCC1187 are cultured in RPMI 1640 Medium (Gibco™, 21875-034); T47D and BT-549 in RPMI supplemented with 8.3344 and 0.958 µg/mL of Insulin–Transferrin–Selenium (Gibco™, 41400-045), respectively; MDA-MB-231 in Dulbecco’s Modified Eagle Medium (Gibco™, 41966-029); BT-474 in Hybri-Care Medium (ATCC™, 46-X.) supplemented with 1.5 g/L sodium bicarbonate; and BT-20 in Minimum Essential Media (Gibco™, 21575-022). Breast cancer cell lines were obtained from AstraZeneca and the Francis Crick Institute. RKO, HT29 and SKCO1 were grown in Dulbecco’s Modified Eagle Medium (Gibco™, 41966-029) and SW1463, T84 and HT55 in F-12 Nutrient Mix (Ham, 1×) (Gibco™, No. 21765-029). The colorectal cancer cell lines were obtained from Charles Swanton’s lab at the Francis Crick Institute. All cell lines were STR-profiled and Mycoplasma tested.

FNE1 immortalized (hTERT) fallopian tube cells were obtained from the University of Miami through a material transfer agreement (MTA) and cultured in FOMI media^17^. RPE1 cells were purchased from ATCC® and grown in F-12 Nutrient Mix (Ham, 1×) (Gibco™, No. 21765-029). KTB6, KTB21, KTB34 and KTB6 immortalized (hTERT) normal breast cells were cultured in 25% Gibco™ low glucose DMEM (Gibco™, No. 12320-032) with 75% F-12 Nutrient Mix (Ham, 1×) (Gibco™, No. 21765-029) supplemented with 0.4 µg/mL hydrocortisone (Sigma-Aldrich, H0888), 5 µg/mL of Insulin (Sigma Aldrich, I5500), 20 ng/mL EGF (Sigma Aldrich, E9644), 0.5 µl/ml of Y-27632 (Rock inhibitor, ALX-279-333-M005, Enzo Life Sciences) and 4 µl/mL of Adenosine (Sigma-Aldrich, A-9001)^22^. Cells were obtained from The Susan G. Komen Tissue Bank (KTB) at Indiana University Simon Comprehensive Cancer Centre through an MTA.

### Transfection

MDA-MB-231, T47D and BT-20 cells were transfected in 6-well plates for 24 h with EGFP-C1 (Clontech #6084-1, discontinued) and EGFP-CDC45 plasmids (pEGFP-N1-Cdc45, Addgene plasmid #211426, originally generated and described in Köhler et al.^52^) using SARTORIUS jetPRIME® DNA/siRNA (co-)transfection reagent (201000003), according to the manufacturer’s instructions. The EGFP-CDC45 plasmid was obtained commercially from Addgene and propagated in *E. coli* following standard plasmid amplification and purification procedures. Cells were subsequently seeded for cell viability assays, and samples were collected for western blot analysis 48 h post-transfection to confirm CDC45 overexpression.

### Cell confluence assay

Cells were seeded in 96-well plates at a range of 3000-10,000 cells per well depending on the cell line. Twenty-four hours after seeding, the medium was changed, and cells were treated with a dose range of ceralasertib 0.05–3.2 µM. Phase contrast images were acquired every 3 h for at least 72 h using an Incucyte® S3 (Sartorius). Incucyte® software was used to apply a cellular mask for each cell line measuring the percentage cell confluence. Each experiment was performed in at least three independent replicates. For CDK2 inhibitors, optimal treatment doses were first determined such that cell proliferation or cell viability was not affected upon CDK2 inhibition per se, to allow accurate and fair measurement of the response to ATRi.

### Cell viability assay

Cell viability was assessed using the CellTiter-Glo® 2.0 Promega kit (G9241). Cells were seeded in 96-well plates and treated similarly to the confluence assays. Cells were treated for 72 h and lysed with the CellTiterGlo reagent at the end point. Samples were transferred in white flat-bottom plates (655075 from Greiner) and luminescence values were measured using FLUOstar Omega Microplate Reader (BMGLabtech). Cell viability was determined by dividing the luminescence values of treated samples by DMSO.

### Clonogenic assay

Cells were seeded at a low density in 12-well plates in duplicates. After 24 h, cells were treated with 0.8 and 3.2 µM of ATRi for 10 days before fixation with 0.5% crystal violet solution in 20% methanol. Wells were imaged using the EVOS M700 imaging system (Invitrogen) with ×4 objective images. Celleste software was used to measure the total area covered by the colonies as well as colony number and intensity. We then resuspended the crystal violet using acetic acid and measured absorbance to obtain another metric of colony density. The absorbance of treated samples was normalized by DMSO.

### Cell death assay

Cells were seeded in 96-well plates and treated similarly to the cell confluence assays. Incucyte Cytotox green dye (Sartorius) was added with ATRi at the time of treatment. Green fluorescence was then measured after 72 h using EVOS M700 imaging system (Invitrogen) using ×4 objective, before performing the CellTiterGlo assay. The Celleste 6 image analysis software (Invitrogen) was used to measure the numbers and total area of green objects. To encounter for the decrease of cell proliferation upon ATRi, a death-to-viability ratio was measured by normalizing the death object area by the cell viability data.

### Immunofluorescence

Cells were grown on glass coverslips in 6-well plates and fixed with PTEMF (0.2% Triton X-100, 0.02 M PIPES (pH 6.8), 0.01 M EGTA, 1 mM MgCl_2_, 4% formaldehyde)^18^. After blocking with 3% bovine serum albumin, cells were stained with primary antibodies against p-H2AX S139 (Millipore 05-636, 1:500) and CREST (antibodies incorporated, 15-234-0001, 1:500), and with secondary antibodies goat anti-mouse Alexa Fluor 488 (A11017 Invitrogen, 1:500) and goat anti-human Alexa Fluor 647 (A21445 Invitrogen, 1:500). DNA was stained with DAPI (4′,6-diamidino-2-phenylindole; Roche) and coverslips were mounted using Vectashield (Vector H-1000, Vector Laboratories). For EdU (5-ethynyl-20-deoxyuridine) experiments, cells were incubated for 15 min with 10 µM EdU (Thermo Fisher Scientific) prior to fixation. After fixation, EdU was stained using Click-iT EdU imaging kit (Invitrogen, C10338) as indicated in the manufacturer’s instructions.

Images were acquired using the Olympus DeltaVision RT microscope and deconvolved with SoftWorx Explorer^18^. For segregation errors, cells were imaged at ×100 objective; for micronuclei quantification at ×40 objective; and at ×20 for cell cycle analysis and γH2AX signals. Analysis of immunofluorescence images was performed using ImageJ and CellProfiler software.

### Replication fork dynamics from ONT sequencing and DNAscent

Cells were incubated with 50 µM EdU for 5 min, 50 µM BrdU for 10 min, followed by thymidine for 20 min, with washes performed between each incubation. Cells were collected by scraping into cold phosphate-buffered saline (PBS), pelleted and washed at 4 °C and frozen at −80 °C. High molecular weight genomic DNA was extracted using the QIAGEN® Puregene Core kit B (No. 1042608). The concentration, quality and length of DNA were measured using Tape Station and sample libraries were prepared using Nanopore kits SQK-LSK109 for R9.4.1 and SQK-ULK-114 for R10.4.1, quality controlled via Tape Station and sequenced with the ONT PromethION platform. Library preparation and sequencing was performed by the genomics facility of the Universities of Birmingham and Cambridge (Supplementary Data 1).

For samples sequenced on the ONT PromethION platform with a R9.4.1 nanopore, base calling of raw sequencing files (fast5 format) was performed using GUPPY (version 5.0.16) with the respective configuration (dna_r9.4.1_450bps_hac_prom.cfg). Both reads that passed and failed the base calling quality metrics were included in downstream processing. Reads were aligned to the human telomere-to-telomere (T2T) reference genome (chm13v2.0.fa) using minimap2 (version 2.24). Next, the output was converted from sam format to bam format using samtools (version 1.14). Finally, the DNAscent pipeline (version 3.1.2) with subprogrammes index, detect and forkSense was run^27^. The DNAscent forkSense output (bed file format) was processed with custom Python (version 3.11.5) scripts to obtain fork speeds and stall scores. During this study, the R9.4.1 flow cells were discontinued so experiments with HCC1806, Cdc7i and ATRi were performed on the ONT PromethION platform with a R10.4.1 nanopore. Base calling of raw sequencing files (pod5 format) and alignment (--mm2-preset map-ont option) were performed using Dorado (version 0.7.3) and the configuration dna_r10.4.1_e8.2_400bps_fast@v5.0.0 and the T2T reference genome (chm13v2.0.fa).

The DNAscent pipeline (version 4.0.3, commit 1432cf75f1e57920856f1ca465ad3bc5650ebbe9, available on https://github.com/MBoemo/DNAscent) with subprogrammes index, detect and forkSense was run, and the output was processed in the same way as described above for the R9.4.1 pore (DNAscent v3.1.2). To generate origin and fork density metrics, the total numbers of origins and forks in the sample were divided by the length of megabase sequenced to obtain a density per megabase. The DNAscent output is presented in Supplementary Data 2.

### Copy number profiles

Pod5 raw sequencing files were basecalled using Dorado v0.5.3. Adaptors were trimmed from fastq files using Porechop v0.2.4 and aligned to GRCh37 using minimap2 v2.5. Segmented copy number data was generated using 500 kb bins using QDNASeq followed by read count, mappability and GC content correction and then copy number segmentation. Absolute copy number fitting was performed using ACE using cellularity, ploidy and error estimations from the squaremodel function.

### Sample preparation and TMT labelling

Cells were washed with PBS and scraped in sodium dodecyl sulfate (SDS) 4%, 50 mM Tris HCl pH 7.4 for cell lysis and protein extraction. After sonication and lysate clearing by centrifugation, protein concentration was measured using the BCA protein assay kit and balanced to 55 µg for every sample.

To prepare the samples for proteomics analysis, isobaric filter-aided sample preparation was performed^53^. Briefly, samples were diluted in UA buffer (8 M Urea, 100 mM Tris-HCl pH 8.5 in dH_2_O) and reduced with the addition of 10 mM dithiothreitol (DTT; VWR, M109) and incubated for 30 min at room temperature. Samples were then alkylated with 55 mM IAA (iodoacetamide, VWR, 786–228) and incubated in the dark for 30 min at room temperature. Samples were loaded on Vivacon® 500, 30,000 MWCO Hydrosart filters (Sartorius, VN01H22) and centrifuged at 14,000 RCF for 20 min. Samples were washed twice with UA buffer and centrifuged at 14,000 RCF for 20 min. Then, samples were washed three times with 100 mM TEAB (triethylammonium bicarbonate, Fisher Scientific, 15215753) before a final addition of 100 μL 100 nM TEAB and 1 μg of Trypsin (Merck, T6567) to every sample before an overnight incubation at 37 °C.

Each TMTpro™ 18-plex Label Reagent (ThermoFisher Scientific, A52047) was resuspended in 100% ACN and 8 μg of TMT-Pro label was added to each respective sample filter before incubation for 1 h at 25 °C at 600 RPM. Excess TMT-Pro labels were quenched with the addition of 5% hydroxylamine and incubated for another 30 min. Peptides were eluted by centrifugation at 14,000 RCF for 20 min, and the filters were washed using 100 mM TEAB and a final wash of 30% ACN. All labelled peptides were pooled and split 10% for the total proteomics analysis and 90% for the phospho-enrichment. The pooled peptide mixture was dried with a vacuum concentrator before downstream sample preparation.

For total proteomics analysis, the TMT-labelled pool was fractionated into seven fractions using Pierce™ High pH reverse-phase fractionation kit (Life Technologies, 84868), using the manufacturer’s protocol. Fractions were dried using vacuum concentration before being resuspended in Buffer A* (2% acetonitrile (ACN), 0.1% trifluoroacetic acid, 0.5% acetic acid) for liquid chromatography–tandem MS (LC–MS/MS) analysis. For phosphoproteomics analysis, phosphopeptides were enriched for using Titansphere TiO phospho-peptide enrichment kit (GL Sciences, 5010-21308), following the manufacturer’s instructions. Eluted enriched TMT-labelled phosphopeptides were dried using vacuum centrifugation before resuspension in Buffer A* for LC–MS/MS analysis.

### MS analysis and data processing

LC–MS/MS analysis was performed on a Q Exactive-plus Orbitrap mass spectrometer coupled with a nanoflow ultimate 3000 RSL nano HPLC platform (Thermo Fisher). Dried peptide mixtures were resuspended in A* buffer. For total proteomics analysis, an equivalent of ~1 μg of protein was injected into the nanoflow HPLC. For phospho-proteomics analysis, ~90% of the total peptide mixture was injected. Samples were resolved at a flow rate of 250 nL/min on an Easy-Spray 50 cm × 75 μm RSLC C18 column (Thermo Fisher). Each run consisted of a 123 min gradient of 3–35% of Buffer B (0.1% formic acid (FA) in ACN) against Buffer A (0.1% FA in LC–MS gradient water), and separated samples were infused into the MS by electrospray ionization (ESI). Spray voltage was set at 1.95 kV, and capillary temperature was set to 255 °C. MS was operated in data-dependent positive mode, where 1 MS scan is followed by 15 MS2 scans (top 15 methods). Full-scan survey spectra (*m*/*z* 375–1500) were acquired with a 70,000 resolution for MS scans and 35,000 for the MS2 scans. A 30-s dynamic exclusion was applied.

MS raw data files were searched and quantified against a FASTA file of the Homo sapiens proteome using MaxQuant (version 2.2.2.0)^54^. Parameter groups were set to define the phospho- or total-proteome analysis and fractions were set for the total proteomics analysis. “Reporter ion MS2” type option was selected with a reporter mass tolerance of 0.003 Da. Enzyme specificity was set to “Trypsin,” allowing up to two missed cleavages. Phosphorylation (STY) was added to variable modifications for the phospho-parameter group. FDR were calculated using a reverse database search approach and were set at 1%. “Re-quantify” options were enabled. Default MaxQuant settings were used for all other parameters.

Downstream statistical analysis was performed in Perseus (version 1.6.2.3)^54^ where data was filtered for contaminants, reverse peptides and peptides only identified by site, log2 transformed, and filtered for valid values only (Supplementary Data 3–6). The Log2 data generated were used for subsequent analysis of total protein expression and KSEA.

### KSEA analysis

Phosphoproteomics Log2-transformed fold changes were translated into kinase activity using KSEA^31,55^, available on https://github.com/CutillasLab/protools2 using signor and Phosphositepus as input databases of kinase–substrate relationships^56,57^. The KSEA analysis was performed to compare a) three resistant DMSO and three sensitive DMSO to reveal basal differences of kinase activities and b) between ATRi-treated samples (1 and 24 h) and their relative DMSO to reveal kinase activities induced by the treatment. Changes in kinase activity were inferred from variations in the putative downstream targets (pdts) of each kinase. The significance of these changes were calculated using Kolmogorov–Smirnov tests^58^. *Z*-score were plotted to visualize the most enriched kinase activities between the different conditions. Volcano plots were generated using the volcano plot function on R using *z*-scores and *p* values. Data of *z*-scores, *p* values and sites of phosphorylation comparing DMSO sensitive to DMSO resistant and ATRi 1 and 24 h to DMSO generated via KSEA are presented in Supplementary Data 7–12.

### Western blotting

Cells were lysed in RIPA buffer (50 mM Tris-HCl pH 8.0, 150 mM NaCl, 1% NP-40, 0.5% sodium deoxylcholate, 0.1% SDS, 1× protease inhibitor cocktail). Protein concentration was determined using the Pierce™ BCA Protein Assay Kit (Thermo Scientific, 23227). Equal amounts of cell lysates were resolved by molecular weight using 4–12% NuPAGE™ Bis-Tris Mini Protein Gels (Invitrogen) before transferring to nitrocellulose membranes (Thermo Scientific, 88018). Membranes were blocked in 5% skimmed milk powder in TBS containing 0.1% Tween-20 (TBST) for 1 h at room temperature before probing with the following primary antibodies overnight at 4 °C: CDC45 (Cell Signaling Technology (CST), #11881; 1:1000), cyclin E1 (Abcam, ab3927; 1:1000), GFP (Roche, 11814460001; 1:2000), γH2AX (CST, #9718; 1:1000), phospho-p53 (Ser15) (CST, #9284; 1:1000), p53 (Santa Cruz Biotechnology, sc-126; 1:2000), β-actin (CST, #5125; 1:2000), β-tubulin (Abcam, ab6046; 1:2000). Horseradish peroxidase (HRP)-conjugated secondary antibody incubations (either anti-mouse IgG (CST, 7076S) or anti-rabbit IgG (Santa Cruz Biotechnology, sc-2357) were performed at room temperature for 1 h. Blots were visualized using Immobilon Forte Western HRP substrate (Millipore, WBLUF0500) and a ChemiDoc MP Imaging System (Bio-Rad). Where necessary, membranes were stripped via incubation in Restore™ Western Blot Stripping Buffer (Thermo Scientific, 21059) for 15 min at room temperature, before re-probing as above. Quantification of Western blots was performed using ImageJ.

### Transcriptomic analysis

Publicly available RNA-sequencing data of the CCLE (https://broadinstitute.org) were downloaded as transcript per million. IC50 of the cancer cell lines were obtained from the GDSC2 dataset and used to classify the cell lines as most sensitive or most resistant (IC50 lower than the first quartile or higher than the third quartile, respectively) (Supplementary Fig. 4a). GSEA was performed between the sensitive and the resistant cell line groups using GO-BP (c5.go.bp.v2024.1) gene sets as reference in the GSEA software (Broad Institute). NES and nominal *q*-values were used to identify the significantly enriched GO-term BP in sensitive cell lines (Supplementary Data 13–19).

The transcriptomic data of a panel of PDAC patient-derived primary cell lines were obtained from Dreyer and colleagues’ study^43^. We used their measured IC50 to classify the primary cell lines as sensitive (<25%) and resistant (>75%) and performed GSEA analysis, as described above (Supplementary Data 20).

The transcriptomic data of a cohort of AML patients, classified as good responders or not responders to ceralasertib, were obtained from the study by Casado and colleagues^59^. Genes were ranked in descending order of fold change (FC) and a GSEA was performed using the ranking module, between the good responders and non-responders to the ATR inhibitor (Supplementary Data 21). GO-BP (c5.go.bp.v2024.1) gene sets were used as reference.

Reactome pathway enrichment analysis by GSEA comparing sensitive and resistant samples. GSEA (Broad Institute, standard phenotype-label permutation mode) was used to calculate enrichment score (ES), NES, nominal *p* value, FDR *q* value and family-wise error rate (*p* value). Nominal *p* values were derived empirically from permutation testing, and multiple-testing correction was performed using the FDR method implemented in GSEA.

### ROC analysis

We generated ROC curves after performing LDA with LOOCV on each dataset, using R packages MASS and pROC. To evaluate the performance of the GOBP “DNA replication initiation” in predicting ATRi sensitivity on the seven CCLE datasets, we calculated pathway enrichment scores using the GSVA package in R (version 4.4.2) and performed LDA with LOOCV. LDA was implemented using the MASS package in R. ROC curves were generated using the pROC package in R. The AUC was calculated as a measure of classifier performance, where an AUC of 1.0 indicates perfect discrimination and 0.5 indicates no discriminatory ability. AUC values are included in Figs. 4 and 5.

### Statistical analysis

Statistical analyses were performed using GraphPad Prism 10.1.2 (324). To compare two datasets, we performed normality tests using Agostino and Pearson and Shapiro–Wilk tests. Paired or unpaired *t* test, Mann and Whitney and Wilcoxon rank tests were performed depending on the data distribution and type. For more than two datasets, the one-way or two-way ANOVA test was performed. In all cases, ns indicates not significant; *p < 0.05; **p < 0.01; ***p < 0.001; ****p < 0.0001. More information can be found in the figure legends.

### Reporting summary

Further information on research design is available in the Nature Portfolio Reporting Summary linked to this article.

## Supplementary information

Supplementary Information

Description of Additional Supplementary Files

Supplementary Data 1 to 25

Reporting Summary

Transparent Peer Review file

## Source data

Source Data

## Data Availability

The long-read sequencing data generated in this study have been deposited in the European Nucleotide Archive (ENA) database at EMBL-EBI under accession code PRJEB85428. The long-read DNA sequencing data generated in this study are provided in Supplementary Data 2. The mass spectrometry data generated in this study have been deposited in the ProteomeXchange Consortium via the PRIDE partner repository under accession code PXD061444 v1 [https://proteomecentral.proteomexchange.org/?pxid=PXD061444]. The total proteome Log2 filtered and phospho-total Log2 filtered data generated in this study and used for subsequent proteomic and phosphoproteomics analysis are provided in the Supplementary Data 3 and 4. Gene expression, long-read sequencing and mass spectrometry analysis raw data generated in this study are provided as Supplementary Data file 1–25. Source data are provided with this paper.
