# Supplementary material for: Replication origin firing capacity indicates ATR inhibitor sensitivity
Source: Nat Commun. 2026 Jun 19;17:7761. doi: 10.1038/s41467-026-74588-7 (PMC13434684; doi:10.1038/s41467-026-74588-7)
Supplement: Supplementary file 1 [file 41467_2026_74588_MOESM1_ESM.pdf]

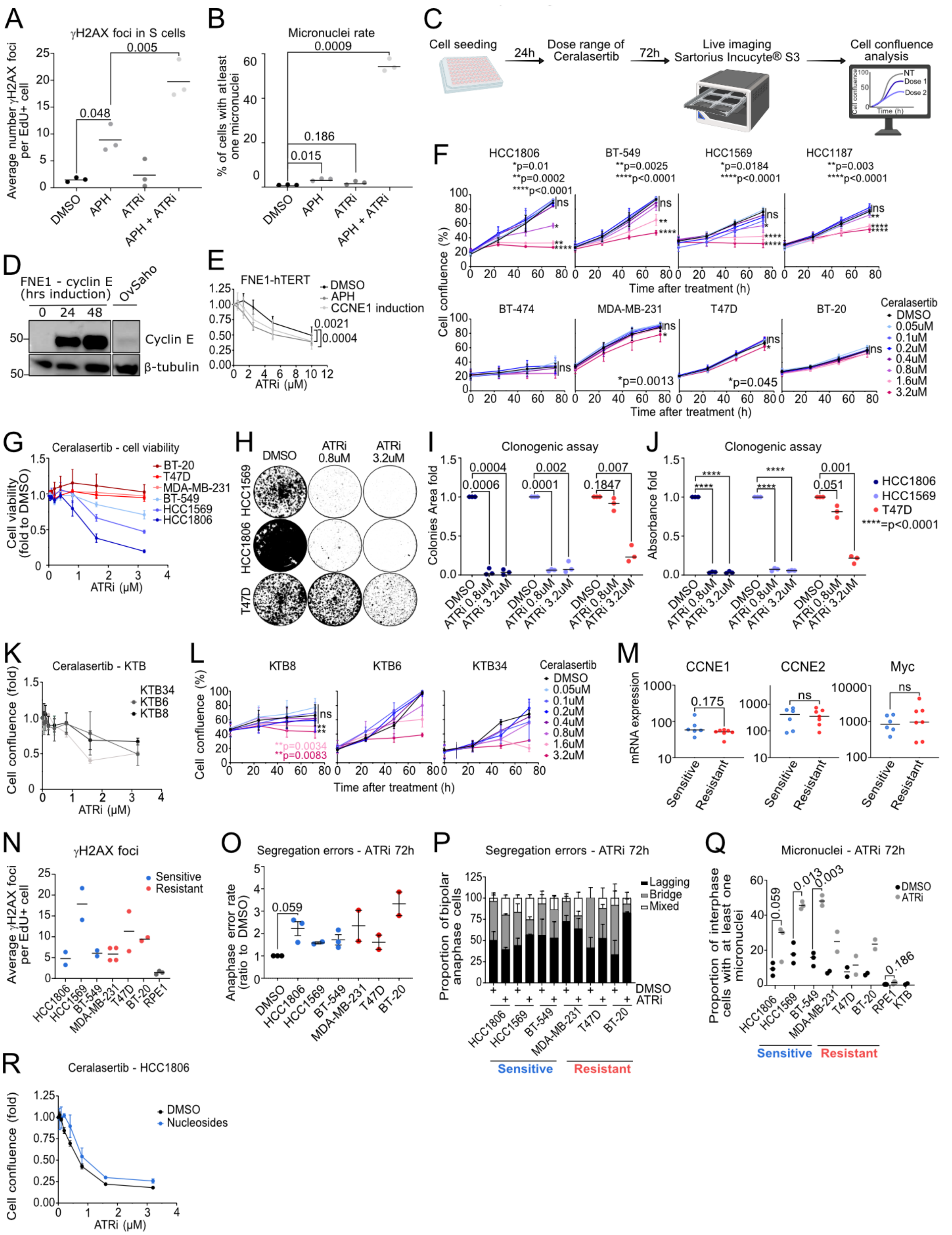

Supplementary Figure 1. (A) Quantification of the average number of γH2AX foci in replicating cells (EdU+) and (B) of the micronuclei rate in RPE hTERT cells treated with DMSO, APH 0.4uM, ATRi 0.5 µM or both for 24h. (A,B) Data are presented as mean values +/- SEM of three independent biological replicates. Two-sided paired t-test analysis. (C) Representation of the protocol used to perform the sensitivity assay to ATR inhibitor using the incucyte microscopy. Cells were treated 24h after seeding and the cell confluence was measured every few hours

for 72h. Created in BioRender. Lumeau, A. (2026) https://BioRender.com/gex8jxy. (D) Western blot of Cyclin E and β-tubulin proteins in FNE1 cells overexpressing Cyclin E, induced with 2 µg/mL of doxycycline for 0h, 24h or 48h, and Ovsaho ovarian cancer cell line. (E) Cell confluence after 72h at the doses of ceralasertib (ATRi) indicated FNE1 hTERT cells overexpressing CCNE1 by tetracycline induction 48h pre ATRi treatment. Data are presented as mean values +/- SEM of three independent biological replicates. Two-way ANOVA analysis. (F) Quantification of the confluence over time in response to different doses of ATRi in eight different breast cancer cell lines. Two-way ANOVA analysis. (G) Quantification of the cell viability of the six breast cancer cell lines in response to ATRi via CellTiterGlo assays, endpoint at 72 hours. (F,G) Data are presented as mean values +/- SEM of three independent biological replicates. (H) Clonogenic assay images of sensitive HCC1569 and HCC1806 and resistant T47D cell lines after 10 days of DMSO, 0.8 µM or 3.2µM of ATRi and quantification of the colony area (I) and the crystal violet absorbance (J) per condition. Data are presented as mean values +/- SEM of three independent biological replicates. Paired t-tests. (K) Cell confluence dose response of ceralasertib (ATRi) was measured at 72h post-treatment in three KTB immortalized cell lines and (L) quantification of the confluence over time in response to different doses of ATRi in three different KTB breast cell lines. (K,L) Data are presented as mean values +/- SEM of two (KTB6, KTB21) or three (KTB8) independent biological replicates. Two-way ANOVA analysis. (M) Quantification of the mRNA expression (normalized transcripts per million) of *CCNE1, CCNE2* and *Myc* in sensitive and resistant breast cancer cell lines using publicly available data from CCLE. Data are presented as mean values +/- SD of six sensitive and seven resistant breast cancer cell lines. Unpaired t-test analysis. (N) Quantification of the mean number of γH2AX foci per replicating cell (EdU+) in breast cancer cell lines and RPE hTERT. Results are representative of at least two independent replicates per cell line. (O) Quantification of the segregation error rate of breast cancer cell lines in response to 72 hours of 0.5 µM ATRi, fold to DMSO. Unpaired t-test. (P) Quantification of the segregation error types per anaphase, with the proportion of lagging chromosomes, chromosome bridges and mixed (lagging and bridge). (O,P) Data are presented as mean values +/- SEM of two (HCC1569, MDA-MB-231, T47D, BT-20) or three (HCC1806, HCC1569) independent biological replicates. (Q) Quantification of the micronuclei rate in breast cancer cell lines treated with 0.5 µM ATRi or DMSO for 72 hours. Data are presented as mean values +/- SEM of two (MDA-MB-231, T47D, BT-20) or three (HCC1806, HCC1569, BT-549) independent biological replicates. Paired t-test analysis. (R) Cell confluence dose response of ceralasertib (ATRi) in DMSO or 10X EmbryoMax® Nucleosides HCC1806 treated cells. Results are representative of two independent replicates. Thoughout the Figure sensitive group includes HCC1806, HCC1569, BT-549 and resistant includes MDA-MB-231, T47D and BT-20. Sensitivity was determined using the GDSC2 dataset, the top 25% IC50 of cell lines was considered resistant and the bottom 25% as sensitive. Ns, not significant; *, P < 0.05; **, P < 0.01; ***, P < 0.001; ****, P < 0.0001. Source data are provided as a Source Data file.

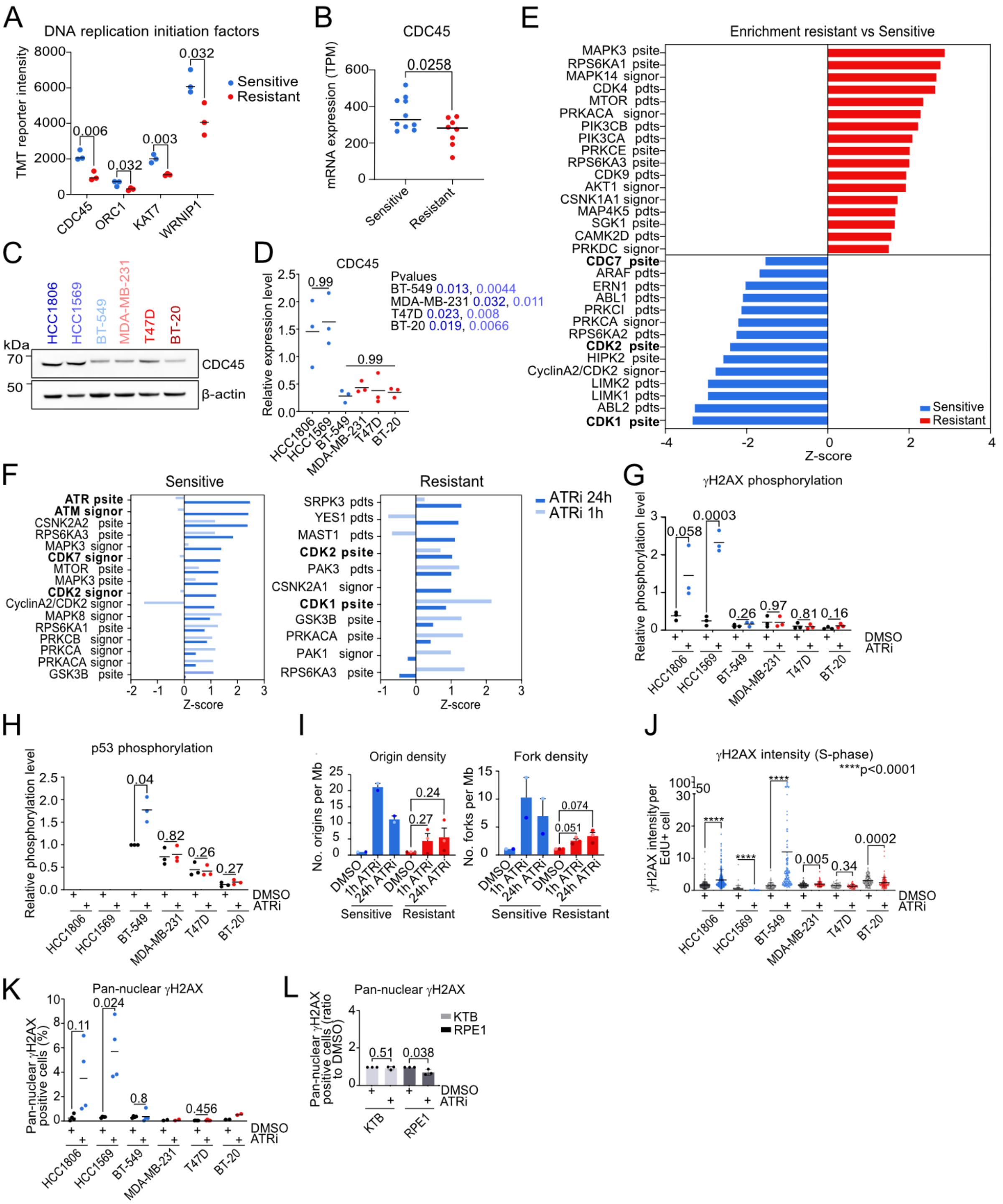

Supplementary Figure 2. (A) TMT reporter (A.U.) of DNA replication initiation factors CDC45, ORC1, KAT7 and WRNIP1 in sensitive and resistant breast cancer cell lines. Data are presented as mean values +/- SEM of three sensitive vs three resistant cell lines, experiment done in one biological replicate. Unpaired t-test analysis. (B) Quantification of the mRNA expression of CDC45 gene in sensitive and resistant breast cancer cell line using publicly available data from CCLE. Data are presented as mean values +/- SD of ten sensitive vs eight resistant cell lines. Unpaired t-test analysis. (C) Western blotting for CDC45 expression in the six breast-cancer cell lines at basal. Beta-actin was used as loading control. (D) Quantification of CDC45 expression, relative to beta-actin in the six breast cancer cell lines. For BT-549, MDA-MB-231, T47D and BT-20 the p-values of statistical tests

performed against HCC1806 are written in dark blue, and against HCC1569 in light blue. Data are presented as mean values +/- SEM of three independent biological replicates. One-way ANOVA analysis, multiple comparisons to HCC1806 or BT-549. (E) Quantification of the enriched kinase activities (z-score) in Resistant compared to Sensitive breast cancer cell lines. In red are the kinase activities enriched in resistant and in blue in sensitive cell lines. (F) Quantification of the kinase activities enriched in sensitive and resistant cell lines enriched in 0.5 µM ATRi for 1 hour or 24 hours compared to DMSO. Relative quantification of the phosphorylation of H2AX-Ser139 (G) and p53-Ser15 (H) in breast cancer cell lines after 24h of DMSO or 0.5 µM of ATRi. P-H2AX quantification relative to beta-actin expression. P-p53 quantification ratio to beta-actin expression was divided by total p53 expression ratio to beta-actin and all samples relative to BT-549 DMSO. (G,H) Data are presented as mean values +/- SEM of three independent biological replicates. Paired t-test analysis. (I) Quantification of the replication origins and forks density per megabase, fold increase to DMSO, in breast cancer cell lines treated with DMSO, 0.5 µM ATRi for 1 hour or 24 hours. Data obtained from R9 long-read DNA sequencing (see Methods), and presented as mean values +/- SEM of two sensitive vs three resistant cell lines, experiment done is one biological replicates. Paired t-test analysis. (J) Quantification of the γH2AX intensity per replicating cell (EdU+) in breast cancer cell lines treated with DMSO or 0.5 µM ATRi for 24 hours. Unpaired t-test analysis comparing DMSO and ATRi conditions. (K) Quantification of the proportion of pan-nuclear γH2AX staining in response to 24 hours DMSO or 0.5 µM ATRi in all six breast cancer cell lines and (L) in KTB and RPE1 cell lines. (K) Data are presented as mean values +/- SEM of two (MDA-MB-231, BT-20) or four (HCC1806, HCC1569, T47D) independent biological replicates and in three independent experiments for (L). Paired t-test analysis. Sensitive group includes HCC1806, HCC1569, BT-549 and resistant includes MDA-MB-231, T47D and BT-20 throughout the Figure. Ns, not significant; ****, $P < 0.0001$. Source data are provided as a Source Data file.

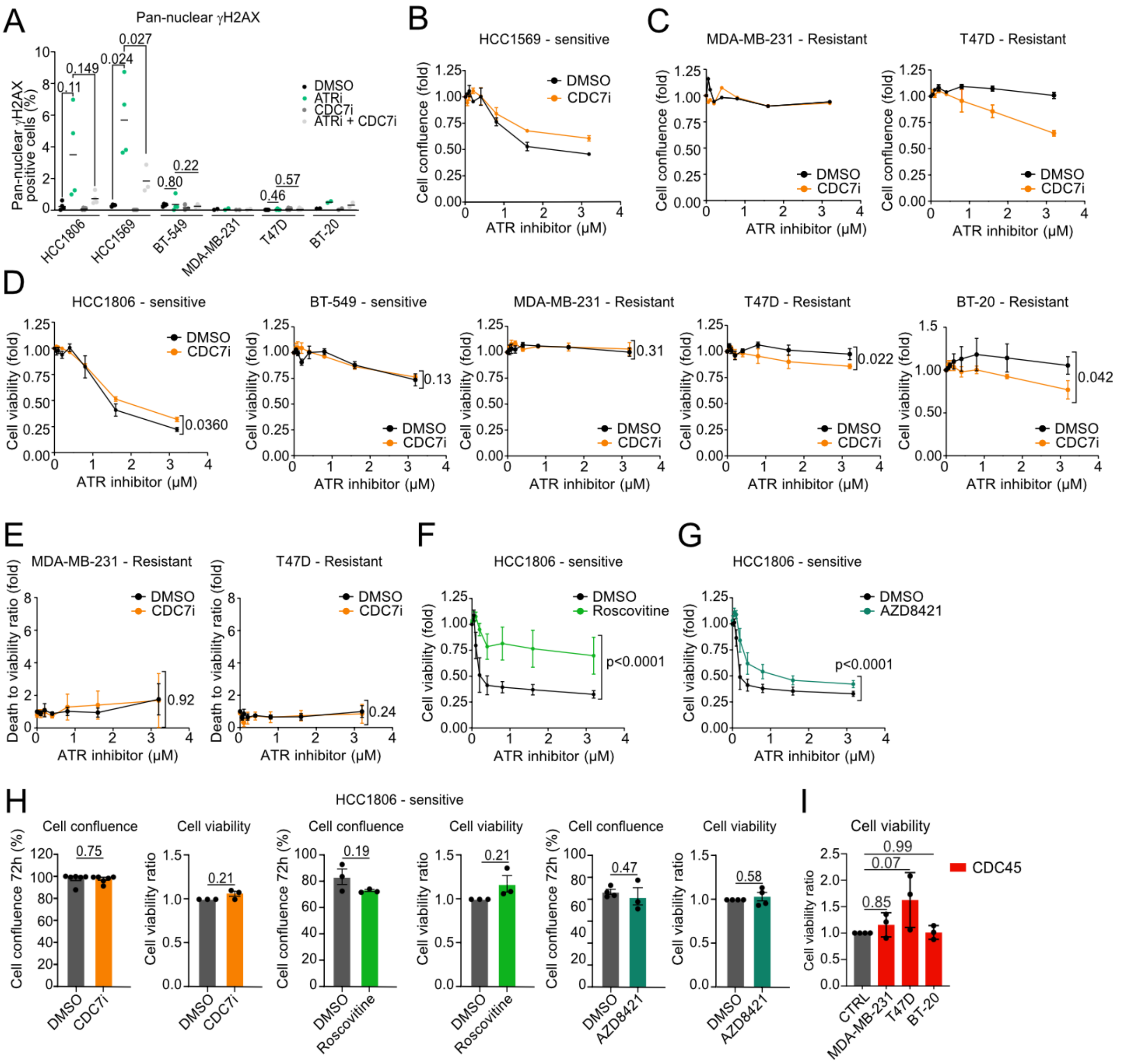

Supplementary Figure 3. (A) Quantification of the proportion of pan-nuclear γH2AX staining in all six breast cancer cell lines in response to 24 hours DMSO, 0.5 µM ATRi, 1 µM CDC7i or both treatments. Data are presented as mean values +/- SEM of two (MDA-MB-231, BT-20) or four (HCC1806, HCC1569, BT-549, T47D) biological replicates. Paired t-test analysis. Quantification of the cell confluence fold in response to ATRi fold to DMSO after 72 hours of treatment, in sensitive HCC1569 (B) and resistant MDA-MB-231 and T47D cell lines (C). Results represent one (MDA-MB-231) or two (HCC1569, T47D) independent replicates. (D) Quantification of cell viability fold in response to ATRi fold to DMSO after 72 hours of treatment, in sensitive HCC1806, BT-549 and in resistant MDA-MB-231, T47D and BT-20. Data are presented as mean values +/- SEM in three independent replicates, two-way ANOVA analysis. (E) Quantification of the cell death to viability ratio in response to ATRi fold to DMSO after 72 hours of treatment in resistant MDA-MB-231 and T47D. Data are presented as mean values +/- SEM of three independent replicates, two-way ANOVA analysis. Ns = non-significant. Cell viability of HCC1806 treated with ATRi in combination with 10 µM roscovitine (F) or 1 µM AZD8421 (G) for 72h. Data are presented as mean values +/- SEM of three independent replicates, two-way ANOVA analysis. (H) Cell confluence and cell viability of HCC1806 sensitive cells treated with DMSO or 10 µM roscovitine or 1 µM AZD8421 for 72 hours. Data are

presented as mean values +/- SEM of three independent replicates, paired t-test analysis, ns = non-significant. (I) Cell viability of MDA-MB-231, T47D and BT-20 overexpressing CDC45 normalized to CTRL. Data are presented as mean values +/- SEM of three independent replicates, one-way ANOVA analysis, ns = non-significant. Source data are provided as a Source Data file.

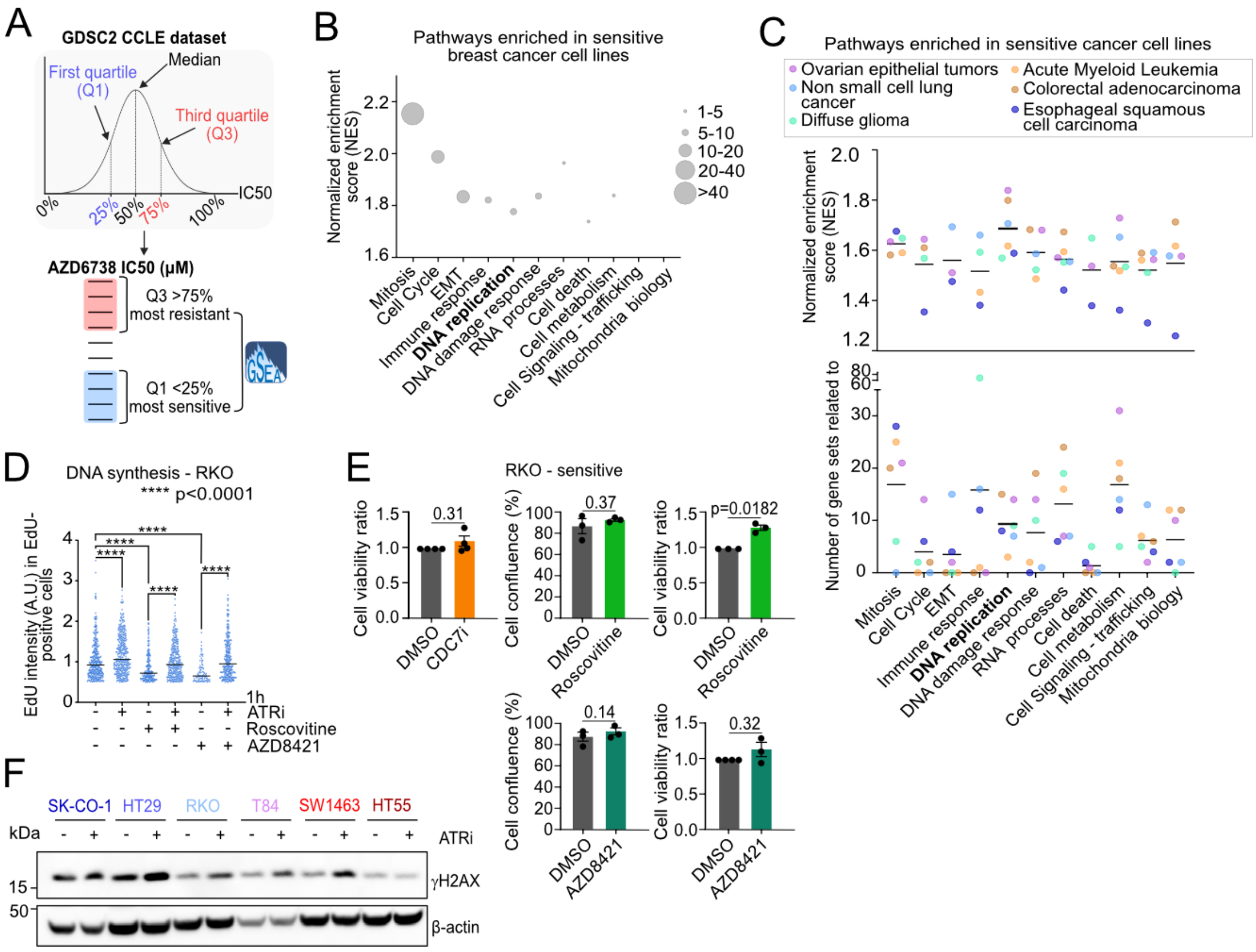

Supplementary Figure 4. (A) Schematic of quartiles method to identify the groups of sensitive and resistant cell lines from the GDSC2 IC50 of AZD6738, used for gene set enrichment analysis. Created in BioRender. Lumeau, A. (2026) https://BioRender.com/dkw0xyv. (B) Gene Set Enrichment Analysis (GSEA) analysis of publicly available transcriptomic data showing the normalized enrichment score (NES) and the number of pathways enriched per gene ontology (GO) biological processes in sensitive vs resistant breast cancer cell lines. (C) GSEA of publicly available transcriptomic data showing the NES and the number of pathways enriched GO biological processes in sensitive vs resistant cancer cell lines. (D) Quantification of the EdU intensity in replicating cells (EdU+) in sensitive RKO cells treated with DMSO, 1 µM roscovitine, 1 µM AZD8421 or the combinations with 0.5 µM ATRi for 1 hour. EdU intensity is used as a metric for activation of origin firing. Data are presented as mean values +/- SD of 200 to 500 EdU positive cells, experiment performed in two independent replicates. Mann and Whitney test analysis. (E) Cell confluence and cell viability of RKO sensitive cells treated with DMSO or 1 µM roscovitine or 1 µM AZD8421 for 72 hours. Data are presented as mean values +/- SEM of three independent replicates, paired t-test analysis, ns = non-significant. (F) Western blotting for H2AX-Ser139 in response to 24 hours of DMSO or 0.5 µM ATRi in the six colorectal cancer cell lines. Beta-actin was used as loading control. Blot representative of three independent experiments. Source data are provided as a Source Data file.

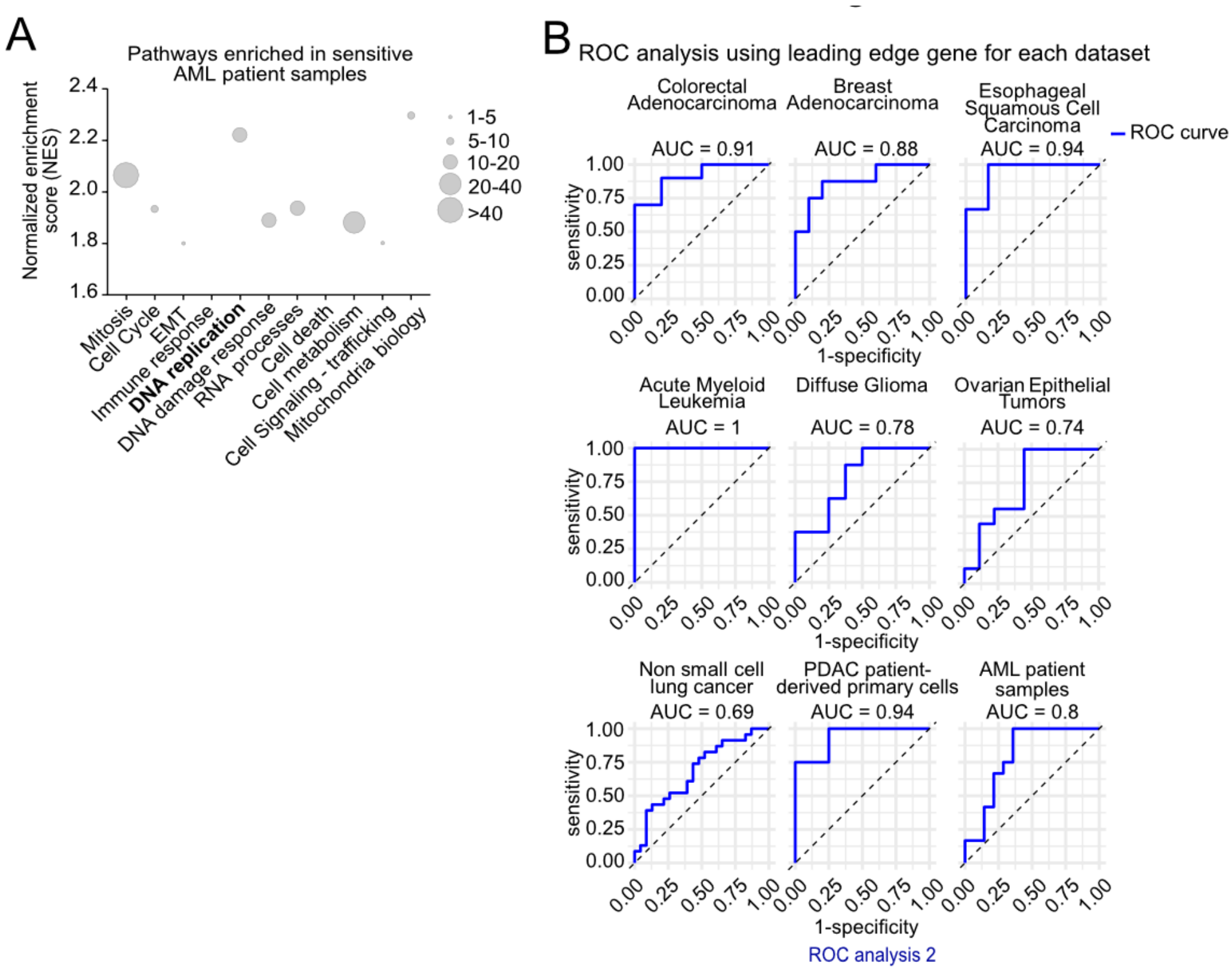

Supplementary Figure 5. (A) Gene Set Enrichment Analysis (GSEA) analysis of acute myeloid patient (AML) transcriptomic data showing the normalized enrichment score (NES) and the number of pathways enriched, per gene ontology (GO) biological processes, in predicted good responders compared to non-responders AML samples based on patient-derived primary cells sensitivity. (B) ROC curve analysis demonstrates performance of using the leading-edge genes of the DNA replication initiation pathway per pathway to predict ATRi sensitivity, in the seven CCLE datasets. ROC curves were generated following LDA with leave-one-out cross-validation. AUC values were measured for each ROC analysis. For each dataset the number of samples in sensitive and resistant groups are the same as in Figure 4 and 5. Source data are provided as a Source Data file.
