# Supplementary material for: Replication origin firing capacity indicates ATR inhibitor sensitivity
Source: Nat Commun. 2026 Jun 19;17:7761. doi: 10.1038/s41467-026-74588-7 (PMC13434684; doi:10.1038/s41467-026-74588-7)
Supplement: Supplementary file 2 [file 41467_2026_74588_MOESM2_ESM.pdf]

Description of Additional Supplementary Files

Supp Data 1: long-read sequencing protocols

Supp Data 2: Long read DNA sequencing data analyzed by DNAscent

Supp Data 3: Total proteome Log2 filtered

Supp Data 4: Phospho-total data Log2 filtered

Supp Data 5: Total proteome ANOVA analysis, post hoc significant pairs (FDR 0.05) across all conditions

Supp Data 6: Total proteome ANOVA analysis, post hoc significant pairs (FDR 0.05) enriched in DMSO sensitive DMSO resistant

Supp Data 7: KSEA analysis z-scores DMSO resistant vs DMSO sensitive

Supp Data 8: KSEA analysis p-values DMSO resistant vs DMSO sensitive

Supp Data 9: KSEA analysis sites DMSO resistant vs DMSO sensitive

Supp Data 10: KSEA analysis z-scores ATRi vs DMSO

Supp Data 11: KSEA analysis p-values ATRi vs DMSO

Supp Data 12: KSEA analysis sites ATRi vs DMSO

Supp Data 13: GSEA analysis of gene ontology biological processes enrichment in sensitive vs resistant breast cancer cell lines (CCLE public data)

Supp Data 14: GSEA analysis of gene ontology biological processes enrichment in sensitive vs resistant ovarian epithelial tumour cell lines (CCLE public data)

Supp Data 15: GSEA analysis of gene ontology biological processes enrichment in sensitive vs resistant colorectal adenocarcinoma cell lines (CCLE public data)

Supp Data 16: GSEA analysis of gene ontology biological processes enrichment in sensitive vs resistant non small cell lung cancer cell lines (CCLE public data)

Supp Data 17: GSEA analysis of gene ontology biological processes enrichment in sensitive vs resistant oesophageal squamous cell carcinoma cell lines (CCLE public data)

Supp Data 18: GSEA analysis of gene ontology biological processes enrichment in sensitive vs resistant diffuse glioma cell lines (CCLE public data)

Supp Data 19: GSEA analysis of gene ontology biological processes enrichment in sensitive vs resistant acute myeloid leukaemia cell lines (CCLE public data)

Supp Data 20: GSEA analysis of gene ontology biological processes enrichment in sensitive vs resistant PDAC patient-derived primary cell lines (Dreyer et al. 2021)

Supp Data 21: GSEA analysis of gene ontology biological processes enrichment in sensitive vs resistant acute myeloid leukemia patient samples (Casado et al. 2023)

Supp Data 22: Leading edge genes CCLE breast cancer cell lines

Supp Data 23: Leading edge genes CCLE cell lines

Supp Data 24: Leading edge genes pancreatic cancer derived primary cells (Dreyer et al. 2021)

Supp Data 25: Leading edge genes AML patient samples
