# Supplementary material for: Replication origin firing capacity indicates ATR inhibitor sensitivity
Source: Nat Commun. 2026 Jun 19;17:7761. doi: 10.1038/s41467-026-74588-7 (PMC13434684; doi:10.1038/s41467-026-74588-7)
Supplement: Supplementary file 3 [file 41467_2026_74588_MOESM4_ESM.pdf]

nature portfolio

Corresponding author(s): Sarah McClelland
Last updated by author(s): Jun 4, 2026

Reporting Summary

Nature Portfolio wishes to improve the reproducibility of the work that we publish. This form provides structure for consistency and transparency in reporting. For further information on Nature Portfolio policies, see our Editorial Policies and the Editorial Policy Checklist.

Statistics

For all statistical analyses, confirm that the following items are present in the figure legend, table legend, main text, or Methods section.

n/a	Confirmed	
☐	☒	The exact sample size (*n*) for each experimental group/condition, given as a discrete number and unit of measurement
☐	☒	A statement on whether measurements were taken from distinct samples or whether the same sample was measured repeatedly
☐	☒	The statistical test(s) used AND whether they are one- or two-sided<br>*Only common tests should be described solely by name; describe more complex techniques in the Methods section.*
☒	☐	A description of all covariates tested
☒	☐	A description of any assumptions or corrections, such as tests of normality and adjustment for multiple comparisons
☐	☒	A full description of the statistical parameters including central tendency (e.g. means) or other basic estimates (e.g. regression coefficient) AND variation (e.g. standard deviation) or associated estimates of uncertainty (e.g. confidence intervals)
☐	☒	For null hypothesis testing, the test statistic (e.g. *F*, *t*, *r*) with confidence intervals, effect sizes, degrees of freedom and *P* value noted<br>*Give P values as exact values whenever suitable.*
☒	☐	For Bayesian analysis, information on the choice of priors and Markov chain Monte Carlo settings
☒	☐	For hierarchical and complex designs, identification of the appropriate level for tests and full reporting of outcomes
☒	☐	Estimates of effect sizes (e.g. Cohen's *d*, Pearson's *r*), indicating how they were calculated

Our web collection on statistics for biologists contains articles on many of the points above.

Software and code

Policy information about availability of computer code

Data collection	Immunofluorescence images were generated using Olympus DeltaVision RT microscope. Cell confluence data were generated using the Sartorius Incucyte® S3 microscope. Clonogenic and cell death assays data were generated using the EVOS M700 imaging system (Invitrogen). Cell viability assay data were generated using FLUOstar Omega Microplate Reader (BMGLabtech). Peptides and phospho-peptides were TMT-labelled and run on a Liquid Chromatography-Tandem Mass Spectrometer. Long-read DNA sample libraries were prepared using Nanopore kits (SQK-LSK109, SQK-ULK-114) and sequenced with the ONT PromethION platform. Western Blot images were generated using the ChemiDoc MP Imaging System (Bio-Rad).
Data analysis	Immunofluorescence images were quantified using CellProfiler software. Dose response curves were analyzed using the Sartorius Incucyte® S3 analysis software. Clonogenic and cell death analysis were performed using the Celleste 6 image analysis software (Invitrogen). Phosphoproteomics Log2 transformed fold changes were translated into kinase activity using Kinase Enrichment Analysis (KSEA) using signorThe Celleste 6 image analysis software (Invitrogen) and Phosphositepus as input databases of kinase-substrate relationships (https://github.com/CutillasLab/protools2).The DNAscent pipeline (version 3.1.2 for R9 and 4.0.3 for R10) with subprogrammes index, detect and forkSense was run and processed to generate fork dynamics data (https://github.com/MBoemo/DNAscent). Gene Set Enrichment analysis were performed using Gene Ontology - Biology Processes gene sets as reference in the GSEA software (c5.go.bp.v2024.1). Western Blot images were quantified using ImageJ. Statistical analysis were performed using GraphPad Prism version 10.

For manuscripts utilizing custom algorithms or software that are central to the research but not yet described in published literature, software must be made available to editors and reviewers. We strongly encourage code deposition in a community repository (e.g. GitHub). See the Nature Portfolio guidelines for submitting code & software for further information.

Data

Policy information about availability of data

All manuscripts must include a data availability statement. This statement should provide the following information, where applicable:

- Accession codes, unique identifiers, or web links for publicly available datasets
- A description of any restrictions on data availability
- For clinical datasets or third party data, please ensure that the statement adheres to our policy

Gene expression, long-read sequencing and mass spectrometry analysis raw data generated in this study can be found in the file 'Supplementary Tables' Excel spreadsheet. Raw mass spectrometry data files were deposited on ProteomeXchange Consortium via the PRIDE partner repository under the accession number PXD061444 (Username: reviewer_pxd061444@ebi.ac.uk, Password: Yxjj5X7U0eH3). Long-read DNA sequencing data were deposited in the European Nucleotide Archive (ENA) at EMBL-EBI under accession number PRJEB85428.

Research involving human participants, their data, or biological material

Policy information about studies with human participants or human data. See also policy information about sex, gender (identity/presentation), and sexual orientation and race, ethnicity and racism.

Reporting on sex and gender	Not applicable.
Reporting on race, ethnicity, or other socially relevant groupings	Not applicable.
Population characteristics	Not applicable.
Recruitment	Not applicable.
Ethics oversight	Not applicable.

Note that full information on the approval of the study protocol must also be provided in the manuscript.

Field-specific reporting

Please select the one below that is the best fit for your research. If you are not sure, read the appropriate sections before making your selection.

☒ Life sciences ☐ Behavioural & social sciences ☐ Ecological, evolutionary & environmental sciences

For a reference copy of the document with all sections, see nature.com/documents/nr-reporting-summary-flat.pdf

Life sciences study design

All studies must disclose on these points even when the disclosure is negative.

Sample size	For in vitro work, we used 3 sensitive and 3 resistant breast cancer cell lines, to be able to perform statistical group comparative analysis (3 vs 3). For the genomic and transtriptomic analysis, we used the maximum number of cell lines from which data were publicly available, to increase the rational and statistical power of the study.
Data exclusions	No data were excluded.
Replication	We repeated the experiments with a number of biological repeats indicated in the figure legends.
Randomization	Not applicable.
Blinding	Not applicable.

Reporting for specific materials, systems and methods

We require information from authors about some types of materials, experimental systems and methods used in many studies. Here, indicate whether each material, system or method listed is relevant to your study. If you are not sure if a list item applies to your research, read the appropriate section before selecting a response.

Materials & experimental systems

n/a	Involved in the study	
☐	☒	Antibodies
☐	☒	Eukaryotic cell lines
☒	☐	Palaeontology and archaeology
☒	☐	Animals and other organisms
☒	☐	Clinical data
☒	☐	Dual use research of concern
☒	☐	Plants

Methods

n/a	Involved in the study	
☒	☐	ChIP-seq
☒	☐	Flow cytometry
☒	☐	MRI-based neuroimaging

Antibodies

Antibodies used

For immunofluorescence, the following antibodies were used: p-H2AX S139 Millipore 05-636, CREST antibodies incorporated, 15-234-0001, secondary antibodies goat anti-mouse Alexa Fluor 488 A11017 Invitrogen and goat anti-human Alexa Fluor 647 A21445 Invitrogen. For Western Blots, the following additional antibodies were used: CDC45 (Cell Signaling Technology (CST), #11881), cyclin E1 (Abcam, ab3927), GFP (Roche, 11814460001), γH2AX (CST, #9718), phospho-p53 (Ser15) (CST, #9284), p53 (Santa Cruz Biotechnology, sc-126), β-actin (CST, #5125), β-tubulin (Abcam, ab6046), horseradish peroxidase (HRP)-conjugated secondary antibodies anti-mouse IgG (CST, 7076S) and anti-rabbit IgG (Santa Cruz Biotechnology, sc-2357).

Validation

The antibodies were validated based on the manufacturer's instructions and published in previous work.

Eukaryotic cell lines

Policy information about cell lines and Sex and Gender in Research

Cell line source(s)

All breast cancer cell lines were obtained from AstraZeneca through collaboration: HCC1806, HCC1569, BT549, HCC1187, BT474, T47D, BT20, MDA-MB-231. KTB cells were obtained from The Susan G. Komen Tissue Bank (KTB) at Indiana University Simon Comprehensive Cancer Centre (IUSCCC) through an MTA (material transfer agreement). FNE1 immortalized (hTERT) fallopian tube cells were obtained from University of Miami through an MTA. RPE1 (hTERT) cells were obtained as published in our previous study Worrall et al. 2018. Colorectal cancer cell lines were obtained from the Francis CRICK Institute: SKCO1, RKO, HT55, T84, SW1463, HT55.

Authentication

All cell lines were STR profiled

Mycoplasma contamination

All cell lines were tested for mycoplasma monthly.

Commonly misidentified lines
(See ICLAC register)

None of the cell lines used in this study were on the ICLAC register.

Plants

Seed stocks

Not applicable.

Novel plant genotypes

Not applicable.

Authentication

Not applicable.
