# Supplementary material for: Replication origin firing capacity indicates ATR inhibitor sensitivity
Source: Nat Commun. 2026 Jun 19;17:7761. doi: 10.1038/s41467-026-74588-7 (PMC13434684; doi:10.1038/s41467-026-74588-7)
Supplement: Supplementary file 4 [file 41467_2026_74588_MOESM5_ESM.pdf]

nature portfolio

Peer Review File

Replication origin firing capacity indicates ATR inhibitor sensitivity

Corresponding Author: Professor Sarah McClelland

This file contains all reviewer reports in order by version, followed by all author rebuttals in order by version.

Version 0:

Reviewer comments:

Reviewer #1

(Remarks to the Author)
ATRi have already been shown to compromise cell cycle checkpoints and hasten premature mitosis, impair replication fork stability and derepress the firing of latent replication origins. This biology has led to the idea that ATR inhibitors have potential in treating cancers with certain types of replication fork stress. However, measuring RFS in cancers is complex and therefore most existing ATRi trials either use genomic surrogates of RFS to select patients for treatment or, sadly, have patient stratification criteria based on histology or resistance to other agents, presuming that these features are also surrogates of RFS. The result of this is that despite nearly a decade of clinical development, not one ATRi has yet been approved for the treatment of cancer. Here Lumeau et al reveal that a subset of breast tumor cell lines and AML patient samples have higher expression of DNA replication initiation factors and increased origin firing upon ATRi exposure and that ATRi sensitivity is causally associated with origin firing rates and decreased by Cdc7 inhibition. The authors argue that this information could lead to the development of multimodal clinical biomarkers for ATRi treatment.

Major comments:

1. Fig 1 J – is this analysis consistent with the conclusion that RS-CAN patterns predict ATRi sensitivity ? Cluster 3 has a very small n with a large variance in IC50 and is ns when compared to cluster 2. I'm not sure how valid this conclusion is based on this analysis.
2. Figure 2 is interesting but it is challenging to reject hypotheses when a sample size (i.e. number of cell lines analyzed) is very small and also when these have so many differences between them, including ER status. I think authors have to acknowledge that whilst the data does not support the idea that the ATRi induced RFS associated chromosomal instability correlates with ATRi sensitivity, they also have little power to reject these hypotheses either.
3. Figure 3 L, M – these are very modest differences in ATRi sensitivity shown, even by comparison to what others have shown the antagonism between Cdc7 and ATRi to be (see below).
4. If Cdc7 activity is different in ATRi sensitive vs resistant cell lines can authors show that Cdc7i have differing effects in ATRi resistant cell lines ?
5. I think authors have to acknowledge beyond the extent that they do that the interaction between Cdc7 and ATR inhibition is a well-known and understood phenomenon (e.g. PMID: 37378325 PMID: 34663432 PMID: 32877678 PMID: 31209037 PMID: 29123096 PMID: 12535533).
6. In figure 4 authors use GSEA to pull out enriched pathways associated with ATRi sensitivity. Although DNA replication came out as an enriched process, it is important to understand how the genes in this signature actually relate to the biology described in figure 3. For example, which genes in the replication signature are those most highly correlated with ATRi sensitivity?
7. Following on from point (6) it might be important to show whether the relationship between the dna replication initiation signature and ATRi sensitivity is stronger than relationships with previously identified biomarkers of ATRi sensitivity.
8. ATRi other than ceralasertib have been described and have data in large scale tumor cell line screens. For the sake of rigor, can the authors show similar relationships exist with ATRi sensitivity in these other datasets?
9. Ideally, predictive biomarkers need to be binary classifiers (i.e. based on the analysis of the biomarker, does the patient fall into the "sensitive/should receive the drug or resistant/shouldn't receive the drug classes). Can the authors carry out a more formal ROC analysis or similar to test the sensitivity and specificity performance of a metagene of replication signature?
Minor comments:

1. Fig 1. Is it necessary to show that Cyclin E over expression causes a very minor increase in ATRi sensitivity, given this has been known since 2011 (PMID: 21552262) ?
2. Fig 1 A, B ANOVA stats on the entire dose response curve might be more appropriate than single dose t tests.

Reviewer #2

(Remarks to the Author)
In this manuscript Lumeau et al. investigate what makes cancer cells sensitive to ATR inhibitors (ATRi). Several of these inhibitors are in clinical trials, but there is currently no reliable biomarker to predict tumor sensitivity to them. Hence, the manuscript tackles a urgent clinical need and provides important clues on an active and important research area.

The authors initially focus on breast cancer cell lines, establishing a generic (and highly expected) link between drug- or oncogene-induced replication stress (RS) and ATRi sensitivity, although the correlation with oncogene expression is not very strong. They also report no clear correlation of ATRi sensitivity with other typical RS markers, such as general levels of pre-existing or ATRi-induced chromosomal rearrangements, fork slowdown or fork stalling, excluding these functional readouts as possible predictive biomarkers of ATRi responsiveness. However, what they do report based on in-depth genomic investigations on sensitive and resistant breast cancer lines is a strong correlation between ATRi sensitivity and a specific pattern of copy number abnormalities, linked to the propensity of the sensitive lines to unleash replication origin firing. The authors consolidate this evidence by showing that sensitive cell lines have increased levels of initiation factors (CDC45) and/or specific marks of origin firing such as pMCM2, which leads to higher DNA damage and cell death upon ATR inhibition. Furthermore, the genotoxic effects of ATRi in the sensitive lines are partially rescued by counteracting the increased origin firing with a CDC7 inhibitor. In the final part of the manuscript, the authors try to extend the pan-cancer relevance of their findings by analyzing publicly available drug sensitivity, transcriptomic and phosphoproteomic datasets in various tumor types and more specifically in an AML patient cohort, observing a generic correlation between sensitivity to ATRi and increased DNA replication initiation, via transcriptomic and phosphoproteomic signatures. Based on these data, the authors suggest that markers of unleashed origin firing could be explored as general predictive biomarkers of ATRi sensitivity in cancer.

The manuscript is generally well written, and some of the conclusions are well supported and discussed, which may in principle supports publication in Nature Communications after appropriate revisions. While – with few exceptions (see below) – the results in breast cancer cell lines are overall convincing, the work on other cancer types is clearly under-developed, questioning how far the proposed biomarker could be applicable to all cancers. Title, abstract and discussion are currently pushing this “pan-cancer” aspect quite a lot, which should be corrected unless the authors manage to significantly consolidate the second part of their manuscript. A few additional concerns should be addressed, also on the first part of their results in breast cancer cell lines.

Major points:

1. Sensitivity throughout the manuscript was solely assessed by cell confluence. Given that cell sensitivity is key to the conclusions of this manuscript, the authors should exploit a different, independent mean to assess cell survival (e.g. clonogenic assays) and (hopefully) confirm all key results in their manuscript. Moreover, it would be very important to distinguish whether the effects on cellular growth are due to cytostatic (cell cycle arrest) or cytotoxic (cell killing) effects.

2. In Figure 2F-I and Extended Figure 2B, the authors should further discuss the origin of the increased CIN upon ATRi in the resistant cell lines. In this respect, how do the authors explain that ATRi-sensitive and resistant cell lines present similar levels of CIN upon ATRi, but only the sensitive ones exhibit increased DNA damage signaling upon ATRi (Figure 3E-F)? Moreover, in Figure 2I the authors claim that no significant differences in CIN levels by micronuclei formation can be observed between sensitive and resistant cell lines. On the contrary ATRi induces up to 40% of cells with micronuclei in sensitive cell lines while the increase in the resistant only up to 20%. Statistics are not shown comparing those 2 conditions, but a trend is clearly observable. These statements seem overall not well supported and may need to be far less dogmatic, providing statistical significance data for each specific comparison. More in general, the authors should systematically calculate and display absolute p-values (as opposed to * or ns) to support key statements throughout their manuscript.

3. In Figure 3D, the authors show higher levels of MCM2 phosphorylation (indicative of higher origin activation) in sensitive compared to resistant breast cancer cell lines in basal conditions. On the contrary, Figure 3G shows no differences in origin firing levels between sensitive and resistant lines in the same conditions. How do the authors explain this observation, which seems clearly at odd with one of the key claims of the manuscript?

4. The effect of the CDC7 inhibitor on cell survival upon ATRi is at best minor (Figure 3L-M). By no means it is sufficient to rescue cell survival to the level observed in resistant lines (see ED Figure 3). The authors should further support this key claim by a) preventing origin firing by an alternative, independent inhibitor (e.g. Roscovitine? Please note that CDK2 seemed to have even higher enrichment than Cdc7 in their initial screen); b) displaying that – besides the mild effects on survival – also other key readouts of ATRi genotoxicity (DDR markers or DNA damage itself) are suppressed by limiting origin firing. In fact, in Figure 3O, the authors claim a change in % of sensitive cells with pan-nuclear gH2AX when treated with ATRi, but statistical significance says the contrary. Overall, providing additional lines of support to this central claim of the manuscript seems essential to grant publication of the study.

5. Figure 4 is overall unconvincing, extending their previous finding from breast cancer to different cancer types. The authors limited this analysis purely to transcriptomics and the enrichment of the DDR/replication pathway (over other pathways) is not clear (Figure 4C). and no effort was invested confirming the relevance of origin firing (e.g. CDC7) in non-breast tumours. Further support to the relevance of origin firing control seems particularly important for AML, on which the authors focused for the data in Figure 5.

6. The authors suggest in the discussion that sensitizing ATRi-resistant tumors could be possible by manipulating their origin firing capacity. However, their results suggest that sensitization would be achieved by unleashing (rather than preventing) origin firing. This seems contradictory to previous findings cited by the authors (Guo et al.) and also to what the authors claim themselves in the second last paragraph of their discussion. Once/if the authors manage to consolidate the relevance of origin firing levels for ATRi sensitivity, this aspect of their Discussion would need to be adjusted for clarity.

Minor points:

7. In figure 2C, it is not clear whether the displayed fork speed is in presence or absence of ATRi

8. The authors should validate their results in Figures 3B (CDC45), 3D (MCM2 phosphorylation) and 3F (ATM activation) by western blot for the specific markers.

9. In Figure 3I-K, how do the authors explain a decrease in EdU incorporation upon CDC7i, given that the decrease in OF is compensated by an increase in FR?

10. In figure 4C bottom panel, Mitosis is missing the median bar.

11. The authors should always indicate which sensitive and resistant cell lines they use. For instance, in Figure 3O this is not clear. It would also be important to include a Figure to visually explain how the different cell lines for different cancer cell types were segregated in sensitive and resistant from the GDSC2 dataset.

Reviewer #3

(Remarks to the Author)
The manuscript ”High levels of DNA replication initiation factors indicate ATRi sensitivity via excessive origin firing” by Lumeau et al. aims to identify prognostic factors indicating sensitivity to ATR inhibition. Since multiple ATR inhibitors are currently undergoing clinical trials, studies like this one are urgently needed to better select the patients who would most benefit from such therapies. Here, the authors show that high expression of origin firing factors predicts increased sensitivity to ATR inhibition due to increased background rate of origin firing. The authors demonstrate this in both breast cancer cell lines and AML and consistently show very convincing correlations between the increased expression of the origin firing factors and ATRi sensitivity. I believe this is a very valuable study, and should be published, but it would strongly benefit from some direct confirmation experiments.
Specifically, to confirm that the overexpression of replication initiation proteins directly results in ATRi sensitivity, the authors should overexpress some of those factors in ATRi-resistant cells and confirm that the sensitivity is induced. CDC45 overexpression is long known to induce origin firing on its own (PMID: 26919204), along with some other proteins (PMID: 36516748), any of these can be used.
Also, the authors provide extensive supplementary tables, but they are not fully annotated, and not all of thems are referenced in the text of the manuscript. Perhaps, they could be referenced in the methods if the data in them are not directly discussed in the manuscript (for example, sup table 5 vs sup table 6).

Reviewer #4

(Remarks to the Author)
I co-reviewed this manuscript with one of the reviewers who provided the listed reports. This is part of the Nature Communications initiative to facilitate training in peer review and to provide appropriate recognition for Early Career Researchers who co-review manuscripts.

Reviewer #5

(Remarks to the Author)
I co-reviewed this manuscript with one of the reviewers who provided the listed reports. This is part of the Nature Communications initiative to facilitate training in peer review and to provide appropriate recognition for Early Career Researchers who co-review manuscripts.

Version 1:

Reviewer comments:

Reviewer #1

(Remarks to the Author)
The authors have gone to significant lenghts to improve the manuscript and to address each of the points raised in the 1st review. I have no other comments to make other than I think the manuscript will make an impressive addition to the literature and be read and highly cited, especially as it tells us a lot about a drug class currently undergoing clinical trial assessment. I think the journal should publish at once.

Reviewer #2

(Remarks to the Author)
I appreciate that the authors consolidated the difference in ATRi sensitivity among the breast cancer lines they selected, extending their analysis to clonogenic and cell viability assays.

However, I remain largely unconvinced – in fact even less convinced than from the originally submitted manuscript – of the functional link between origin firing and ATRi sensitivity. In a nutshell, key experiments were not performed (with questionable arguments) and those that were performed led to rather controversial data, which do not provide significant support to the central conclusion of this manuscript, but rather challenge it further.

Besides a few minor points (that are omitted here), these are the main concerns on the revised manuscript:

Point 2 (of my original comments): the explanation of the increased gH2AX in sensitive cell lines as "pan-nuclear" signal uncoupled from DNA damage is purely speculative and not substantiated by any data. Moreover, now that careful statistical assessment has been done on micronuclei levels, the data clearly reveal upon ATRi a significant increase in the sensitive lines, which is not significant for the resistant ones. Hence, even though the authors chose a vaguer formulation in their text ("…does not robustly correlate with exacerbated…"), the conclusion that sensitivity is NOT related to increased CIN and genomic instability is essentially challenged by their own new data.

Point 3: MCM2 phosphorylation is well known to reflect origin firing (as opposed to licensing). Hence it is not really clear what the authors are proposing, when suggesting that the sensitive lines are "primed" to achieve higher rates of origin firing only upon ATR inhbition. In essence, along with the other problematic points, these data contribute to challenge the link between sensitivity, P-MCM2 and excessive origin firing. Providing a possible, yet unsupported explanation for this discrepancy – as the authors do in their rebuttal – does not really help substantiating the main conclusion of the study.

Points 4/5: despite possible off-target effects – which are in fact also a possible problem for CDC7i – roscovitine has been previously used in multiple publications that initially showed deregulated origin firing by ATR/CHK1 inhibition or inactivation (see for example PMID: 20805465 or PMID: 21173116). Hence, it is not understandable why the authors refrained from performing this simple and essential control experiment that may (or may not) have backed up their conclusions from CDC7i treatments. Moreover, their new siCDC45 data are puzzling and worrying. Such an effective depletion of an essential initiation factor is expected to affect origin firing significantly (something that the authors did not directly assess). The lack of any effect of such a perturbation on the sensitivity of their lines (Rebuttal Fig. 6) is definitively at odd with the key conclusion of this study. In this context, arguing without even testing it directly that "it is probably very technically challenging to reduce origin firing by manipulating relevant protein levels in cells" is not really useful to resolve such a crucial discrepancy and to increase confidence in the manuscript conclusions.

Inspired by the comment of a different reviewer, the authors performed the opposite experiment (CDC45 OE), but the results they obtained (Fig. 3i-l) are unconvincing. Although the statistics seems to reveal significant differences between the displayed curves (which is surprising per se), the effects are once again marginal, and by no means sufficient to make a resistant cell line even close to the response observed in sensitive lines.

Similarly, extending the use of CDC7i to the colorectal cell lines (Fig. 4d-i) generally showed marginal effects on sensitivity (in some cases even discrepant results, e.g. Fig. 4F). Once again, these marginal effects of CDC7i also in this context can hardly explain in terms of origin firing the differential sensitivity observed among colorectal cancer cell lines.

Overall, even considering all new data added in revision, the entire dataset in the manuscript at best suggests that origin firing has a minor contribution to ATRi sensitivity. However, it does not seem that any of the biomarkers or functional readouts explored by the authors for origin firing could effectively be used to predict ATRi response in cancer cells, which is the key conclusion proposed in the manuscript. Hence, the authors should be encouraged to drastically tone down their conclusions, while submitting a selection of their results to a more specialistic journal.

Reviewer #3

(Remarks to the Author)
The authors sufficiently addressed my comments and I believe the manusscript can now be published.

Reviewer #4

(Remarks to the Author)
I co-reviewed this manuscript with one of the reviewers who provided the listed reports. This is part of the Nature Communications initiative to facilitate training in peer review and to provide appropriate recognition for Early Career Researchers who co-review manuscripts.

Reviewer #5

(Remarks to the Author)
I co-reviewed this manuscript with one of the reviewers who provided the listed reports. This is part of the Nature Communications initiative to facilitate training in peer review and to provide appropriate recognition for Early Career Researchers who co-review manuscripts.

Version 2:

Reviewer comments:

Reviewer #2

(Remarks to the Author)
I appreciate that the authors agreed to proceed with the requested experiments with Roscovitine and removed from the manuscript the confusing data obtained with CDC45 overexpression. I agree that the manuscript is now consolidated on the relevance of origin firing in breast cancer cell lines, although the effects on sensitivity displayed throughout the manuscript are often marginal and would rather suggest a "contribution" of origin firing, rather than a "casual" link with ATRi sensitivity (as currently implicated by their abstract).
I remain unconvinced that any of the markers explored by the authors to monitor origin firing potential in different lines could be used in clinical or preclinical settings to efficiently predict ATRi sensitivity. Most importantly, the data included in the paper to extend their key claim to other cancer types (besides breast cancer) remain very limited. Overall, I remain of the opinion that this manuscript would be appropriate for a more specialistic journal; however, should the editor be convinced to proceed with publication (also in light of the support of the other reviewers), I would recommend to check carefully the key statements of the authors on the generalisation of their statements for cancer, to avoid generating confusion within the field.

Reviewer #4

(Remarks to the Author)
I co-reviewed this manuscript with one of the reviewers who provided the listed reports. This is part of the Nature Communications initiative to facilitate training in peer review and to provide appropriate recognition for Early Career Researchers who co-review manuscripts.

Reviewer #5

(Remarks to the Author)
I co-reviewed this manuscript with one of the reviewers who provided the listed reports. This is part of the Nature Communications initiative to facilitate training in peer review and to provide appropriate recognition for Early Career Researchers who co-review manuscripts.

Open Access This Peer Review File is licensed under a Creative Commons Attribution 4.0 International License, which

permits use, sharing, adaptation, distribution and reproduction in any medium or format, as long as you give appropriate credit to the original author(s) and the source, provide a link to the Creative Commons license, and indicate if changes were made.
In cases where reviewers are anonymous, credit should be given to 'Anonymous Referee' and the source.
The images or other third party material in this Peer Review File are included in the article's Creative Commons license, unless indicated otherwise in a credit line to the material. If material is not included in the article's Creative Commons license and your intended use is not permitted by statutory regulation or exceeds the permitted use, you will need to obtain permission directly from the copyright holder.
To view a copy of this license, visit https://creativecommons.org/licenses/by/4.0/

Lumeau et al, Rebuttal for "High levels of DNA replication initiation factors indicate ATRi sensitivity via excessive origin firing"

We thank all reviewers for their careful reading of our initial manuscript and their expert opinions. We are very happy that overall, the importance of our study was highlighted by all reviewers. We have now addressed all the comments in full (see below) and we feel that our manuscript is strengthened by these revisions.

Summary of major changes to the manuscript and figures:

1. Extended analysis of ATRi-induced pan-nuclear γH2AX and CDC7i rescue of sensitivity to ATRi across all breast cancer cell lines.

2. Extended our findings that origin firing is a key determinant of ATRi sensitivity to a second cancer type (colorectal cancer) using functional experiments. We performed ATRi sensitivity and CDC7i rescue experiments in a panel of colorectal cancer cell lines corroborating our initial predictions from transcriptomics analyses.

3. Performed the reverse experiment of *increasing* sensitivity of ATRi resistant breast cancer cells by upregulating origin firing capacity, demonstrating further that origin firing is a key regulator of ATRi response.

4. Extended transcriptomic signature analyses including the addition of ROC analyses as a step further to understanding the potential clinical utility of our new mechanistic understanding of ATRi sensitivity.

5. Based on our additional experiments and further demonstration of the relationship between origin firing capacity and ATRi sensitivity we have changed title of the manuscript to: **"Replication origin firing capacity indicates ATR inhibitor sensitivity"**.

Reviewer #1 (Remarks to the Author):

ATRi have already been shown to compromise cell cycle checkpoints and hasten premature mitosis, impair replication fork stability and derepress the firing of latent replication origins. This biology has led to the idea that ATR inhibitors have potential in treating cancers with certain types of replication fork stress. However, measuring RFS in cancers is complex and therefore most existing ATRi trials either use genomic surrogates of RFS to select patients for treatment or, sadly, have patient stratification criteria based on histology or resistance to other agents, presuming that these features are also surrogates of RFS. The result of this is that despite nearly a decade of clinical development, not one ATRi has yet been approved for the treatment of cancer. Here Lumeau et al reveal that a subset of breast tumor cell lines and AML patient samples have higher expression of DNA replication initiation factors and increased origin firing upon ATRi exposure and that ATRi sensitivity is causally associated with origin firing rates and decreased by Cdc7 inhibition. The authors argue that this information could lead to the development of multimodal clinical biomarkers for ATRi treatment.

We thank the reviewer for highlighting the important lack of mechanistic information regarding clinically translatable biomarkers for ATRi sensitivity.

Major comments:

1. Fig 1 J – is this analysis consistent with the conclusion that RS-CAN patterns predict ATRi sensitivity ? Cluster 3 has a very small n with a large variance in IC50 and is ns when compared to cluster 2. I'm not sure how valid this conclusion is based on this analysis.

We attempted to identify additional cell line data with matched genomic sequencing and ATRi sensitivity to improve this analysis but were not able to. Therefore, we decided at this point to remove this aspect from the study and continue this work separately when additional data can be acquired.

2. Figure 2 is interesting but it is challenging to reject hypotheses when a sample size (i.e. number of cell lines analyzed) is very small and also when these have so many differences between them, including ER status. I think authors have to acknowledge that whilst the data does not support the idea that the ATRi induced RFS associated chromosomal instability correlates with ATRi sensitivity, they also have little power to reject these hypotheses either.

We agree that due to many confounding variables it is not possible to formally rule out a link between higher CIN and ATRi sensitivity generally. We have therefore reworded the manuscript to reflect this uncertainty, and also reworded the results heading for this section:

"ATRi sensitivity does not robustly correlate with exacerbated increases in RS or associated chromosomal instability in a panel of breast cancer cell lines."

"…….Baseline rates of CIN were not significantly different between the sensitive and resistant lines (Figure 1l-o, Extended Figure 1o). Furthermore, although all cell lines displayed increased CIN after ATRi treatment, there were no consistent differences in the rates nor type of CIN induced by 72h ATRi treatment between sensitive and resistant cell lines (Extended Figure 1o-q). Absolute rates of micronuclei were higher after ATRi in sensitive cells compared to resistant cells however this was not statistically significant (Figure 1n,o), and when accounting for the basal micronuclei rates the relative increases were similar between sensitive and resistant cell lines (Figure 1p). Therefore, neither high baseline rates of CIN nor intolerable levels of ATRi-induced RS or CIN could readily explain the differential ATRi sensitivity of this panel of breast cancer cell lines."

3. Figure 3 L, M – these are very modest differences in ATRi sensitivity shown, even by comparison to what others have shown the antagonism between Cdc7 and ATRi to be (see below).

Although we agree the effect is modest, we note that the result is statistically significant and reproducible. To provide more confidence in this finding, we have now performed new supplemental cell viability assays for all six breast cancer cell lines (using CellTiterGlo) and showed similar results (see revised manuscript - **Extended Figure 3c-d,** also shown below). We also measured an additional read-out of CDC7i rescue of ATRi toxicity by quantifying pan-nuclear γH2AX staining. This analysis revealed a large reduction of ATRi sensitivity in HCC1569 and HCC1806 cells upon CDC7i treatment (revised manuscript - **Figure 3d**, **Extended Figure 3a,** also shown below). BT549 was a consistent outlier in many of these and previous experiments. During revision experiments we identified that this cell line is the only p53-proficient ATRi-sensitive breast cancer cell line, and we suspect this may point to an alternative mechanism of ATRi sensitivity, and which we now mention in the Discussion.

Extended Figure 3c-d:

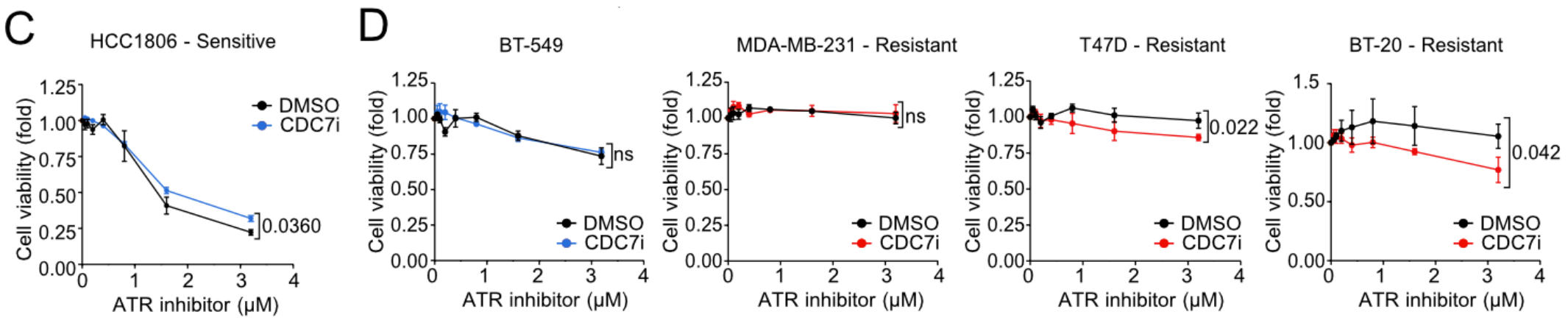

Figure 3d, **Extended Figure 3a:**

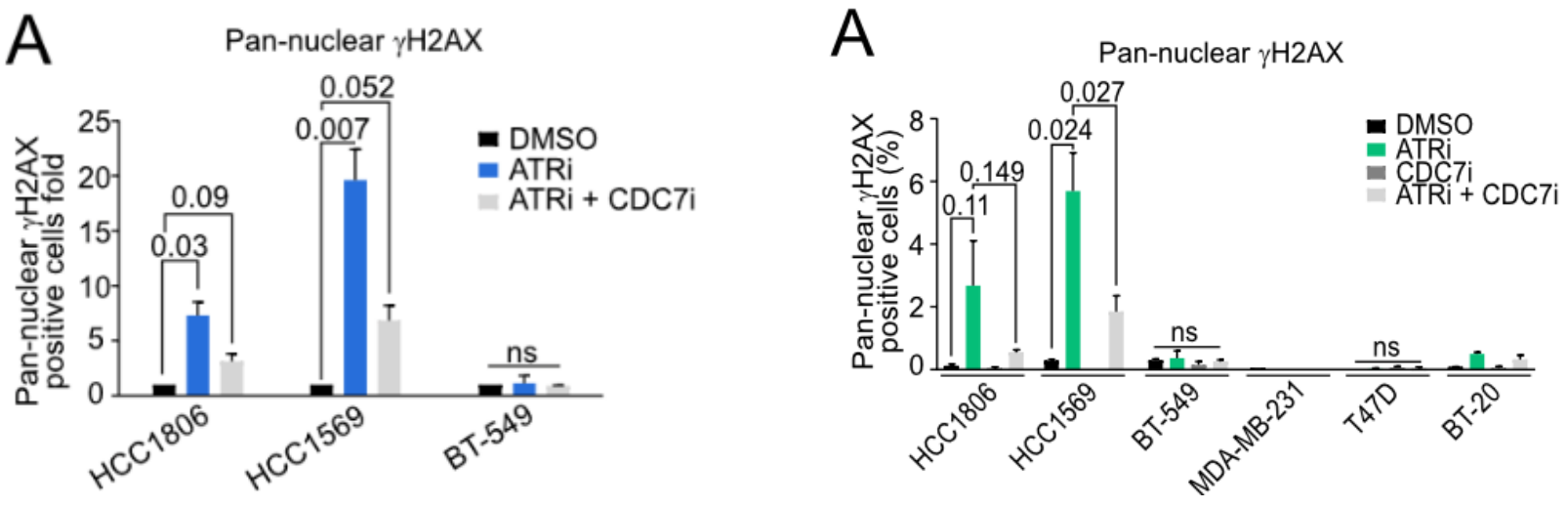

Further strengthening our findings, we also performed a new set of similar experiments in colorectal cancer cell lines in new **Figure 4** (as a response to Reviewer 2), and now additionally show that the decrease of cell viability by ATRi can be rescued by CDC7i in multiple colorectal cancer cell lines (revised manuscript - **Figures 4e-i,** also shown below). We also quantified the rescue of ATRi-induced pan-nuclear γH2AX in ATRi-sensitive RKO cells (revised manuscript – **Figure 4k,** also shown below). Together these new results show that partial inhibition of CDC7 can reduce the phenotypes induced by ATRi in multiple different cell lines from two cancer types by multiple cell biology read-outs, and thus we confirm the contribution of origin firing in ATRi toxicity.

Figures 4e-i:

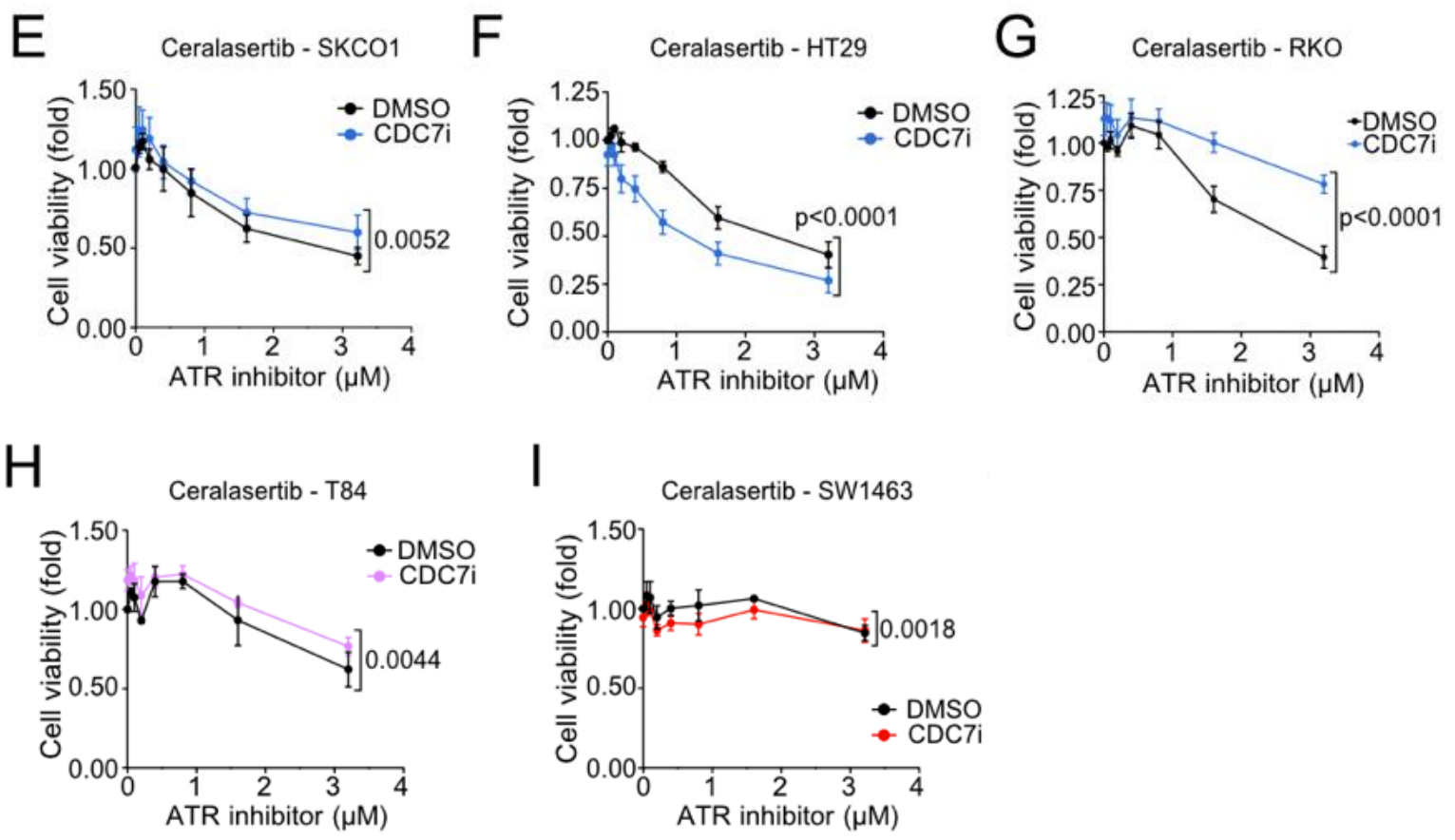

Figure 4k:

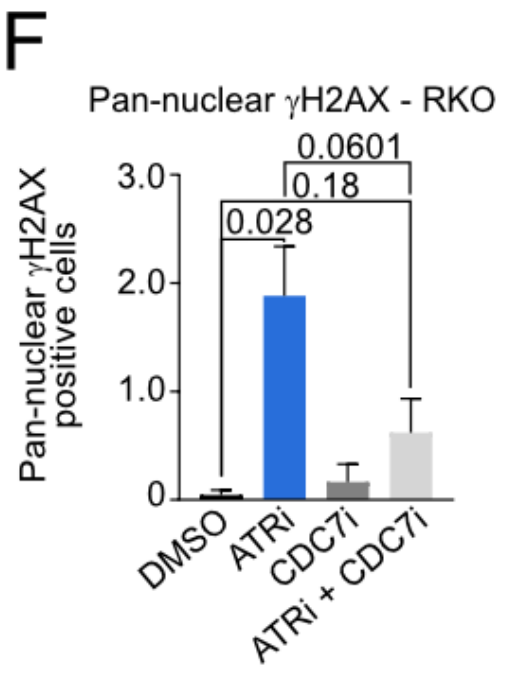

4. If Cdc7 activity is different in ATRi sensitive vs resistant cell lines can authors show that Cdc7i have differing effects in ATRi resistant cell lines?

We previously showed that CDC7i have no, or opposite (decreased cell viability) effects in two ATRi resistant breast cancer cell lines. We have now performed additional viability assays on all three resistant breast cancer cell lines and cell death assays (revised manuscript – **Extended Figures 3d-e**) as well as pan-nuclear γH2AX frequency (revised manuscript – **Extended Figure 3a,** figures also shown below). We also performed cell viability and cell death assays in the most ATRi-resistant colorectal cell line of our panel, HT55 (revised manuscript – **Extended Figure 4j** and shown below). Our new data further support our initial conclusion that ATRi-resistant cell lines display either a mild decrease of cell viability (BT20, T47D), or no effect (MDA-MB-231, HT55) upon co-treatment with CDC7i.

Extended Figures 3d-e:

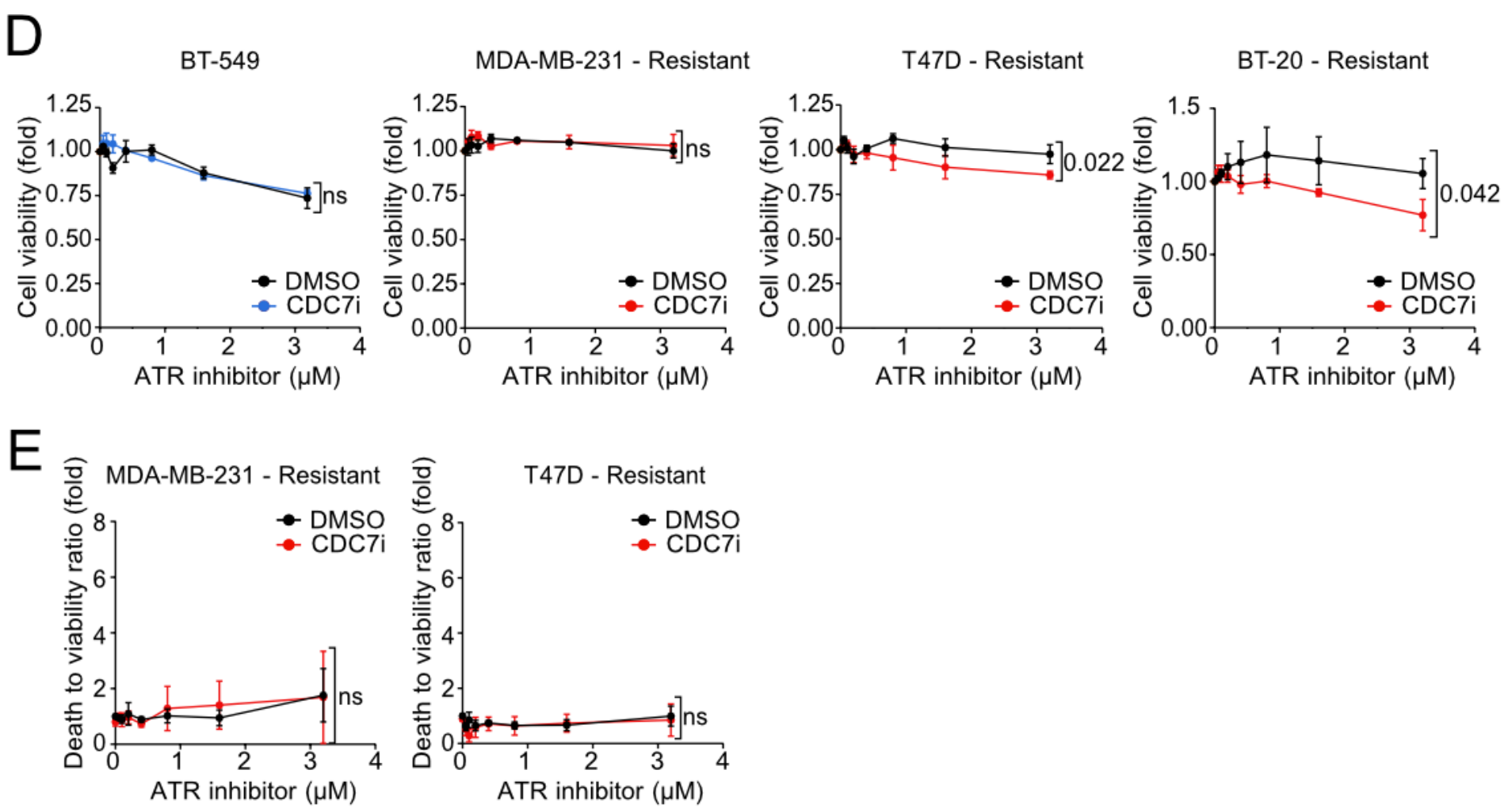

Extended Figure 3a:

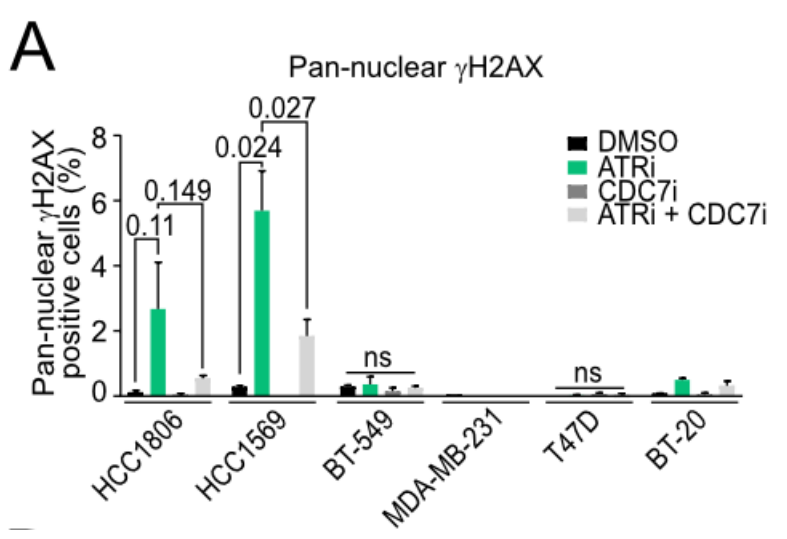

Extended Figure 4j:

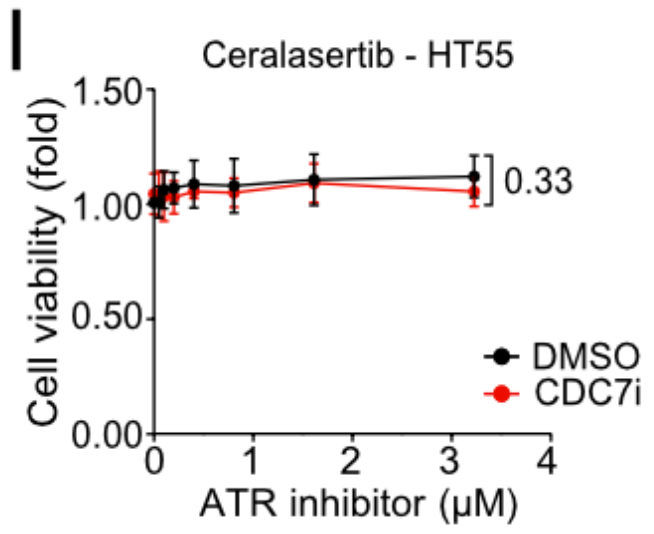

5. I think authors have to acknowledge beyond the extent that they do that the interaction between Cdc7 and ATR inhibition is a well-known and understood phenomenon (e.g. PMID: 37378325 PMID: 34663432 PMID: 32877678 PMID: 31209037 PMID: 29123096 PMID: 12535533).

We agree and thank the reviewer for highlighting these studies. Some of these were referenced in the original manuscript, however we have increased the discussion around these and added the missing references. We would still like to highlight that though previous studies do describe the link between CDC7i and ATRi and that CDC7i can rescue ATR-induced origin firing, our work additionally shows for the first time that cancer cell sensitivity to ATRi can be rescued using CDC7i manipulation of origin firing capacity, and also brings a new insight into the potential translational use of biomarkers of excessive origin firing to predict sensitivity to ATRi by assessing expression and activation of DNA replication initiation factors.

6. In figure 4 authors use GSEA to pull out enriched pathways associated with ATRi sensitivity. Although DNA replication came out as an enriched process, it is important to understand how the genes in this signature actually relate to the biology described in figure 3. For example, which genes in the replication signature are those most highly correlated with ATRi sensitivity?

We agree and now present new tables with the leading-edge genes of each dataset, revealing the genes that contributed the most to the significant positive enrichment of the pathway in sensitive cells (**Supplementary Tables 22-25**). Interestingly only 3 genes in the 38 gene DNA replication initiation signature were communal between all the datasets that we studied: ORC1, CDT1, MCM10. New figures are presented in revised manuscript – **Figure 5 f-i** (also shown below) and discussed in the manuscript to show gene expression signature analysis of the full signature and the 3 communal gene signature of the leading-edge genes for each dataset.

Figure 5 f-i:

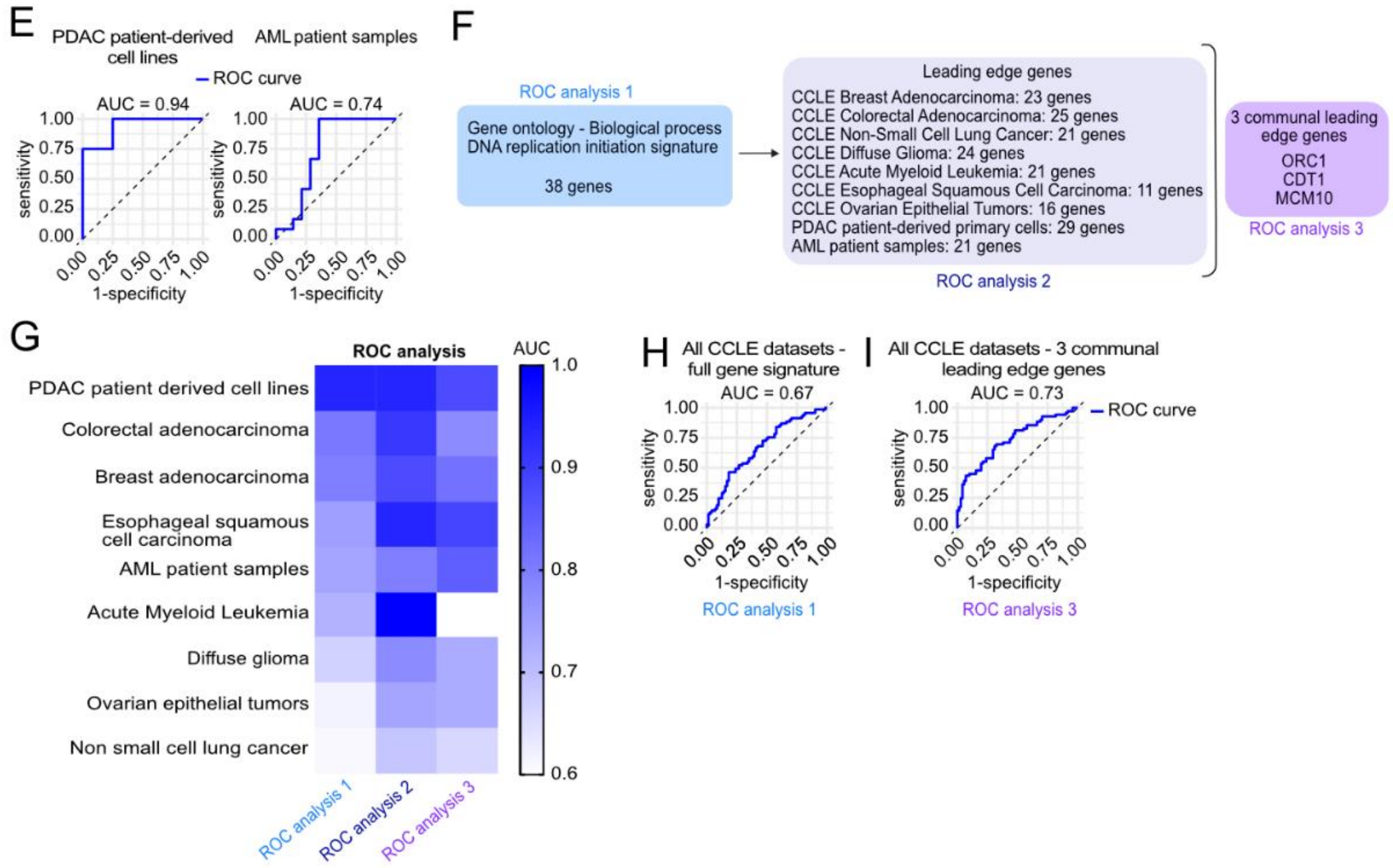

We have now included the following sentence in our results to discuss this:

"As the specificity and sensitivity of the signature to predict ATRi response is variable among different datasets and cancer types, e.g. excellent in PDAC patient-derived cell lines and poor in non-small cell lung cancer, we investigated whether a subset of genes within the DNA replication initiation signature would be sufficient or more efficient at predicting ATRi sensitivity in a pan-cancer manner. For that, we performed and compared the following ROC analyses: analysis 1 using the full GO-term biological process (BP) DNA replication initiation signature of 38 genes; analysis 2 using the leading-edge genes in each dataset; analysis 3 using the leading-edge genes common between all the datasets (Figure 5f). The leading-edge genes per dataset were extracted from the GSEA analysis and corresponded to the genes that contributed to the enrichment of the DNA replication initiation pathway in the sensitive samples versus resistant (Supplementary tables 22-25). We observed between 11 and 29 leading edge genes per dataset. When examining the overlap of these, we found three communal genes consistently in the leading edge across the datasets (hypergeometric test, p-value = 0.0005): ORC1, CDT1, and MCM10 (Figure 5f). The three ROC analyses allowed us to compare the efficiency of each signature to predict sensitivity to ATRi (Figure 5f-g). As expected, we found that ROC analysis 2 using the cancer type-specific leading-edge genes showed higher and better predictive AUC scores across all datasets (Figure 5g, Supplementary Figure 5b). For non-small cell lung cancer cell lines, none of the signatures provided good predictive value despite the enriched gene expression signature in sensitive samples (Figure 4b), suggesting low specificity. Finally, when performing the ROC analyses on all seven CCLE datasets combined, we found that the full signature did not give a good predictive AUC value (0.67, Figure 5h) whereas the three common gene signature gave an improved predictive AUC value (0.73, Figure 5i). Overall, these data suggest that despite the DNA replication initiation gene expression signature having good-to-excellent predictive AUC values for some cancers, it is not currently possible to define a single gene expression signature to accurately predict patient sensitivity to ATRi in a pan-cancer manner, thus cancer type-specific signatures are likely required for optimal biomarker development.

7. Following on from point (6) it might be important to show whether the relationship between the dna replication initiation signature and ATRi sensitivity is stronger than relationships with previously identified biomarkers of ATRi sensitivity.

We agree that it would be interesting to directly compare the performance of our DNA replication initiation gene expression signature as a predictor of sensitivity to ATRi with other previously identified potential biomarkers. However, this has proved quite difficult to do as we could not identify a suitable comparative metric to compare between different datasets and sensitivity markers, however we investigated whether we could gain insights from comparing to ATM mutation, the only biomarker

currently investigated in clinics to predict sensitivity to ATRi monotherapy[1,2]. We extracted ATM mutation information from CCLE and Dreyer *et al*[3] datasets and analysed how efficient ATM mutation status was to predict ATRi sensitive cell lines (the cell lines with the lowest 25% IC50 in each dataset). We measured the percentage of all cell lines per cancer type with ATM mutation, and the proportion of these cell lines that were considered as the most sensitive of the dataset, see **Figure R1a** below. We then measured the percentage of sensitive, intermediate or resistant cell lines with an ATM mutation, or no mutation in breast and colorectal cancer cell lines (see **Figure R1b-c** below).

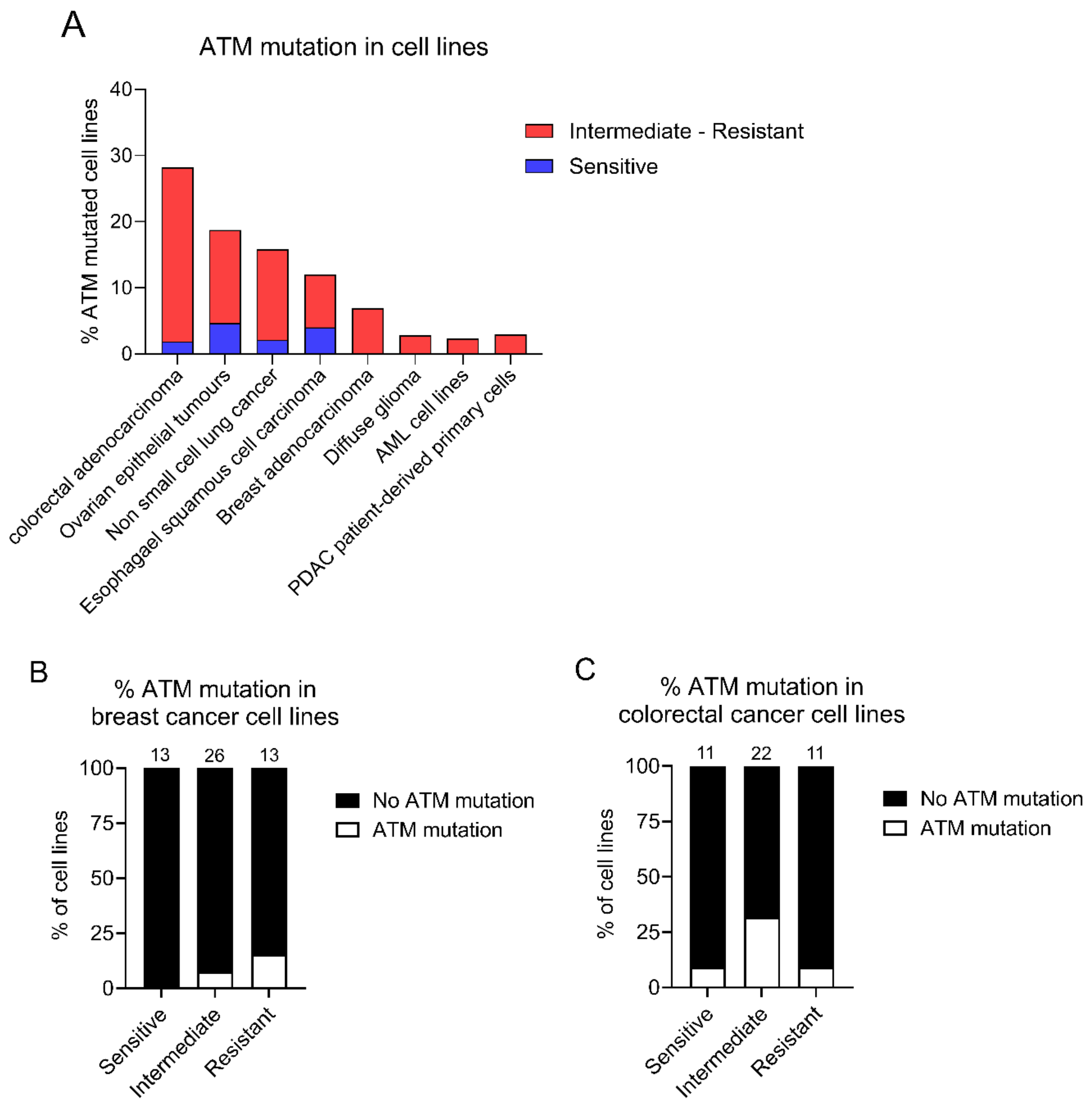

Rebuttal Figure 1. (**A**) Quantification of the percentage of cell lines with an ATM mutation that are considered as sensitive (bottom 25% IC50 of each dataset) or resistant (top 25% IC50) - intermediate. Quantification of the percentage of sensitive, intermediate and resistant cell lines with an ATM mutation or no ATM mutation in (**B**) breast cancer cell lines and (**C**) colorectal cancer cell lines. Figures not presented in the revised manuscript.

We note that this analysis is preliminary and does not take into account the functionality of ATM mutations, nor whether ATM function is impaired through other means. However it is clear that though ATM is mutated in around 2-23% cell lines across all cancer types and datasets, this status does not overlap well with ATRi sensitivity, and there are many ATRi sensitive cell lines that lack ATM mutations that are not predicted by this biomarker. Therefore ATM mutation status alone appears to be neither

sensitive nor specific for ATRi sensitivity. Moreover, a low number (or none) of sensitive cell lines had ATM mutation in breast and colorectal cancer cell lines.

Although our gene expression signature was not applicable across all cancer types, it gave a very good predictive value in colorectal cancer cell lines, breast cancer cell lines and PDAC patient-derived primary cell lines, where none of the four most sensitive cell lines had an ATM mutation. We note that these comparisons are restricted by the available datasets with genomic mutations, gene expression and IC50 for ATRi, so further large-scale analysis with statistical analysis would be required to properly determine if the DNA replication initiation signature is a better predictor than ATM mutation, however based on our new ROC analyses we feel that our predictor is more specific and sensitive than ATM mutation alone.

8. ATRi other than ceralasertib have been described and have data in large scale tumor cell line screens. For the sake of rigor, can the authors show similar relationships exist with ATRi sensitivity in these other datasets?

To address this valid point, we performed new analyses using the publicly available GDSC2 data (drug screen across all breast cancer cell lines, as described in the revised manuscript) of VE821 and VE822, the only other ATR inhibitors available in the GDSC2 dataset. We found that the DNA replication initiation signature is predictive of sensitivity only to Ceralasertib (AZD6738) in breast cancer, and not to VE821 and VE822 as shown in the **Figure R2 a-c** below.

A

	AZD6738		VE821		VE822	
	Sensitive	Resistant	Sensitive	Resistant	Sensitive	Resistant
Total	12	12	13	13	13	13
AZD6738			9	10	6	6
VE821	9	10			6	7
VE822	6	6	6	7		

AZD6738			69%	77%	46%	46%
VE821	75%	83%			46%	54%
VE822	50%	50%	46%	54%		

B

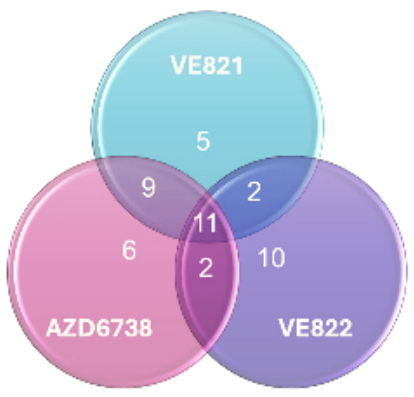

C

GOBP_DNA replication initiation

ATRi	NES	pvalue	Enrichment
VE821	0.92	0.573	No
VE822	0.96	0.534	No
AZD6738	1.74	0.003	**Yes**
Communal	0.40	1	No

Rebuttal Figure 2. (**A**) For each ATRi inhibitor the total number of breast cancer sensitive and resistant cell lines that were identified from the dataset is shown. This number is then compared between inhibitors to report the number of cell lines identified as similarly sensitive/resistant. Percentages are given in the lower table. We found that VE821 sensitive/resistant cell lines are around 80% similar with AZD6738, while VE822 cell lines are only 50% similar with AZD6738. (**B**) Venn diagram representation of the number of breast cancer cell lines (resistant and sensitive combined) in common between the three ATRi inhibitors. (**C**) Table showing the result of the GSEA analysis for each inhibitor, presenting the normalized enrichment score and the p-value for the enrichment of the GO terms BP-DNA replication initiation. This shows that the signature predicts the sensitivity only to AZD6738. Not presented in the revised manuscript.

This appears to be primarily because the three drugs have surprisingly non-overlapping sets of sensitive and resistant cell lines (**Figure R2a,b**). This suggests each inhibitor may have slightly different mechanisms of action, or pharmacological activity despite all converging on ATRi inhibition. Of the other ATRi inhibitors, VE821 has subsequently been shown to have non-specific targets and be less effective than VE822[4,5]. VE822 has a different chemical structure and pharmacological properties to AZD6738 (Cerelasertib)[5] which might explain some of these differences. Of note, only 50% of ATRi-sensitive cell lines are in common between VE822 and AZD6738 (Cerelasertib), meaning that other factors than the activity against ATR may influence sensitivity. Given these differences in activity of the three compounds it is perhaps not surprising that the DNA replication initiation signature loses predictive ability for these other ATR inhibitors, and suggests more broadly that a specific biomarker may be needed for each individual ATRi inhibitor to overcome differences in potency, mechanism, and secondary targets between inhibitors.

9. Ideally, predictive biomarkers need to be binary classifiers (i.e. based on the analysis of the biomarker, does the patient fall into the “sensitive/should receive the drug or resistant/shouldn’t receive the drug classes). Can the authors carry out a more formal ROC analysis or similar to test the sensitivity and specificity performance of a metagene of replication signature?

We thank the reviewer for this suggestion, and we have now performed ROC analysis of the GO-term biological process DNA replication initiation and measured AUC values for each dataset in revised manuscript - **Figure 4c and 5e** and shown below. Please also see our response to your point 6 above. We have also added a new dataset of pancreatic ductal adenocarcinoma patient-derived primary cell lines from Dreyer et al. 2021[3], a study that uses transcriptomics and genomics to investigate new therapeutic opportunities in DDR-proficient pancreatic cancer, and performed similar GSEA and ROC analysis. This analysis revealed a strong enrichment of the signature in the sensitive samples (revised manuscript – **Figure 5a,e** and shown below).

Figure 4c and 5e:

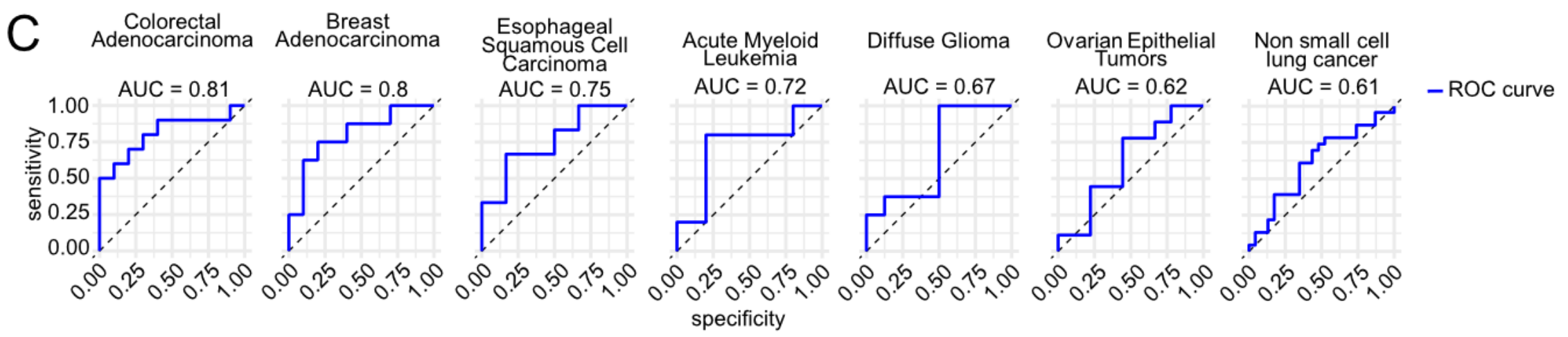

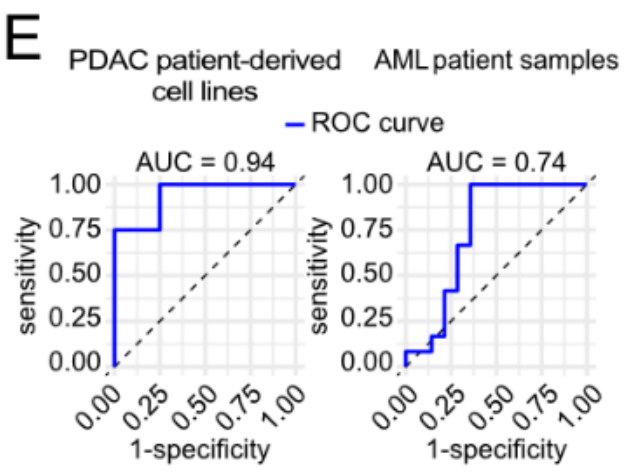

Figure 5a:

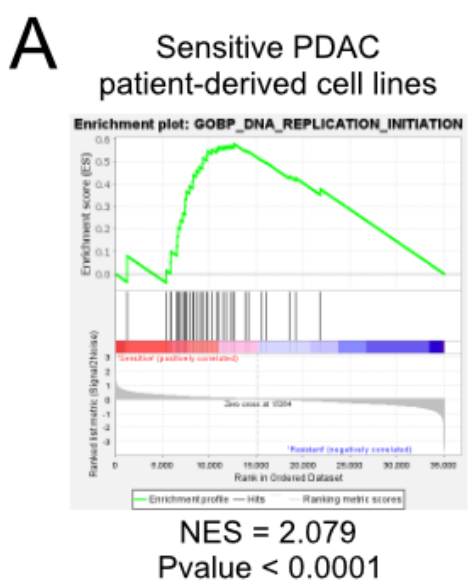

Following up on that, we also performed ROC analysis for each dataset using the leading-edge genes instead of the full 38 gene signature and using the 3 leading edge genes that were in common between all tested datasets (revised manuscript - **Figure 5f-i**). The ROC analysis using these 3 genes increased the AUC of the 7 CCLE datasets together from 0.67 (poor) to 0.73 (fair), as well as increased some individual AUC as for the AML patient data with an increase from 0.74 (fair) to 0.85 (good) (revised manuscript –**Figure 5g**). Although the full signature ROC analysis failed in glioma, NSLC and Ovarian as the AUC score was too poor, using the 3 common leading-edge genes on the AUC score reached above 0.7 in Glioma and Ovarian cancer.

Figure 5f-i

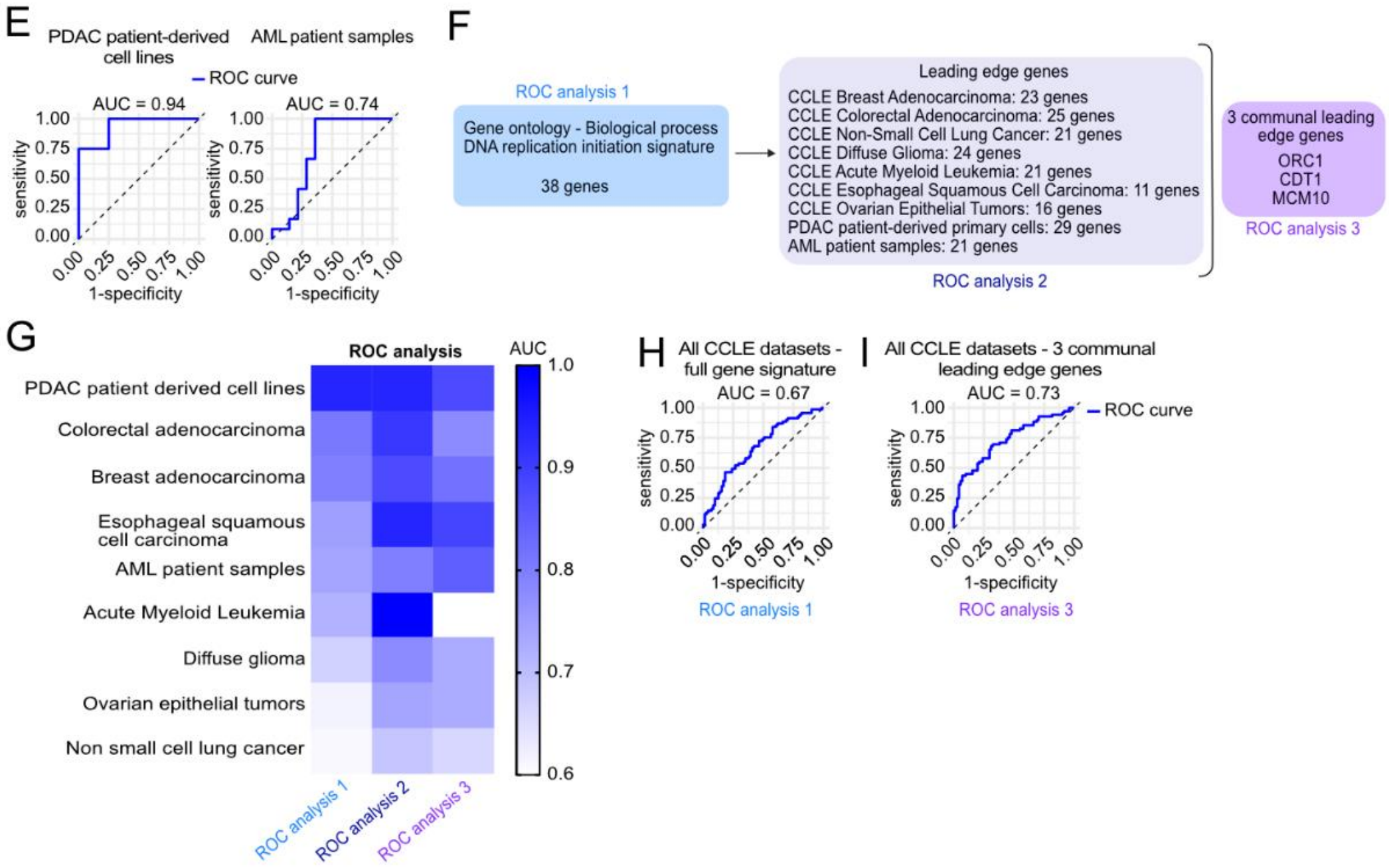

Minor comments:

1. Fig 1. Is it necessary to show that Cyclin E over expression causes a very minor increase in ATRi sensitivity, given this has been known since 2011 (PMID: 21552262)?

We agree this is already known however we included this data to show the impact of CCNE1 overexpression and aphidicolin in our system, as they provide similar difference in ATRi sensitivity range to that present in our cell line panel. We have now moved this panel to revised manuscript – **Extended Figure 1d-e**.

2. Fig 1 A, B ANOVA stats on the entire dose response curve might be more appropriate than single dose t tests.

Thank you for this suggestion, we have now performed two-way ANOVA analysis on all dose response curves and updated the Figures and Figure legends accordingly.

Reviewer #2 (Remarks to the Author):

In this manuscript Lumeau et al. investigate what makes cancer cells sensitive to ATR inhibitors (ATRi). Several of these inhibitors are in clinical trials, but there is currently no reliable biomarker to predict tumor sensitivity to them. Hence, the manuscript tackles a urgent clinical need and provides important clues on an active and important research area.

We thank the reviewer for highlighting the importance of our study.

The authors initially focus on breast cancer cell lines, establishing a generic (and highly expected) link between drug- or oncogene-induced replication stress (RS) and ATRi sensitivity, although the correlation with oncogene expression is not very strong. They also report no clear correlation of ATRi sensitivity with other typical RS markers, such as general levels of pre-existing or ATRi-induced chromosomal rearrangements, fork slowdown or fork stalling, excluding these functional readouts as

possible predictive biomarkers of ATRi responsiveness. However, what they do report based on in-depth genomic investigations on sensitive and resistant breast cancer lines is a strong correlation between ATRi sensitivity and a specific pattern of copy number abnormalities, linked to the propensity of the sensitive lines to unleash replication origin firing. The authors consolidate this evidence by showing that sensitive cell lines have increased levels of initiation factors (CDC45) and/or specific marks of origin firing such as pMCM2, which leads to higher DNA damage and cell death upon ATR inhibition. Furthermore, the genotoxic effects of ATRi in the sensitive lines are partially rescued by counteracting the increased origin firing with a CDC7 inhibitor. In the final part of the manuscript, the authors try to extend the pan-cancer relevance of their findings by analyzing publicly available drug sensitivity, transcriptomic and phosphoproteomic datasets in various tumor types and more specifically in an AML patient cohort, observing a generic correlation between sensitivity to ATRi and increased DNA replication initiation, via transcriptomic and phosphoproteomic signatures. Based on these data, the authors suggest that markers of unleashed origin firing could be explored as general predictive biomarkers of ATRi sensitivity in cancer.

The manuscript is generally well written, and some of the conclusions are well supported and discussed, which may in principle supports publication in Nature Communications after appropriate revisions. While – with few exceptions (see below) – the results in breast cancer cell lines are overall convincing, the work on other cancer types is clearly under-developed, questioning how far the proposed biomarker could be applicable to all cancers. Title, abstract and discussion are currently pushing this "pan-cancer" aspect quite a lot, which should be corrected unless the authors manage to significantly consolidate the second part of their manuscript. A few additional concerns should be addressed, also on the first part of their results in breast cancer cell lines.

We thank the reviewer for their in-depth review and we have now specifically strengthened the pan-cancer aspect as advised (see below).

Major points:

1. Sensitivity throughout the manuscript was solely assessed by cell confluence. Given that cell sensitivity is key to the conclusions of this manuscript, the authors should exploit a different, independent mean to assess cell survival (e.g. clonogenic assays) and (hopefully) confirm all key results in their manuscript. Moreover, it would be very important to distinguish whether the effects on cellular growth are due to cytostatic (cell cycle arrest) or cytotoxic (cell killing) effects.

We thank the reviewer for this suggestion. We have now performed clonogenic assays for three cell lines that we were amenable to single cell cloning (see revised manuscript – **Extended Figures 1h-j**) as well as cell viability assays (CelltiterGlo) for all six cell lines (see revised manuscript – **Extended Figure 1g**) and cell death assays (green cytotox) (see revised manuscript – **Figure 2k-l**). These new data confirm HCC1806, HCC1569 and BT549 as the most sensitive and T47D, MDA-MB-231 and BT20 as the most resistant cell lines of the panel, as shown in the original manuscript. It also recapitulates our original result that among the sensitive cell lines, the cytotoxic effect is higher in HCC1806.

Extended Figure 1g:

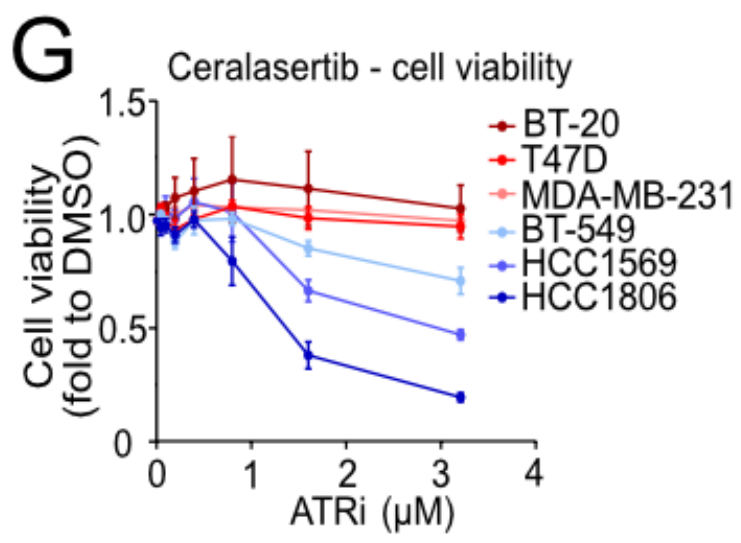

Extended Figures 1h-j:

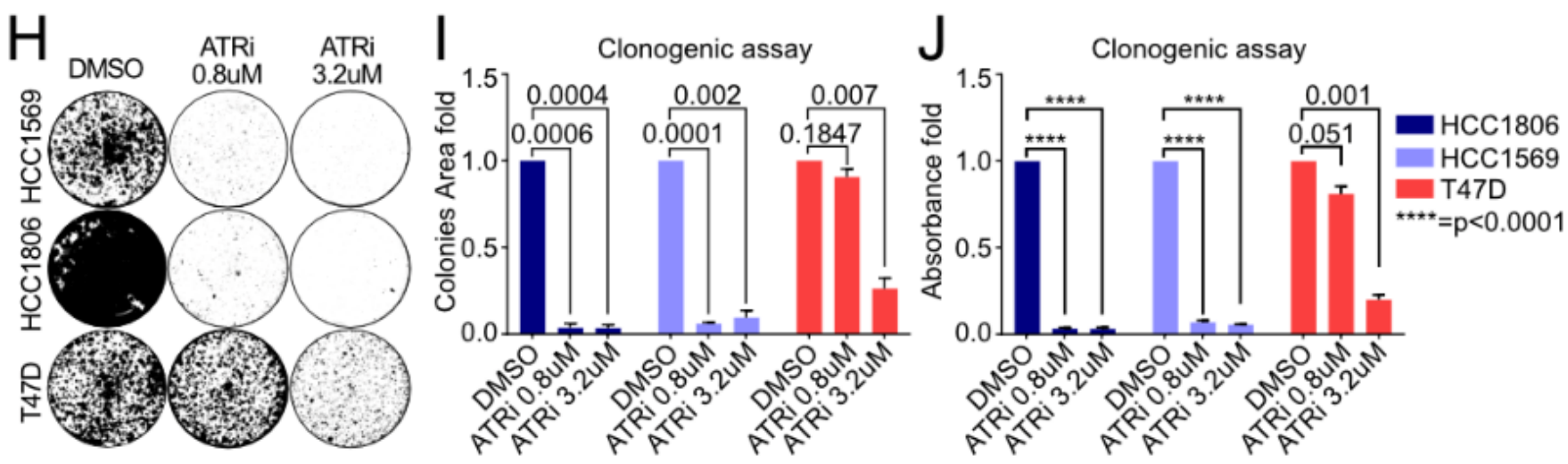

Extended Figure 2k-l

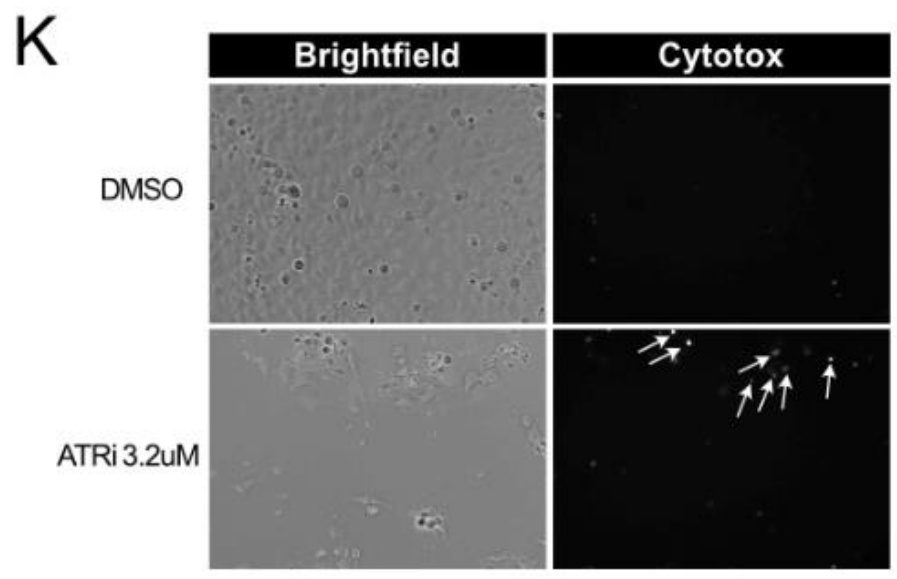

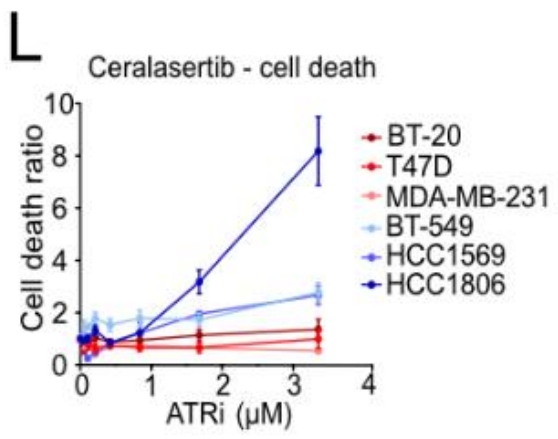

2. In Figure 2F-I and Extended Figure 2B, the authors should further discuss the origin of the increased CIN upon ATRi in the resistant cell lines. In this respect, how do the authors explain that ATRi-sensitive and resistant cell lines present similar levels of CIN upon ATRi, but only the sensitive ones exhibit increased DNA damage signaling upon ATRi (Figure 3E-F)?

We agree that the origin of CIN and the differences of segregation errors and micronuclei rates between sensitive and resistant cell lines should have been more fully discussed in the manuscript. We have updated the figures in this section to more clearly compare CIN between the cell lines. We have also included more information about how replication stress can generate chromosome segregation errors and MN:

"Replication stress is a driver of chromosomal instability; incomplete DNA replication, mitotic DNA breakage of fragile sites, and a multitude of other mechanisms can all drive the generation of chromosome segregation errors and micronuclei[6-10]*."*

In terms of the difference in increased DNA damage signalling, we actually think that the increased H2AX-Ser140 signal observed by mass spec (and now additionally by western blotting) is a result of the pan-nuclear γH2AX signal that occurs only in sensitive cells upon ATRi, rather than increased DNA damage signalling *per se*.

Moreover, in Figure 2I the authors claim that no significant differences in CIN levels by micronuclei formation can be observed between sensitive and resistant cell lines. On the contrary ATRi induces up to 40% of cells with micronuclei in sensitive cell lines while the increase in the resistant only up to 20%. Statistics are not shown comparing those 2 conditions, but a trend is clearly observable. These statements seem overall not well supported and may need to be far less dogmatic, providing statistical significance data for each specific comparison. More in general, the authors should systematically calculate and display absolute p-values (as opposed to * or ns) to support key statements throughout their manuscript.

We agree that the absolute MN rate in sensitive cells treated with ATRi is higher than in the resistant cells, which is now also made clearer in **Figure 1o**. We have now included a direct statistical comparison of absolute and relative increases in MN after ATRi treatment in **Figure 1p**.

Figure 1o-p:

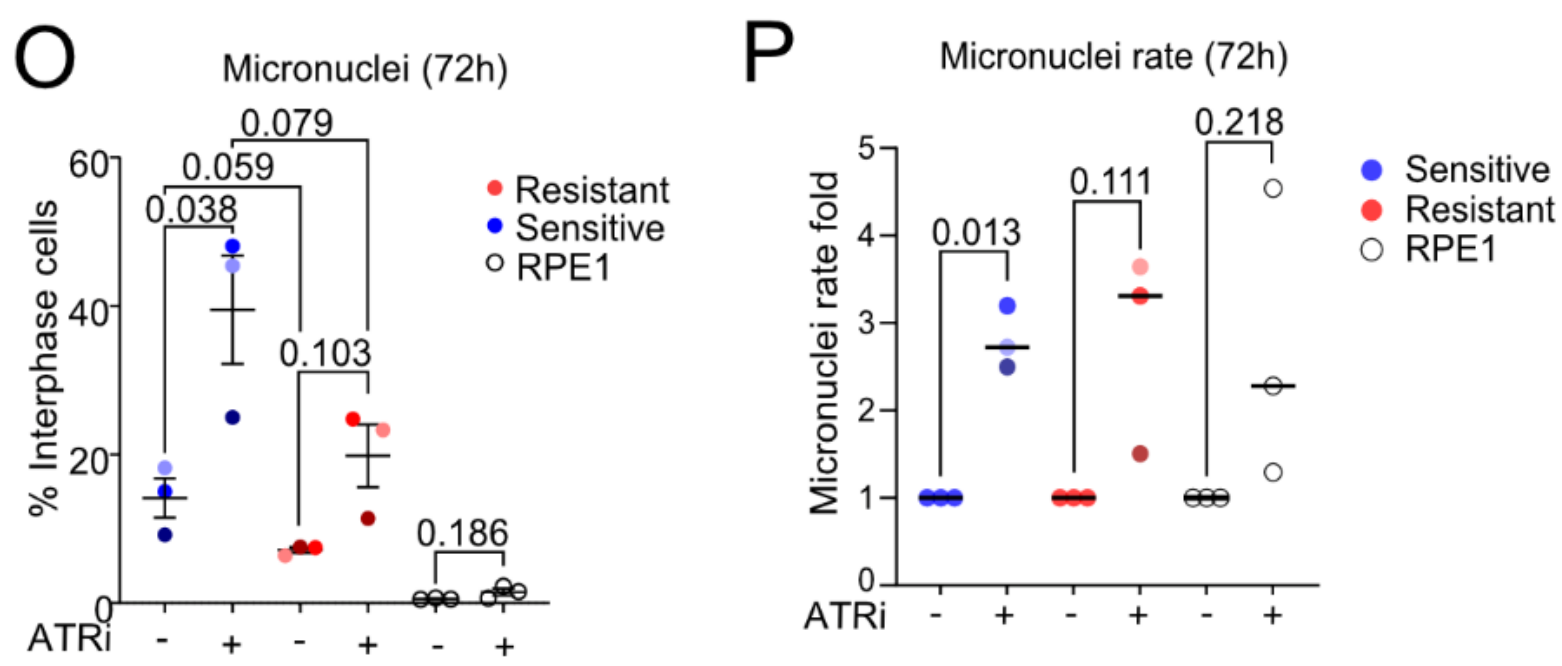

We also note that relative increases in MN upon ATRi are not different between sensitive and resistant cell lines. We also provide a more detailed discussion of these data and tone down our conclusion that RS/CIN does not explain ATRi sensitivity including rewording the section heading:

"ATRi sensitivity does not robustly correlate with exacerbated increases in RS or associated chromosomal instability in a panel of breast cancer cell lines."

"…….Baseline rates of CIN were not significantly different between the sensitive and resistant lines (Figure 1l-o, Extended Figure 1o). Furthermore, although all cell lines displayed increased CIN after ATRi treatment, there were no consistent differences in the rates nor type of CIN induced by 72h ATRi treatment between sensitive and resistant cell lines (Extended Figure 1o-q). Absolute rates of micronuclei were higher after ATRi in sensitive cells compared to resistant cells however this was not statistically significant (Figure 1n,o), and when accounting for the basal micronuclei rates the relative increases were similar between sensitive and resistant cell lines (Figure 1p). Therefore, neither high baseline rates of CIN nor intolerable levels of ATRi-induced RS or CIN could readily explain the differential ATRi sensitivity of this panel of breast cancer cell lines."

We agree regarding the reporting of statistical tests and have now included specific p values throughout the figures and statistical analysis test information in the figure legends.

3. In Figure 3D, the authors show higher levels of MCM2 phosphorylation (indicative of higher origin activation) in sensitive compared to resistant breast cancer cell lines in basal conditions. On the contrary, Figure 3G shows no differences in origin firing levels between sensitive and resistant lines in the same conditions. How do the authors explain this observation, which seems clearly at odd with one of the key claims of the manuscript?

We thank the reviewer and agree with this comment. There are two potential interpretations of these apparently conflicting findings. First, it could be that the sensitive cell lines are 'primed' for higher

origin firing upon ATRi treatment, rather than exhibiting higher rates of origin firing under basal conditions. Alternatively, we may simple be unable to detect increased origin firing rates at basal level, due the technical difficulties with identifying origin firing events. We now explain this in the results:

*"No difference in origin density was detected between sensitive and resistant cells in untreated conditions, however it is possible that the limit of detection for this assay precludes this observation (**Figure 2g**)."*

4. The effect of the CDC7 inhibitor on cell survival upon ATRi is at best minor (Figure 3L-M). By no means it is sufficient to rescue cell survival to the level observed in resistant lines (see ED Figure 3). The authors should further support this key claim by

a) preventing origin firing by an alternative, independent inhibitor (e.g. Roscovitine? Please note that CDK2 seemed to have even higher enrichment than Cdc7 in their initial screen);

b) displaying that – besides the mild effects on survival – also other key readouts of ATRi genotoxicity (DDR markers or DNA damage itself) are suppressed by limiting origin firing. In fact, in Figure 3O, the authors claim a change in % of sensitive cells with pan-nuclear gH2AX when treated with ATRi, but statistical significance says the contrary. Overall, providing additional lines of support to this central claim of the manuscript seems essential to grant publication of the study.

We agree that the rescue provided by partial CDC7 inhibition is minor. However, we did not expect CDC7i to fully rescue sensitivity. Firstly, we carefully titrated CDC7i to reduce origin firing without impacting cell viability as in a previous study[6]. Reducing origin firing capacity even further may be possible but it is difficult to obtain the precise balance without negatively impacting cell viability. Secondly, ATR's role in the cell is multifaceted and we focused here only on ameliorating the contribution of exacerbated origin firing increases in ATRi sensitivity, meaning that other drivers of sensitivity could still operate in sensitive cells. Our new data using a panel of colorectal cancer cell lines, and the additional experiments outlined above in response to Reviewer 1 and summarised at the start of this rebuttal we think show that CDC7i-induced rescue is very reproducible across sensitive cell lines from two different cancer types, and using multiple cell viability and γH2AX pan-nuclear staining assays.

a) We agree testing limiting origin firing by an independent means is an excellent suggestion. We decided not to use CDK2i as most available inhibitors have off-target effects, targeting several CDK kinases in addition to the one suggested by the reviewer. We instead performed partial protein depletion of CDC45 and MCM2 using siRNA, in order to reduce the pool of origin firing factors and limit origin firing, followed by cell viability assays in response to ATRi. Unfortunately, we could not obtain sufficient depletion of MCM2 in HCC1569 or BT549 cells, and could only slightly decrease protein expression in HCC1806, as presented in **Rebuttal Figure 4** below. We decided therefore to focus on depleting CDC45 for further assays.

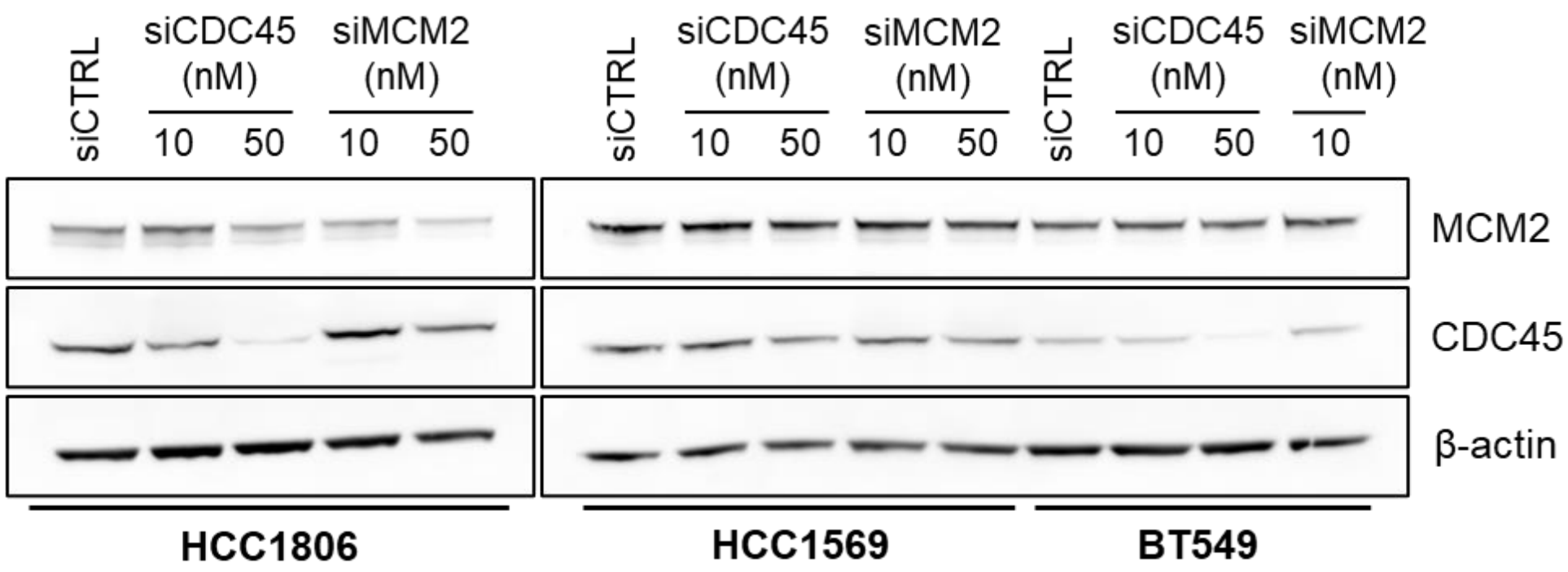

Rebuttal Figure 4. Western Blot of MCM2 and CDC45 protein expression in HCC1806, HCC1569 and BT549 transfected with siCTRL or pools of siMCM2 or siCDC45. Not presented in the revised manuscript.

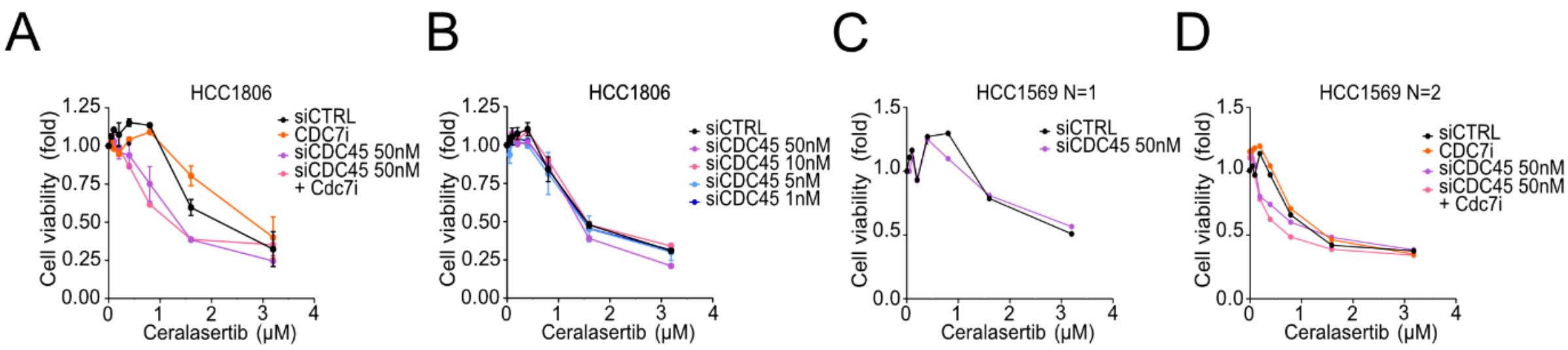

Rebuttal Figure 5. Cell viability assays in response to ATR inhibitors of (**A**,**B**) HCC1806 and (**C**,**D**) HCC1569 transfected of siCTRL or a pool of siCDC45. CellTiterGlo assay was performed 72h after ATRi treatments (6 days post-transfection). Not presented in the revised manuscript.

Interestingly, depleting CDC45 to very low levels (50nM siCDC45) did not cause obvious adverse effects (see HCC1806 cells western blot for CDC45 and cell proliferation data in Rebuttal figure R5 and R6 below).

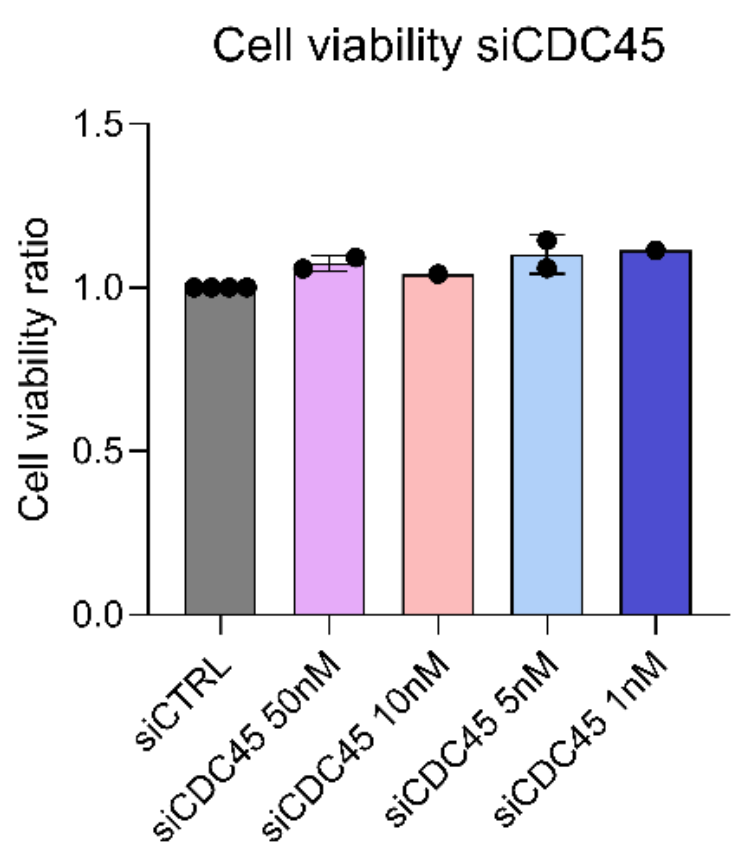

Rebuttal Figure 6. Cell viability (CellTiterGlo assay) of HCC1806 treated with siCTRL or siCDC45 50nM, 10nM, 5nM or 1nM, 6 days post-transfection (24h transfection).

This suggested to us it is probably very technically challenging to reduce origin firing by manipulating relevant protein levels in cells sufficiently to impact origin firing, without depleting protein levels completely, which would presumably be incompatible with passage through S-phase. Moreover, in both HCC1806 and HCC1569, 50nM siCDC45 *decreases* cell viability after ATRi treatment, suggesting that this extreme reduction in levels of CDC45 rather impedes origin firing to too great an extent when ATRi is present. We repeated the experiment with lower doses of siCDC45 (10nM, 5nM and 1nM) in HCC1806. Despite a partial depletion in the 10nM dose (see **Figure R5** above), no change in cell viability

was observed. Overall, we conclude that controlling for the right protein level of CDC45 to be able to reduce origin firing in a similar way as the CDC7i and so rescue partially the effect of ATRi on cell survival is not feasible.

However, we were able to do the opposite experiment of elevating CDC45 levels and sensitising resistant cell lines (as suggested by Reviewer 3) which is now included in the revised manuscript (**Figure 3i-l**):

"We next reasoned that, if resistant cell lines are able to escape ATRi-mediated cell death by limiting excessive origin firing, then inducing higher origin firing rates in ATRi resistant cells might reverse ATRi resistance. To investigate this, and given that CDC45 is already highly expressed in sensitive cell lines (Figure 2b, Extended Figure 2a-d), we transiently overexpressed CDC45 (as done previously to increase origin firing[11]) in the three resistant breast cancer cell lines MDA-MB-231, T47D and BT-20. Western blot assays confirmed the increase of expression of CDC45 protein levels in all three cell lines (Figure 3i). Interestingly, CDC45 overexpression led to a mild but significant sensitization to ATRi in all three resistant cell lines (Figure 3j-l), while having no impact on cell viability in the absence of ATRi (Extended Figure 3f). This demonstrates that origin firing must be tightly regulated in cancer cell lines to resist ATRi, since decreasing origin firing (with CDC7i) or increased origin firing (with overexpression of CDC45) could both reduce the viability of cell lines initially resistant to ATRi, particularly exemplified by T47D which was sensitive to both manipulations (Extended Figure 3b,d,e Figure 3i,k). Altogether our results provide evidence that origin firing capacity is an important factor in determining sensitivity to ATRi and that manipulating origin firing can modulate the ATRi response in both directions."

Figure 3i-l:

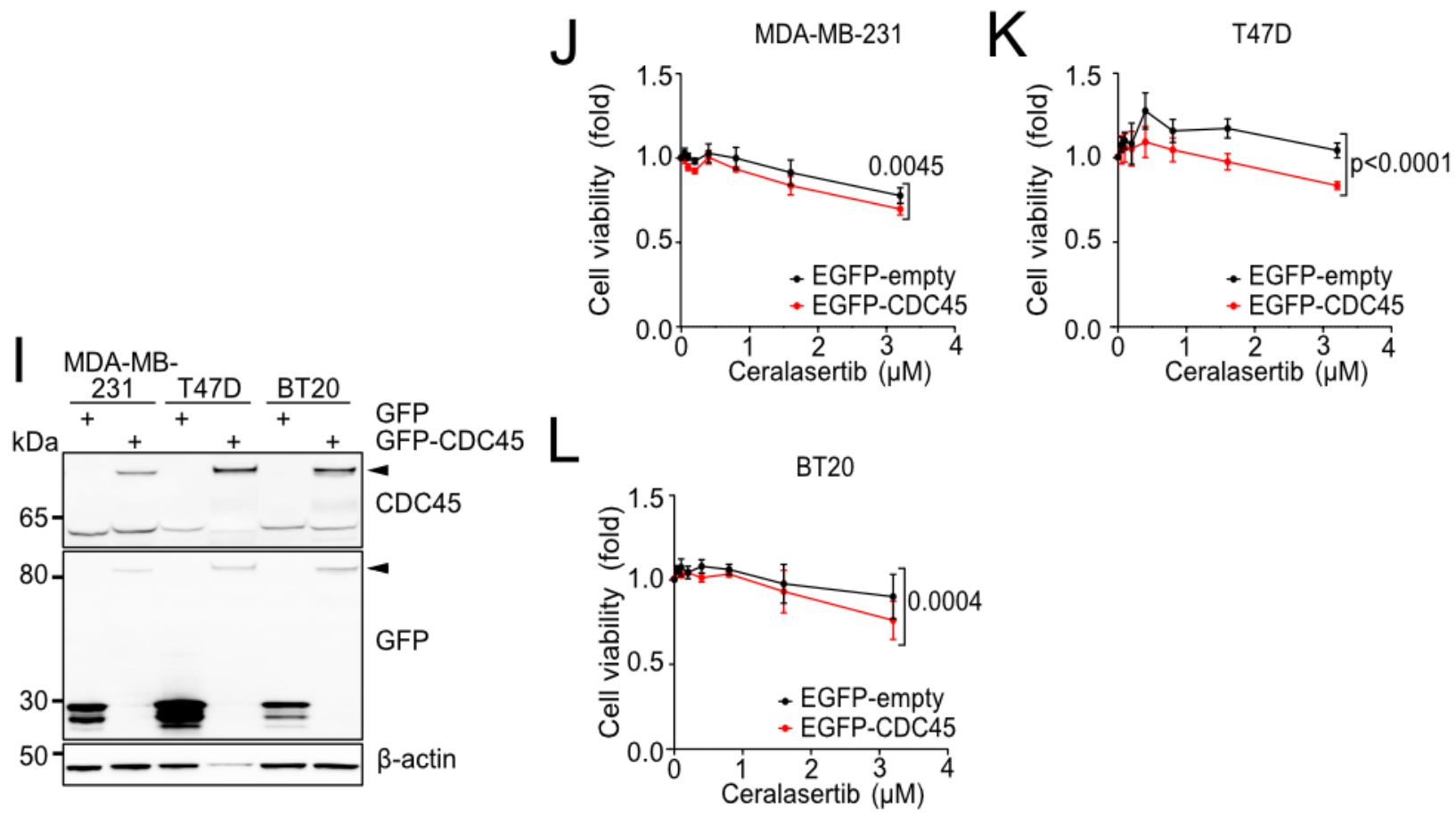

B) We agree and have now performed more repeats of the γH2AX pan-nuclear quantification in all six breast cancer cell lines. For increased clarity we have plotted cell lines individually instead of grouping as sensitive vs resistant and performed statistical analysis for each cell line individually (revised manuscript – **Figure 2i-j**, **Extended Figure 2k**). This confirms the increase of γH2AX pan-nuclear-positive cells in response to 24h ATRi in sensitive HCC1806 and HCC1569 cells and not in resistant/intermediate BT549, T47D, MDA-MB-231 and BT20 cells. We also performed the same assays in the colorectal cancer cell line RKO and show similar increases in pan-nuclear γH2AX (revised manuscript **Figure 4k**). In these three cell lines (HCC1806, HCC1569, RKO), the effect of ATRi was significantly rescued by CDC7i (revised manuscript - **Figure 3d**, **Extended Figure 3a, Figure 4k**). Finally, we performed western blot assays and confirmed the increase of γH2AX in HCC1806, HCC1569 (revised manuscript **Figure 2f, Extended Figure 2g**) and in several colorectal cancer cell lines (revised manuscript - **Extended Figure 4d**).

Figure 2i-j:

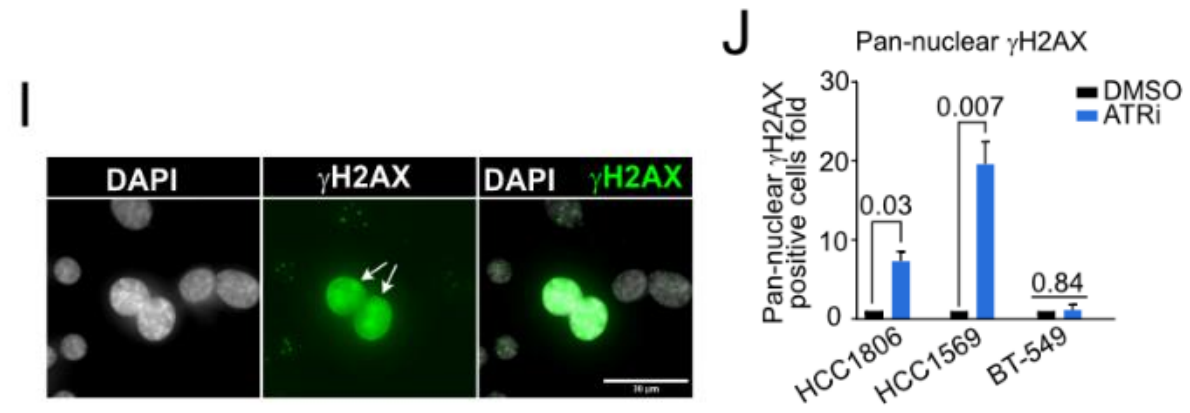

Extended Figure 2k:

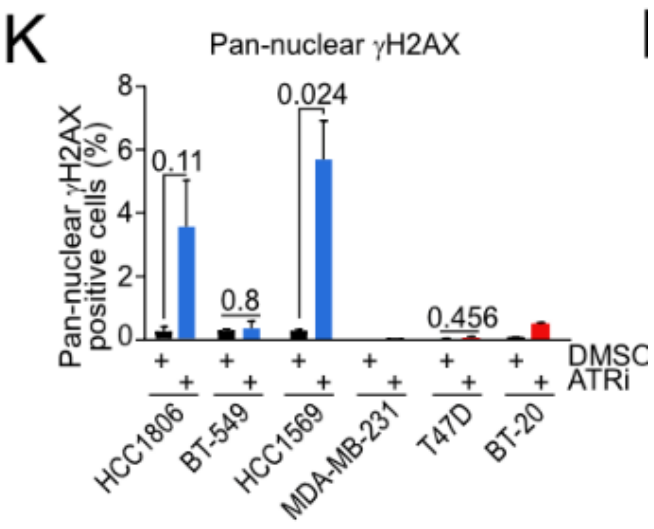

Figure 4k:

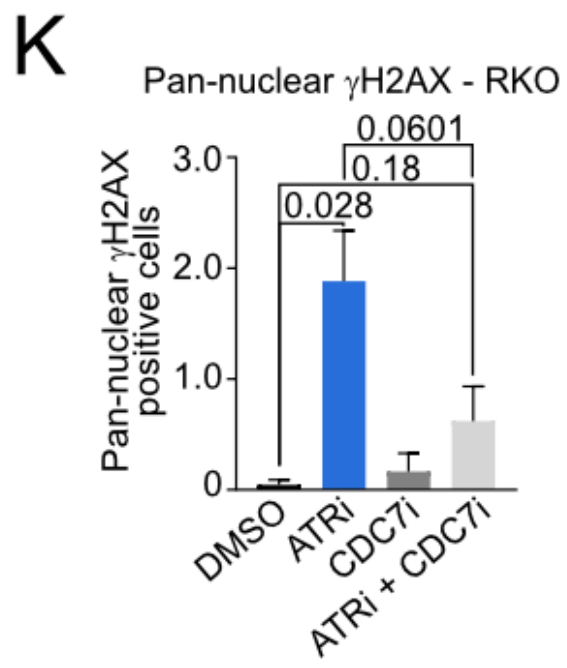

Figure 3d, Extended Figure 3a

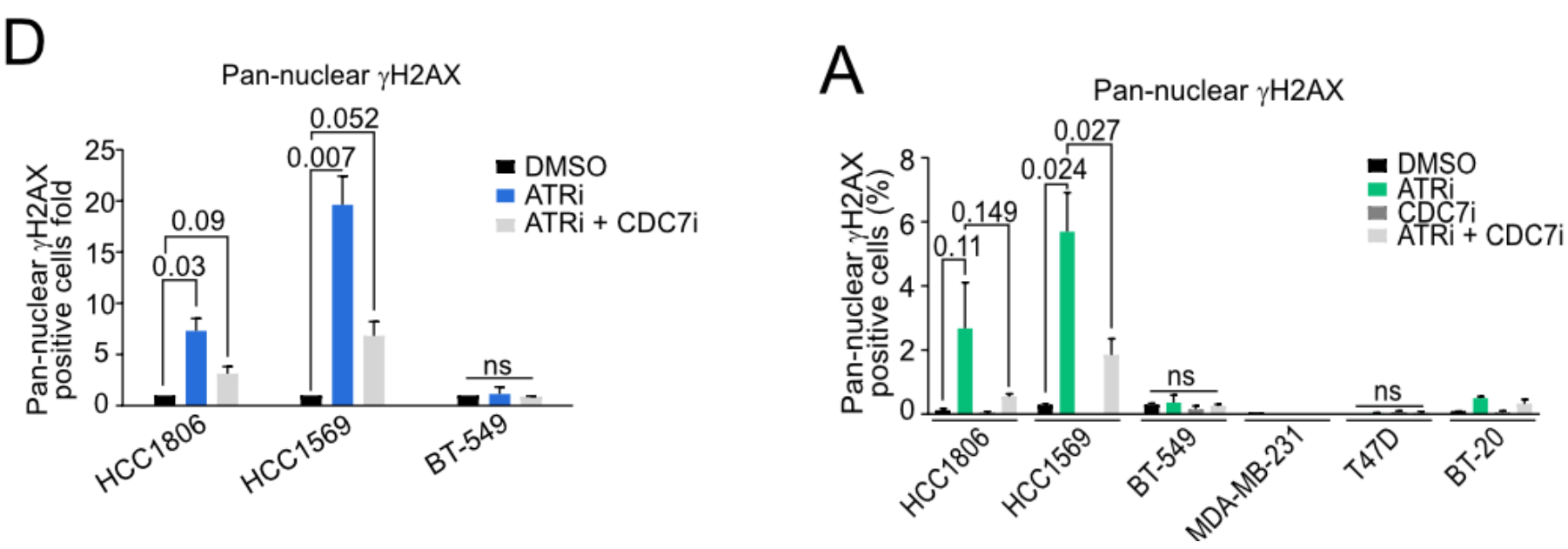

Figure 2f, Extended Figure 2g:

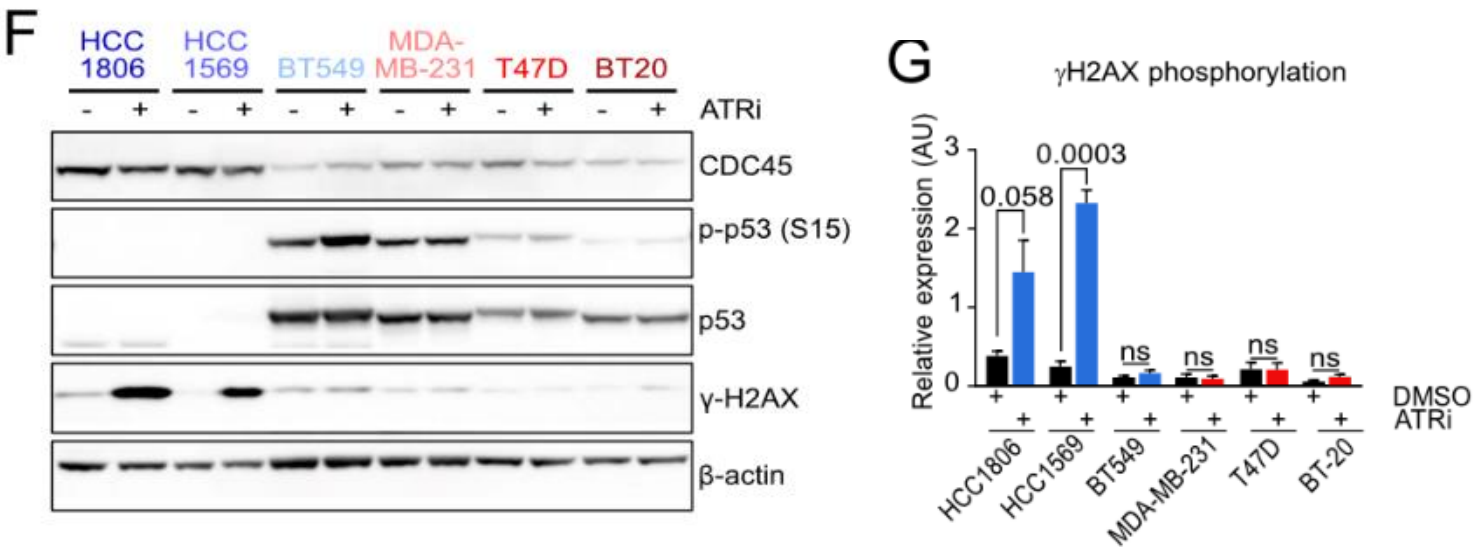

Extended Figure 4d:

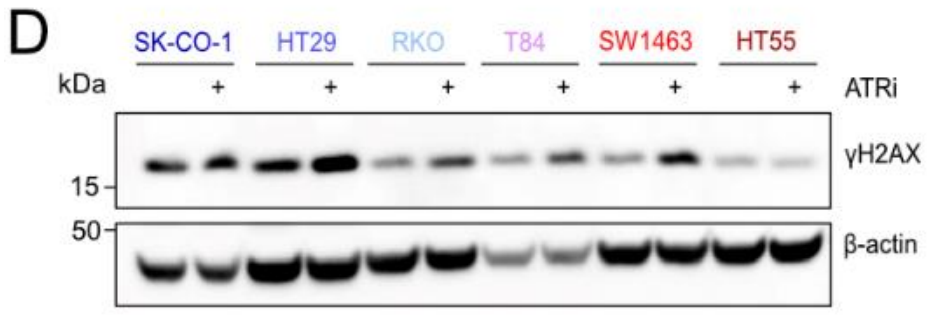

5. Figure 4 is overall unconvincing, extending their previous finding from breast cancer to different cancer types. The authors limited this analysis purely to transcriptomics and the enrichment of the DDR/replication pathway (over other pathways) is not clear (Figure 4C). and no effort was invested confirming the relevance of origin firing (e.g. CDC7) in non-breast tumours. Further support to the relevance of origin firing control seems particularly important for AML, on which the authors focused for the data in Figure 5.

To address this important point, we have now performed additional functional experiments in six colorectal cancer cell lines to test whether the predictions made by our transcriptomic analyses in Figure 4 (revised manuscript - **Figure 4b-c**) would hold true under functional testing. We performed cell viability assays to ATRi treatment, and identified HT55 as the most resistant cell line, SKCO1, HT29 and RKO as the most sensitive cell lines, and T84 and SW1463 as intermediate (revised manuscript – **Figure 4d**). We then performed cell viability assays in response to ATRi with and without CDC7i (revised manuscript – **Figure 4e-i**). We showed that there was no effect of CDC7i nor ATRi on resistant HT55 cell survival. However, For SKCO1, RKO, T84 and SW1463 cell lines, CDC7i partially and significantly rescued ATRi proliferation defects. We then performed immunofluorescence in RKO cells, showing an increase of γH2AX pan-nuclear staining upon 24h ATRi that was rescued by CDC7i (**Figure 4k**).

Figure 4d-i:

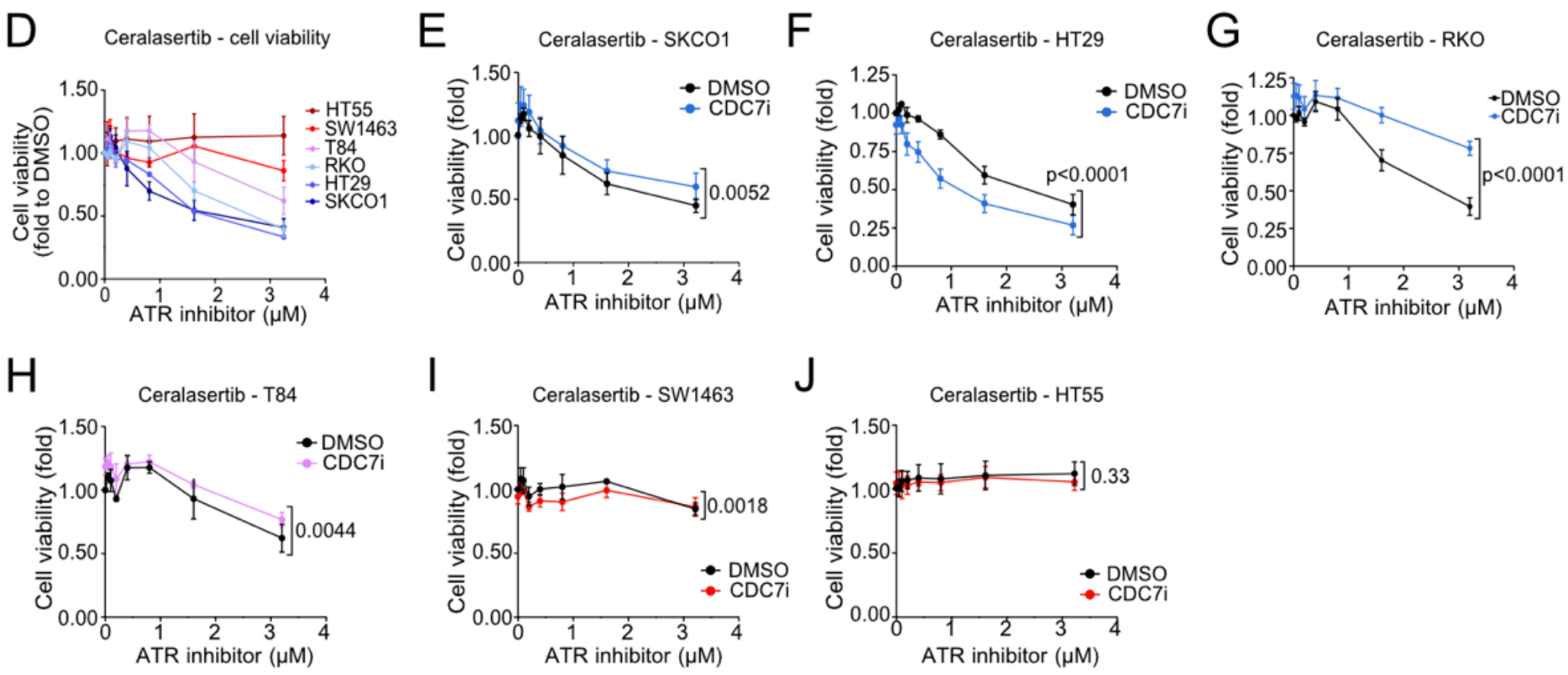

Figure 4k:

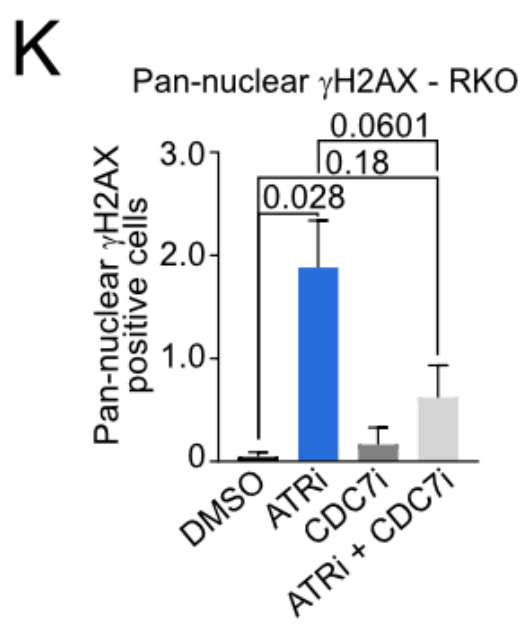

We also performed western blot assays showing a higher expression of CDC45 in RKO, SKCO1, HT29, T84 and SW1463 and very low expression in resistant cell line HT55 (**Revised manuscript - Figure 4m**).

Figure 4m:

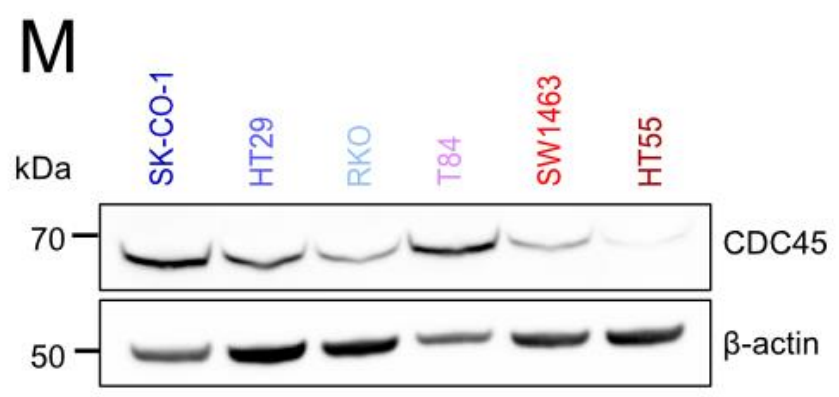

These new data confirm the relevance of origin firing in ATRi sensitivity beyond breast cancer cell lines and is in line with Figure 4 showing that high origin firing factors are associated with sensitivity to ATRi in colorectal cancer, among others.

Regarding strengthening the findings in AML, we were not able within the scope of this study to identify suitable AML cell line panels to test our predictions functionally, however we agree this would be interesting for future studies.

6. The authors suggest in the discussion that sensitizing ATRi-resistant tumors could be possible by manipulating their origin firing capacity. However, their results suggest that sensitization would be achieved by unleashing (rather than preventing) origin firing. This seems contradictory to previous findings cited by the authors (Guo et al.) and also to what the authors claim themselves in the second last paragraph of their discussion. Once/if the authors manage to consolidate the relevance of origin firing levels for ATRi sensitivity, this aspect of their Discussion would need to be adjusted for clarity.

We agree entirely with this prediction and thank the reviewer for pointing out this lack of clarity. Our new results (see point 4a above) demonstrate that we can indeed sensitise resistant cells by unleashing origin firing capacity, which now removes any confusion in the Discussion on this point.

Minor points:

7. In figure 2C, it is not clear whether the displayed fork speed is in presence or absence of ATRi.

Apologies for the confusion, we have now increased clarity of the Figure and legend of the revised manuscript - **Figure 1e,g**.

8. The authors should validate their results in Figures 3B (CDC45), 3D (MCM2 phosphorylation) and 3F (ATM activation) by western blot for the specific markers.

We have now performed western blot assays for CDC45, and for H2AX-Ser139 and p53-Ser15 as the substrates of the identified ATM activated pathway. We also present associated quantifications and statistical analysis in the revised manuscript – **Figure 2f** and **Extended Figure 2c-d, g-h**).

Figure 2f:

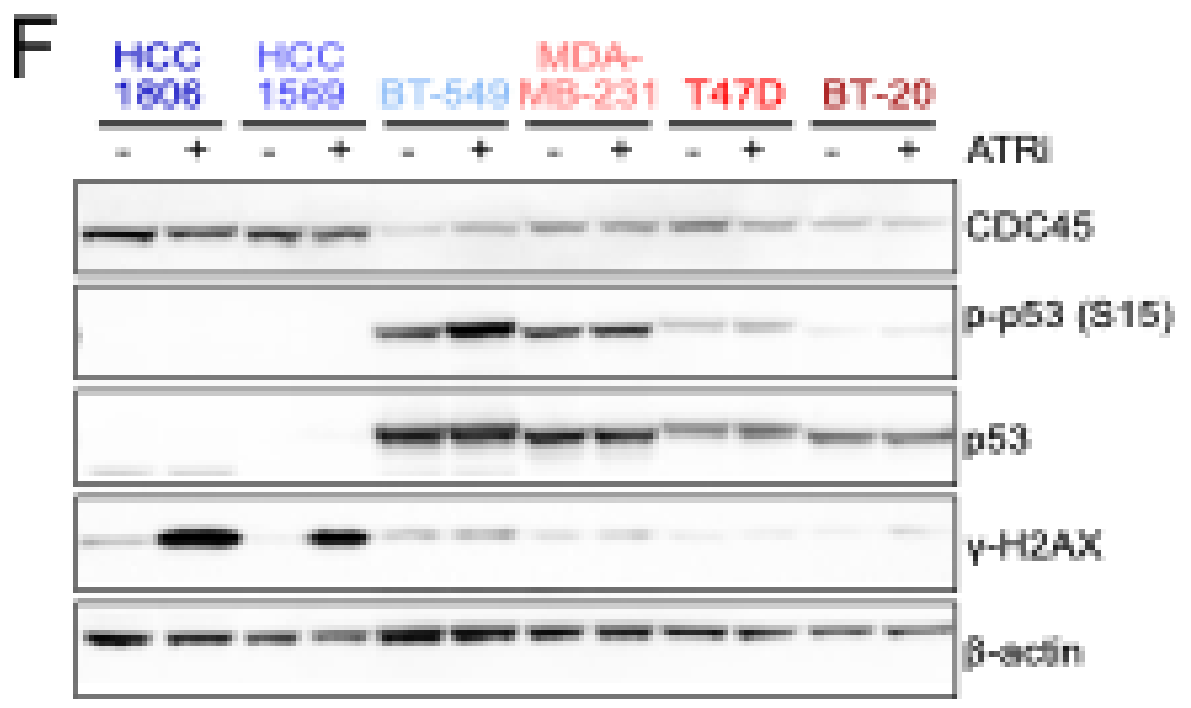

Extended Figure 2c-d, g-h:

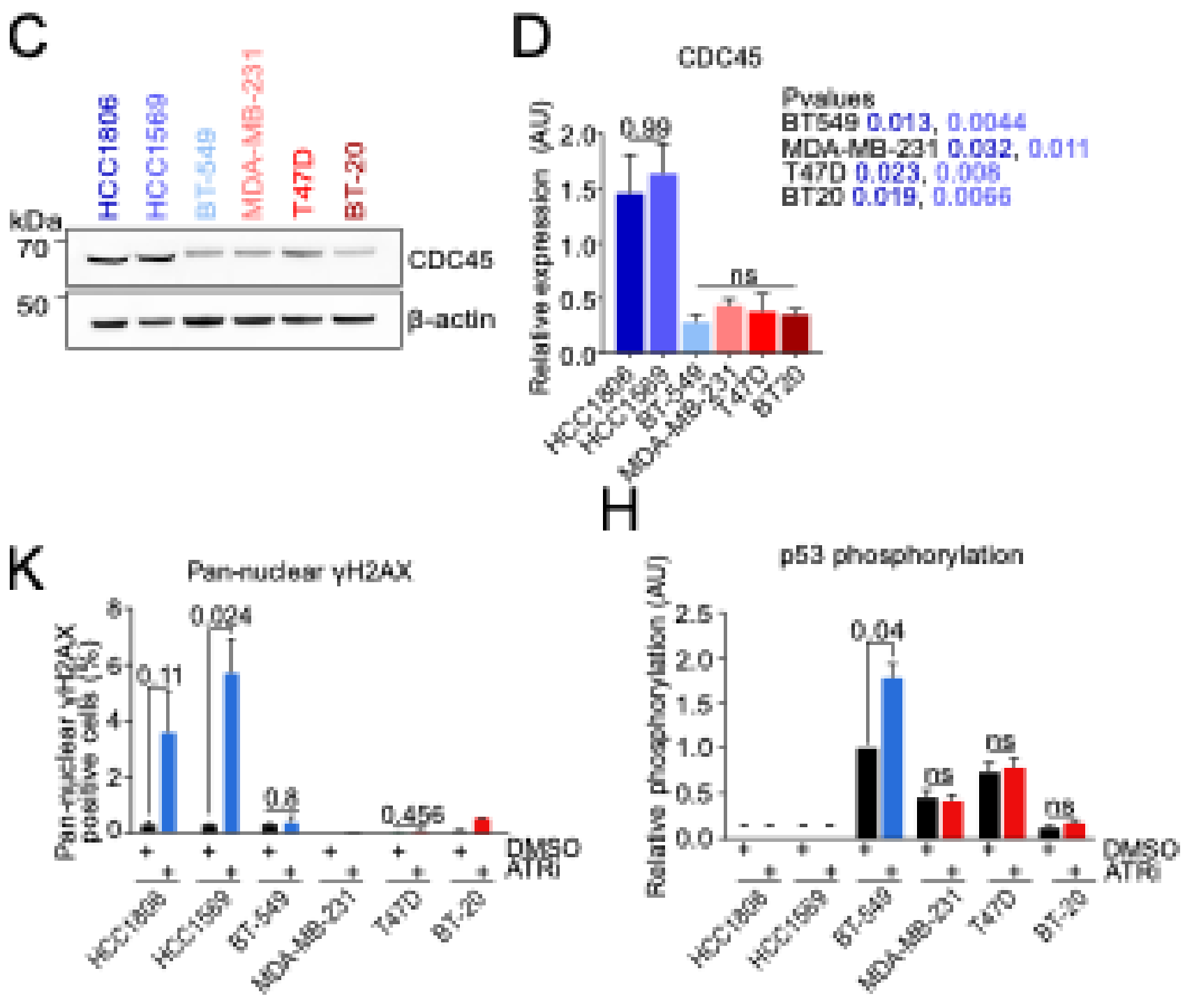

*"To deconvolute the contribution of these alterations from individual cell lines, we performed western blot experiments in the breast cancer cell lines treated with 24h ATRi and showed that the sensitive cell lines with high CDC45 expression (HCC1806, HCC1569) have a strong activation of γH2AX, but did not express p53 (**Figure 2f**). In contrast, BT549 exhibited a strong increase in p53-S15 upon ATRi. These results are in line with the mass spectrometry data showing activation of γH2AX and p53-S15 in response to ATRi in sensitive cells as a collective, but additionally reveals the cell line specific contributions to these signals."*

We also performed western blotting to detect MCM phosphorylation at Ser108 or Ser139, which the mass spectrometry analyses suggested were elevated in sensitive cell lines vs resistant cell lines (**Figure R7** shown below).

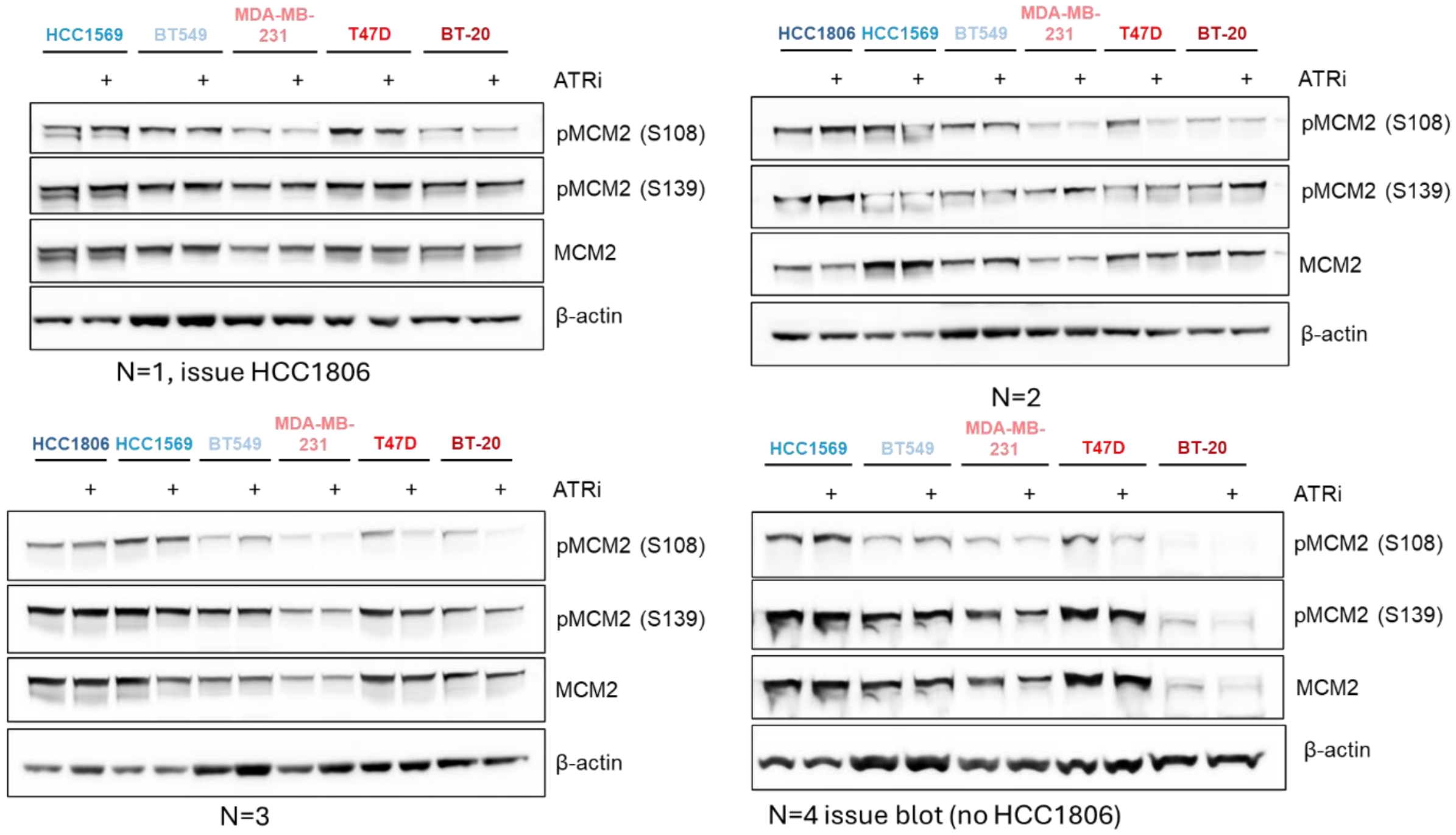

Rebuttal Figure 7. Western blotting of MCM2 phosphorylation at Ser108 and Ser139 and MCM2 protein expression. Beta-actin was used as loading control. Four experimental repeats were performed, and please note the n=1 and 4 blots are missing the data for HCC1806.

Altogether this analysis confirms higher basal levels of MCM2-S108 in the sensitive cell lines compared to the resistant cell lines, while the differences are less clear for MCM2-S139. It is important to note that the mass spec analyses suggesting MCM2 phosphorylation is based on sensitive and cumulative signal from three grouped sensitive cell lines. This may have allowed the S108 site to be picked up by the kinase enrichment analysis in sensitive vs resistant cell lines, while the absolute increase in signal detectable per individual cell line by western blot is minor and below the dynamic range of this technique. Overall, to ensure we are not over-stating the identification of MCM2 phosphorylation increases in the sensitive cell lines, we have now removed specific reference to MCM2 phospho-sites from the manuscript, as we feel the other indicators of the role of origin firing are sufficient.

9. In Figure 3I-K, how do the authors explain a decrease in EdU incorporation upon CDC7i, given that the decrease in OF is compensated by an increase in FR?

Thank you for commenting on that. Indeed CDC7i treatment itself induces a compensatory increase in fork speed which does not seem to result in a full rescue of EdU levels. It is worth noting that our analyses were done after only 1 hr of CDC7i and it is possible that the fork speed increase cannot compensate for total rate of DNA replication in this short time. Overall, it is likely that S phase timing or efficiency is not compromised under these conditions since in our hands, CDC7i treatment for 72h does not impair cell proliferation (see **Figure R8** below).

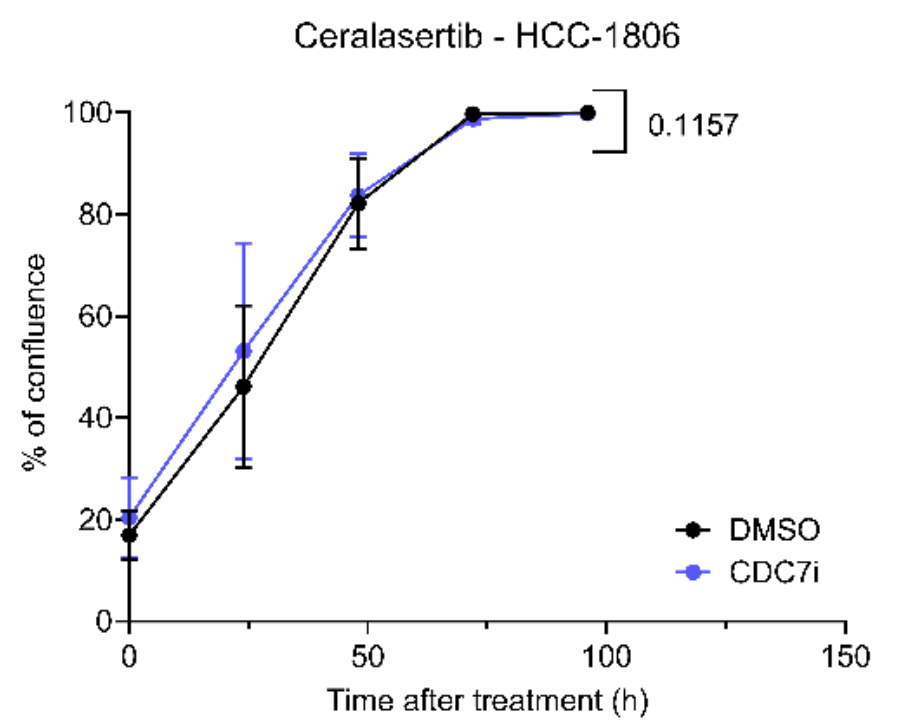

Rebuttal Figure 8. Cell confluence measured in HCC1806 in response to DMSO or CDC7i. Two-way ANOVA test.

10. In figure 4C bottom panel, Mitosis is missing the median bar.

Thank you for pointing this out, this has been corrected in revised manuscript – **Extended Figure 4c**.

11. The authors should always indicate which sensitive and resistant cell lines they use. For instance, in Figure 3O this is not clear. It would also be important to include a Figure to visually explain how the different cell lines for different cancer cell types were segregated in sensitive and resistant from the GDSC2 dataset.

We agree and have now plotted Figure 3o differently (see revised manuscript – **Extended Figure 3a**) and have added figure legend information for when sensitive or resistant cell lines are presented together in a plot.

We have also added a schematic to show how sensitive and resistant cell lines were identified using the GDSC2 AZD6738 IC50 dataset with the quartile method (revised manuscript – **Extended Figure 4a**).

Extended Figure 4a:

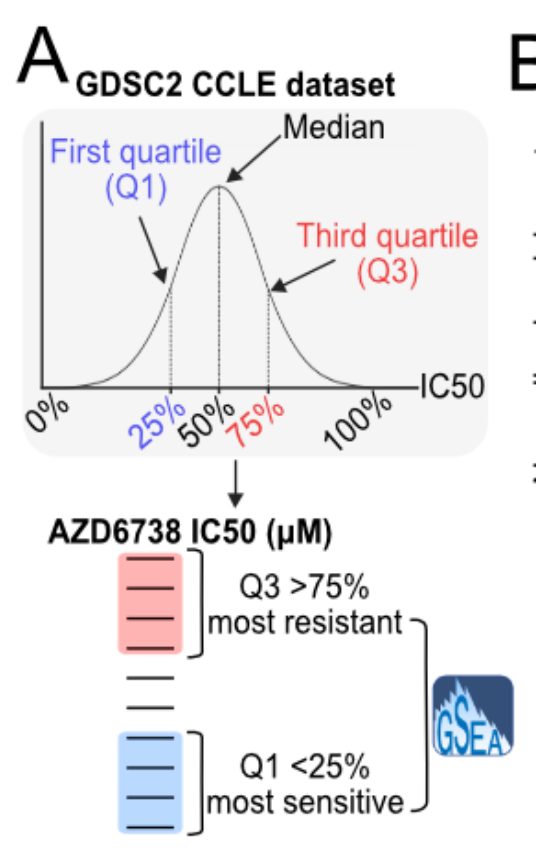

Reviewer #3 (Remarks to the Author):

The manuscript "High levels of DNA replication initiation factors indicate ATRi sensitivity via excessive origin firing" by Lumeau et al. aims to identify prognostic factors indicating sensitivity to ATR inhibition. Since multiple ATR inhibitors are currently undergoing clinical trials, studies like this one are urgently needed to better select the patients who would most benefit from such therapies. Here, the authors show that high expression of origin firing factors predicts increased sensitivity to ATR inhibition due to increased background rate of origin firing. The authors demonstrate this in both breast cancer cell lines

and AML and consistently show very convincing correlations between the increased expression of the origin firing factors and ATRi sensitivity. I believe this is a very valuable study, and should be published, but it would strongly benefit from some direct confirmation experiments.

We thank the reviewer for their support.

Specifically, to confirm that the overexpression of replication initiation proteins directly results in ATRi sensitivity, the authors should overexpress some of those factors in ATRi-resistant cells and confirm that the sensitivity is induced. CDC45 overexpression is long known to induce origin firing on its own (PMID: 26919204), along with some other proteins (PMID: 36516748), any of these can be used.

We agree this is a valuable experiment, especially as we see a correlation between CDC45 protein expression and sensitivity to ATRi (revised manuscript **Figure 2b**, **Extended Figures 2a-d**). We obtained and produced GINS1 and CDC45 overexpression plasmids used in Böhly *et al* study[6]. We first transfected the plasmids in HEK293 cells to test the feasibility and the method (transfections using Lipofectamine 2000 or Jetprime reagents). Unfortunately, GINS1 overexpression did not work in any of the tested cell lines including HEK293 (no detection of GINS1 nor Myc-Tag) so we focused on EGFP-CDC45 overexpression.

We transfected resistant T47D, MDA-MB-231 and BT20 breast cancer cell lines and performed western blots to assess exogenous protein expression (**Figure 3i**), as well as viability assays to assess the response to ATRi. Our new results showed that while CDC45-GFP overexpression alone did not impact cell viability (revised manuscript **Extended Figure 3f**), we observed a modest but significant decrease of cell viability in response to ATRi when CDC45 is overexpressed in MDA-MB-231, T47D and BT20 (revised manuscript - **Figure 3i-l**).

Figure 3i:

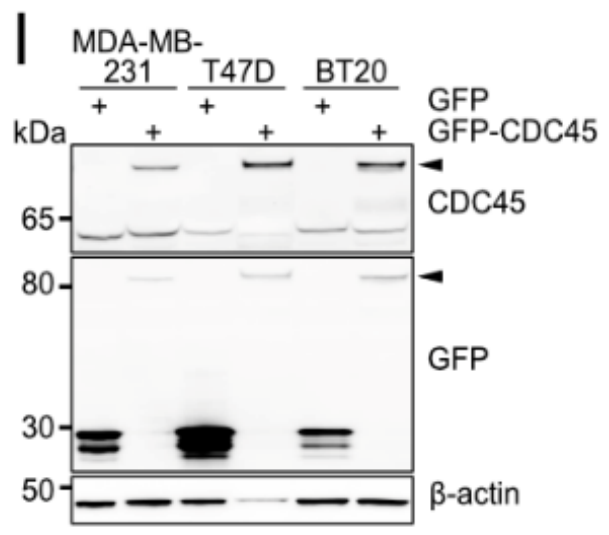

Extended Figure 3f:

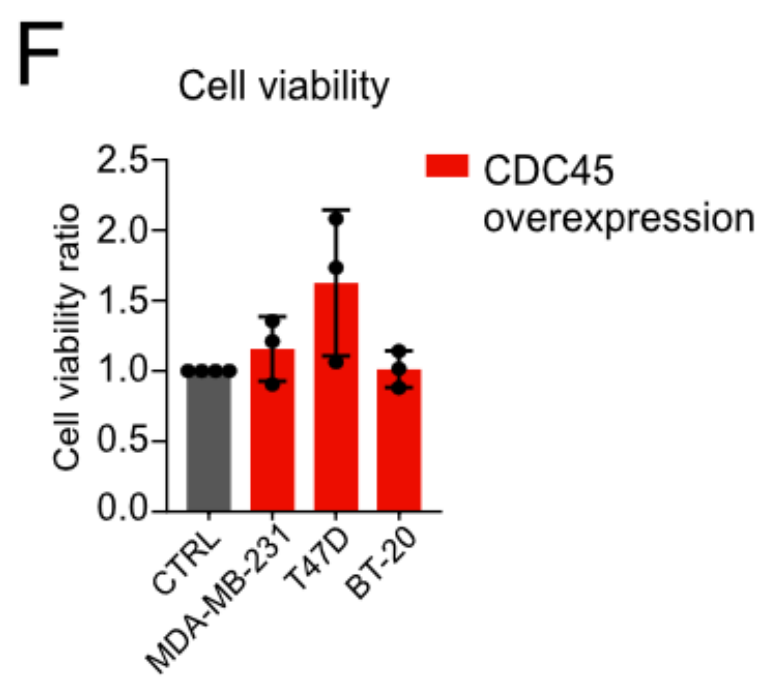

Figure 3j-l:

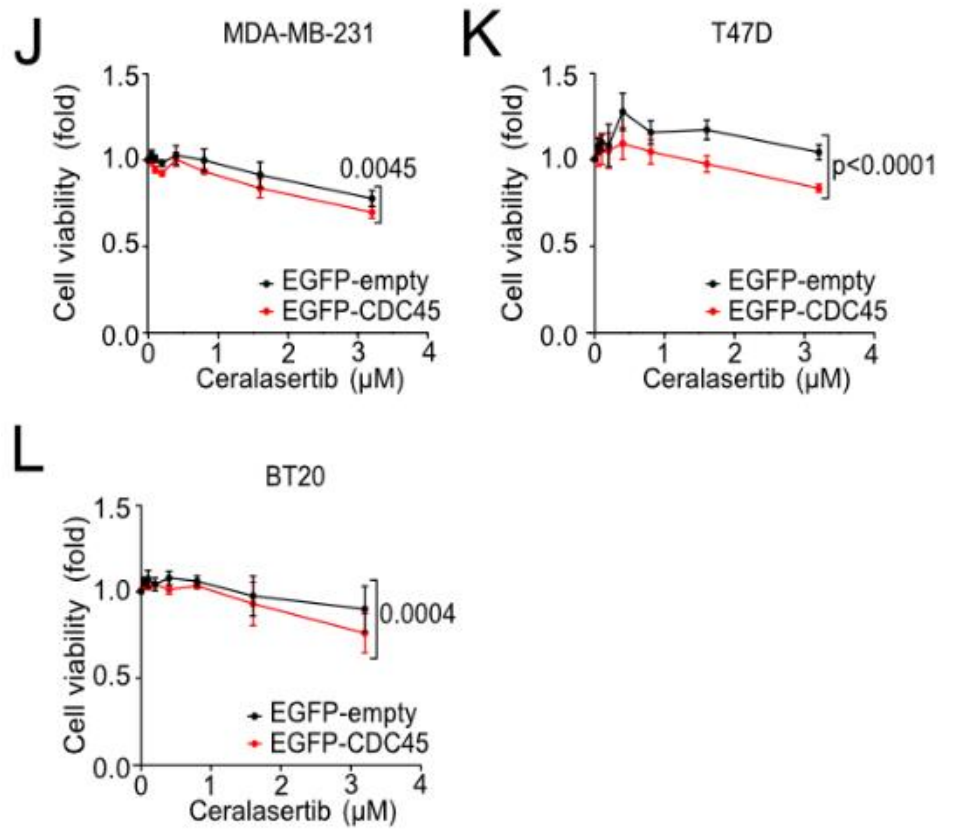

This shows that treating resistant cells with these specific doses of ATR and CDC7 inhibitors in combination decreases cell viability. We have added these new data into the results along with the new figures presented above:

"We next reasoned that, if resistant cell lines are able to escape ATRi-mediated cell death by limiting excessive origin firing, then inducing higher origin firing rates in ATRi resistant cells might reverse ATRi resistance. To investigate this, and given that CDC45 is already highly expressed in sensitive cell lines (Figure 2b, Extended Figure 2a-d), we transiently overexpressed CDC45 (as done previously to increase origin firing[11]) in the three resistant breast cancer cell lines MDA-MB-231, T47D and BT-20. Western blot assays confirmed the increase of expression of CDC45 protein levels in all three cell lines (Figure 3i). Interestingly, CDC45 overexpression led to a mild but significant sensitization to ATRi in all three resistant cell lines (Figure 3j-l), while having no impact on cell viability in the absence of ATRi (Extended Figure 3f). This demonstrates that origin firing must be tightly regulated in cancer cell lines to resist ATRi, since decreasing origin firing (with CDC7i) or increased origin firing (with overexpression of CDC45) could both reduce the viability of cell lines initially resistant to ATRi, particularly exemplified by T47D which was sensitive to both manipulations (Extended Figure 3b,d,e Figure 3i,k). Altogether our results provide evidence that origin firing capacity is an important factor in determining sensitivity to ATRi and that manipulating origin firing can modulate the ATRi response in both directions."

Also, the authors provide extensive supplementary tables, but they are not fully annotated, and not all of thems are referenced in the text of the manuscript. Perhaps, they could be referenced in the methods if the data in them are not directly discussed in the manuscript (for example, sup table 5 vs sup table 6).

We apologise for this and have now cited all supplementary tables.

References

(1) *Home | ClinicalTrials.gov*. https://clinicaltrials.gov/ (accessed 2025-11-11).

(2) Rafiei, S.; Fitzpatrick, K.; Liu, D.; Cai, M.-Y.; Elmarakeby, H. A.; Park, J.; Ricker, C.; Kochupurakkal, B. S.; Choudhury, A. D.; Hahn, W. C.; Balk, S. P.; Hwang, J. H.; Van Allen, E. M.; Mouw, K. W. ATM Loss Confers Greater Sensitivity to ATR Inhibition than PARP Inhibition in Prostate Cancer. *Cancer Res* **2020**, *80* (11), 2094–2100. https://doi.org/10.1158/0008-5472.CAN-19-3126.

(3) Dreyer, S. B.; Upstill-Goddard, R.; Paulus-Hock, V.; Paris, C.; Lampraki, E.-M.; Dray, E.; Serrels, B.; Caligiuri, G.; Rebus, S.; Plenker, D.; Galluzzo, Z.; Brunton, H.; Cunningham, R.; Tesson, M.; Nourse, C.; Bailey, U.-M.; Jones, M.; Moran-Jones, K.; Wright, D. W.; Duthie, F.; Oien, K.; Evers, L.; McKay, C. J.; McGregor, G. A.; Gulati, A.; Brough, R.; Bajrami, I.; Pettitt, S.; Dziubinski, M. L.; Candido, J.; Balkwill, F.; Barry, S. T.; Grützmann, R.; Rahib, L.; Glasgow Precision Oncology Laboratory,; Australian Pancreatic Cancer Genome Initiative; Johns, A.; Pajic, M.; Froeling, F. E. M.; Beer, P.; Musgrove, E. A.; Petersen, G. M.; Ashworth, A.; Frame, M. C.; Crawford, H. C.; Simeone, D. M.;

Lord, C.; Mukhopadhyay, D.; Pilarsky, C.; Tuveson, D. A.; Cooke, S. L.; Jamieson, N. B.; Morton, J. P.; Sansom, O. J.; Bailey, P. J.; Biankin, A. V.; Chang, D. K. Targeting DNA Damage Response and Replication Stress in Pancreatic Cancer. *Gastroenterology* **2021**, *160* (1), 362-377.e13. https://doi.org/10.1053/j.gastro.2020.09.043.

(4) Shi, Q.; Shen, L.-Y.; Dong, B.; Fu, H.; Kang, X.-Z.; Yang, Y.-B.; Dai, L.; Yan, W.-P.; Xiong, H.-C.; Liang, Z.; Chen, K.-N. The Identification of the ATR Inhibitor VE-822 as a Therapeutic Strategy for Enhancing Cisplatin Chemosensitivity in Esophageal Squamous Cell Carcinoma. *Cancer Lett* **2018**, *432*, 56–68. https://doi.org/10.1016/j.canlet.2018.06.010.

(5) Barnieh, F. M.; Loadman, P. M.; Falconer, R. A. Progress towards a Clinically-Successful ATR Inhibitor for Cancer Therapy. *Current Research in Pharmacology and Drug Discovery* **2021**, *2*, 100017. https://doi.org/10.1016/j.crphar.2021.100017.

(6) Böhly, N.; Schmidt, A.-K.; Zhang, X.; Slusarenko, B. O.; Hennecke, M.; Kschischo, M.; Bastians, H. Increased Replication Origin Firing Links Replication Stress to Whole Chromosomal Instability in Human Cancer. *Cell Reports* **2022**, *41* (11), 111836. https://doi.org/10.1016/j.celrep.2022.111836.

Lumeau et al, Rebuttal for *"Replication origin firing capacity indicates ATR inhibitor sensitivity"*

We thank all reviewers for their careful reading of our revised manuscript**.** We are very happy that Reviewers 1 and 3 appreciated the improved revised manuscript and entirely support publication in Nature Communications without further edits to the manuscript. Reviewer 2 raised some additional concerns to be addressed. We thank the editors for allowing us to submit a second revised manuscript, and we hope that Reviewer 2 will find the new experiments further support our central hypotheses.

Reviewer #1 (Remarks to the Author)

The authors have gone to significant lengths to improve the manuscript and to address each of the points raised in the 1st review. I have no other comments to make other than I think the manuscript will make an impressive addition to the literature and be read and highly cited, especially as it tells us a lot about a drug class currently undergoing clinical trial assessment. I think the journal should publish at once.

> We thank the Reviewer kindly for their support.

Reviewer #2 (Remarks to the Author):

I appreciate that the authors consolidated the difference in ATRi sensitivity among the breast cancer lines they selected, extending their analysis to clonogenic and cell viability assays.

> We thank the Reviewer for this.

However, I remain largely unconvinced – in fact even less convinced than from the originally submitted manuscript – of the functional link between origin firing and ATRi sensitivity. In a nutshell, key experiments were not performed (with questionable arguments) and those that were performed led to rather controversial data, which do not provide significant support to the central conclusion of this manuscript, but rather challenge it further.

> We hope that, with the additional experiments presented below - namely the requested addition of roscovitine experiments which further support our hypothesis regarding origin firing and ATRi sensitivity, and a deeper investigation into the initially puzzling CDC45 depletion experiment - we have alleviated these concerns.

Besides a few minor points (that are omitted here), these are the main concerns on the revised manuscript:

Point 2 (of my original comments): the explanation of the increased γH2AX in sensitive cell lines as "pan-nuclear" signal uncoupled from DNA damage is purely speculative and not substantiated by any data. Moreover, now that careful statistical assessment has been done on micronuclei levels, the data clearly reveal upon ATRi a significant increase in the sensitive lines, which is not significant for the resistant ones. Hence, even though the authors chose a vaguer formulation in their text ("…does not robustly correlate with exacerbated…"), the conclusion that sensitivity is NOT related to increased CIN and genomic instability is essentially challenged by their own new data.

> We apologise for any confusion regarding the wording our previous rebuttal to this point. We did not mean to imply that γH2AX (pan-nuclear or otherwise) is necessarily uncoupled from DNA damage signalling. Instead, we meant that because the only indicator of DNA damage signalling we observed across all three sensitive breast cancer lines was γH2AX signal (by Mass spec, western blot, and pan-nuclear signal by imaging), we could not formally implicate downstream DNA damage signalling downstream of γH2AX *per se*. Importantly, this point does not impact the main conclusion of the manuscript, which connects an increased capacity of origin firing upon ATRi treatment to cellular sensitivity. We were therefore careful not to state any specific role of DNA damage to this phenotype in the text of the manuscript.

Regarding the new statistical analyses of micronucleus formation, we agree that increased formation of MN remains a potential driver of ATRi sensitivity in sensitive breast cancer cell lines. We had not focussed on this specific aspect in our study as increased MN formation was the only significant indicator of increased CIN in the ATRi sensitive cell lines, and as we were interested in drivers that could be detected at basal levels (prior to ATRi) whereas the increased MN formation only becomes apparent upon ATRi. We have now updated the text in the introduction and result sections to note the significant MN increase in sensitive lines and to reiterate our goal of detecting a predictive marker of ATRi sensitivity:

<u>End of introduction:</u>
"All cell lines experienced similar increases in RS and CIN upon ATR inhibition, though sensitive lines exhibited a higher micronucleus rate, suggesting a potential contribution of excessive micronuclei rates to ATRi sensitivity. However, none of these RS nor CIN markers could predict the response of breast cancer cell lines to ATRi, prompting us to explore additional molecular mechanisms responsible for cell death in sensitive cell lines."

<u>Results section:</u>
*"Baseline rates of CIN were not significantly different between the sensitive and resistant lines (**Figure 1l-o, Extended Figure 1o**), and ATRi treatment caused similar relative increases in segregation errors and micronuclei across all cell lines (**Figure 1p**). The universal increase in CIN following ATRi treatment also excludes differences in drug uptake or efflux as a cause of differential ATRi sensitivity. <u>We noted that absolute micronuclei rates were significantly higher after ATRi in sensitive cells (**Figure 1l, Extended Figure 1o-q**), potentially contributing to ATRi sensitivity in sensitive breast cancer cell lines. However, overall, baseline rates of CIN or RS could not readily predict the differential ATRi sensitivity of this panel of breast cancer cell lines."</u>*

Point 3: MCM2 phosphorylation is well known to reflect origin firing (as opposed to licensing). Hence it is not really clear what the authors are proposing, when suggesting that the sensitive lines are "primed" to achieve higher rates of origin firing only upon ATR inhbition. In essence, along with the other problematic points, these data contribute to challenge the link between sensitivity, P-MCM2 and excessive origin firing. Providing a possible, yet unsupported explanation for this discrepancy – as the authors do in their rebuttal – does not really help substantiating the main conclusion of the study.

> We apologise for any confusion resulting from our previous rebuttal. We agree that specific phosphorylation of MCM2 reflects a higher origin firing rate. Accordingly, in the manuscript we had proposed only that the inability to detect higher origin firing at basal states (without ATRi present) is likely due to lack of sufficient sensitivity in the experimental methods used:

Original manuscript text: *"To determine whether the high origin firing factor expression levels in sensitive cell lines led to an elevated increase in replication origin firing upon ATRi, we analysed the frequency of DNA replication origins per megabase of DNA (origin density) in our DNAscent data. No*

difference in origin density was detected between sensitive and resistant cells in untreated conditions, though it is likely that the limit of detection for this assay precludes this observation (Figure 2g)".

The reason we developed the 'primed state' idea that we discussed in the rebuttal only was that, given the lack of ability to confirm basal origin firing due to limits of the assay, **we speculated that 'a primed origin firing' state rather than basally elevated origin firing could also explain our data, given a strong association with increased replication factor expression levels in addition to a potential increase in MCM2 phosphorylation**. However we agree this is highly speculative and have not then, or now, alluded to this in the manuscript, and we apologise for any confusion resulting from this speculation.

Points 4/5: despite possible off-target effects – which are in fact also a possible problem for CDC7i – roscovitine has been previously used in multiple publications that initially showed deregulated origin firing by ATR/CHK1 inhibition or inactivation (see for example PMID: 20805465 or PMID: 21173116). Hence, it is not understandable why the authors refrained from performing this simple and essential control experiment that may (or may not) have backed up their conclusions from CDC7i treatments. Moreover, their new siCDC45 data are puzzling and worrying. Such an effective depletion of an essential initiation factor is expected to affect origin firing significantly (something that the authors did not directly assess). The lack of any effect of such a perturbation on the sensitivity of their lines (Rebuttal Fig. 6) is definitively at odd with the key conclusion of this study. In this context, arguing without even testing it directly that "it is probably very technically challenging to reduce origin firing by manipulating relevant protein levels in cells" is not really useful to resolve such a crucial discrepancy and to increase confidence in the manuscript conclusions.

> We apologise for not originally including experiments with roscovitine to inhibit CDK2 and so reduce origin firing by an independent means. We entirely agree that this is an important orthogonal validation of our central hypothesis, and we only hesitated due to known off-target effects[1,2] that might be more problematic over longer-term experiments, and concerns that inhibiting cell cycle progression would prevent long-term assays required to assess cell viability changes. Indeed, the roscovitine doses used in these prior publications were toxic to our cell lines in our initial experiments (data not shown). However, we were able to titrate down the concentration of roscovitine to allow efficient reduction of origin firing without compromising cell viability (**Extended Data 3h and 4e**). Using these conditions, we were excitingly able to show a strong reduction of ATRi sensitivity as the reviewer had predicted by lowering origin firing capacity.

We have now performed the experiments as suggested in two ATRi-sensitive cell lines (HCC1806 to represent breast cancer and RKO to represent colorectal cancer). We also performed identical experiments with a new, specific CDK2 inhibitor from AZ; AZD8421[3]. Both CDK2 inhibitors decreased origin firing and prevented ATRi-induced hyperactive origin firing, restoring origin firing to near-normal levels upon ATRi treatment (**Figure 3A, I, Extended Figure 4F**):

Figures 3A,I:

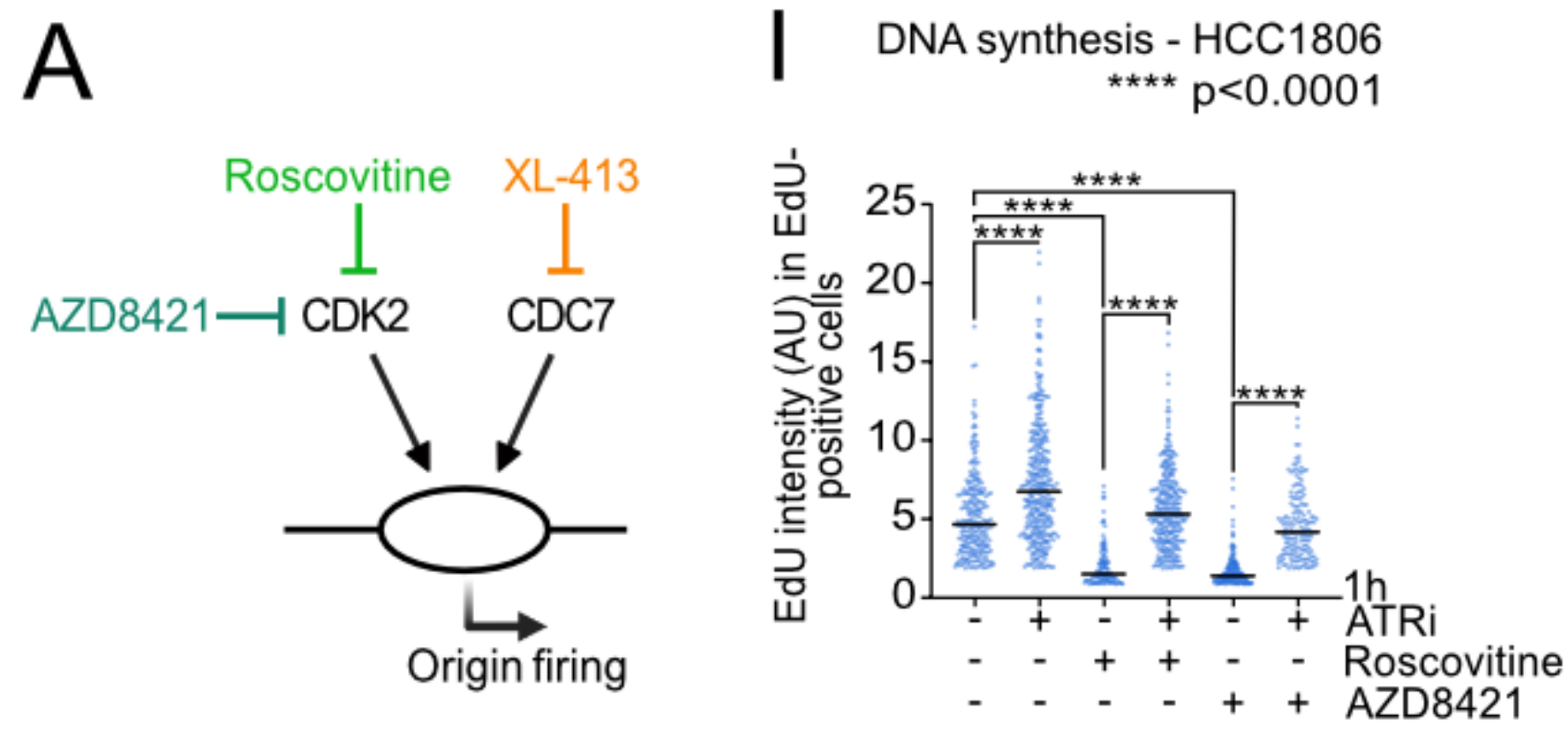

Importantly, both inhibitors also efficiently reduced ATRi sensitivity of both cell lines as measured by cell proliferation and cell viability assays, in fact even more efficiently than our previous CDC7i experiments (**Figure 3J-K, Extended Figures 3F-G, Figure 4M-N**):

Figure 3J-K:

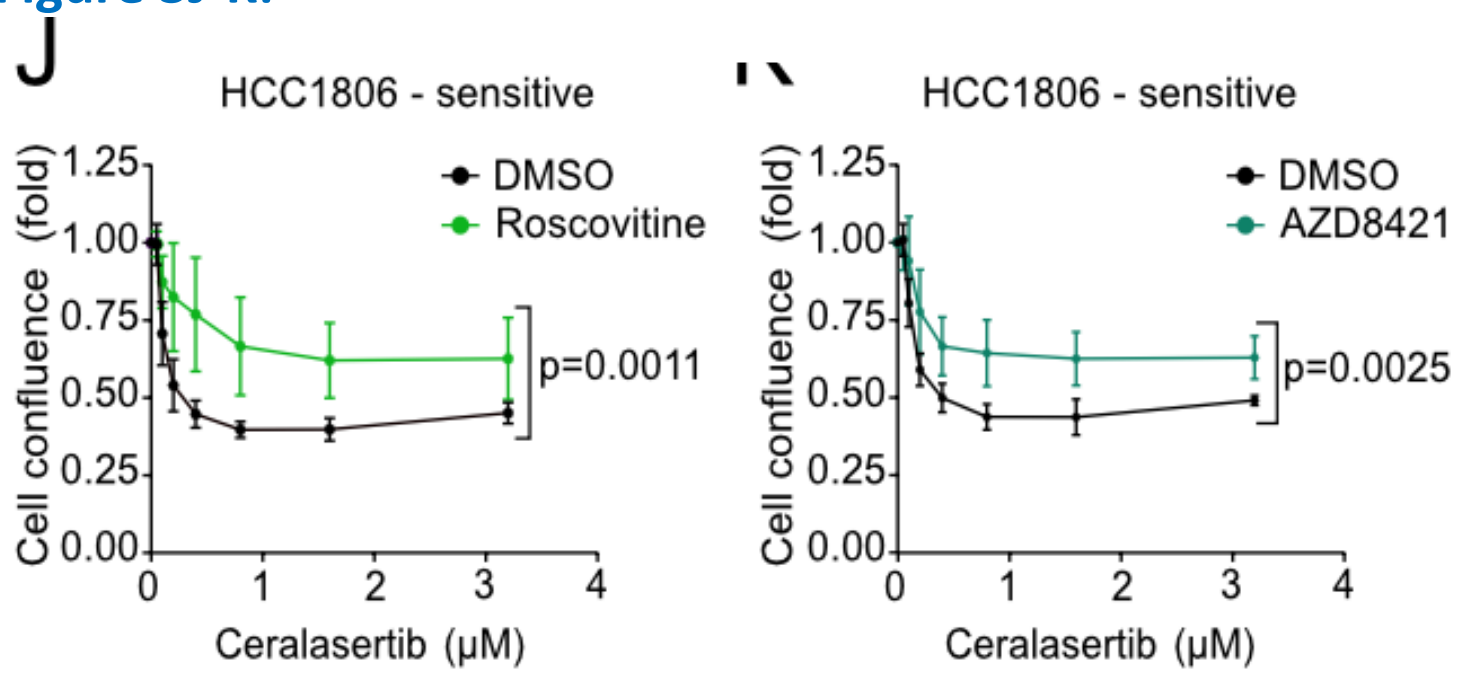

Extended Figure 3F-G:

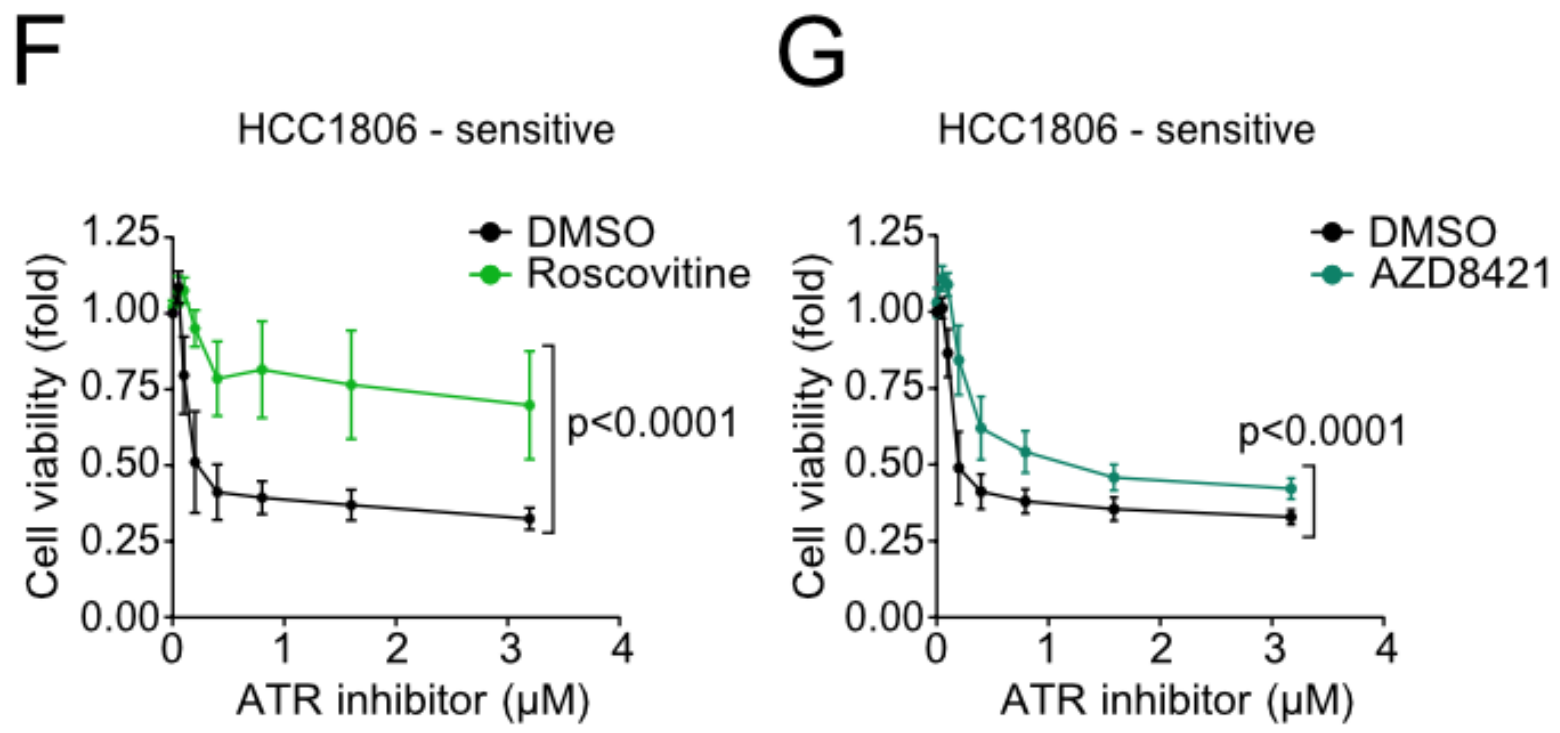

Figure 4M-N:

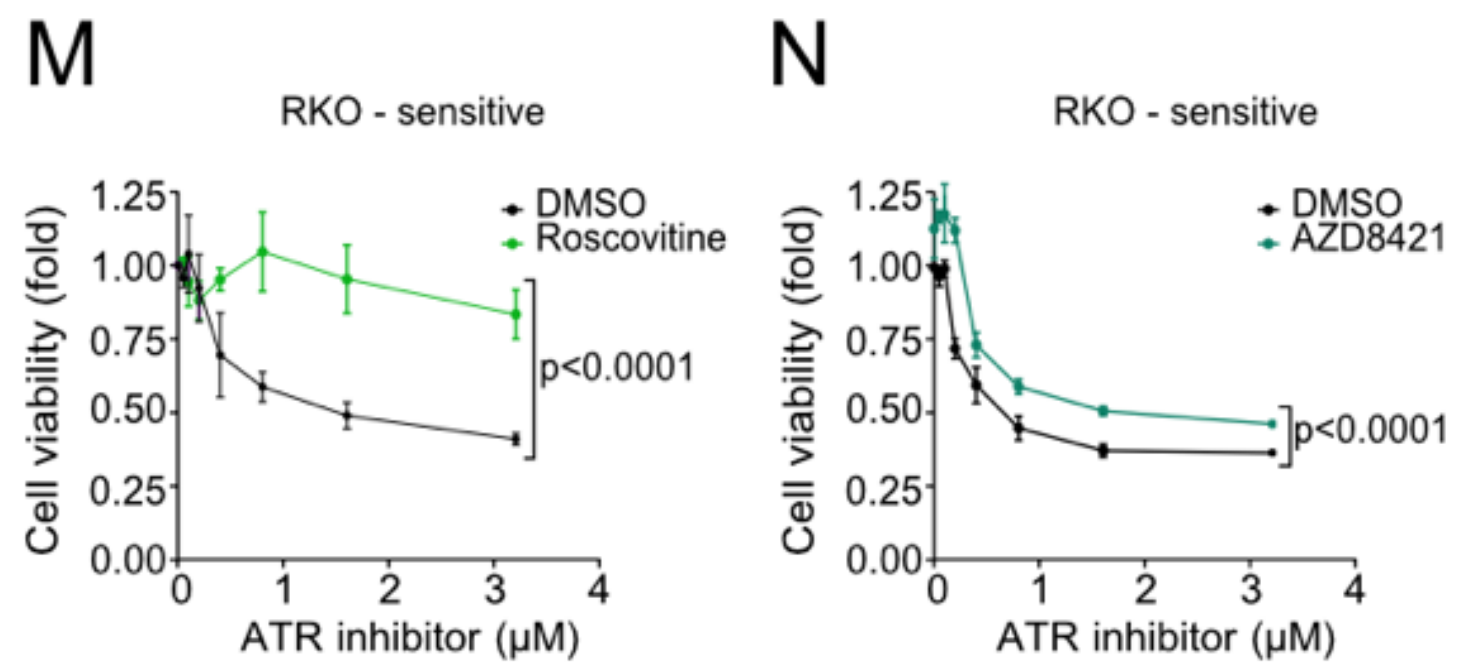

We thank the reviewer for their perseverance in encouraging us to complete these experiments as we feel these data have strongly improved the robustness of our original findings. **The manuscript's central hypothesis is now supported by the use of three different kinase inhibitors and two independent protein targets (CDC7 and CDK2) to reduce origin firing and decrease sensitivity to ATRi in two cancer types.**

Regarding the initially confusing CDC45 depletion experiments (which were presented in the previous rebuttal as an alternative approach to manipulating origin firing), we appreciate that our preliminary attempts to reduce origin firing using siRNA to deplete CDC45 protein levels led to a confusing observation that was apparently at odds with our other data, namely that reducing CDC45 levels significantly did not cause observable decrease in ATRi sensitivity, in fact sensitising them further (**Rebuttal Figure 1a,b**). To explore the reason for this result, we repeated siRNA experiments and measured origin firing using EdU foci. CDC45 depletion by siRNA was efficient, and reduced origin firing levels as expected (**Rebuttal figure 1a,b**). Interestingly however - *and in direct contrast to inhibition of CDK2 or CDC7* - ATRi treatment on top of CDC45 depletion failed to restore origin firing to normal levels (**Rebuttal Figure 1c**). This is the likely explanation for why siCDC45 is toxic when combined with ATRi, as this presents cells with a condition where origin firing levels are completely prevented from increasing in a compensatory manner after ATRi, presumably leading to cell death, though we did not investigate this further given the new CDK2 inhibition data.

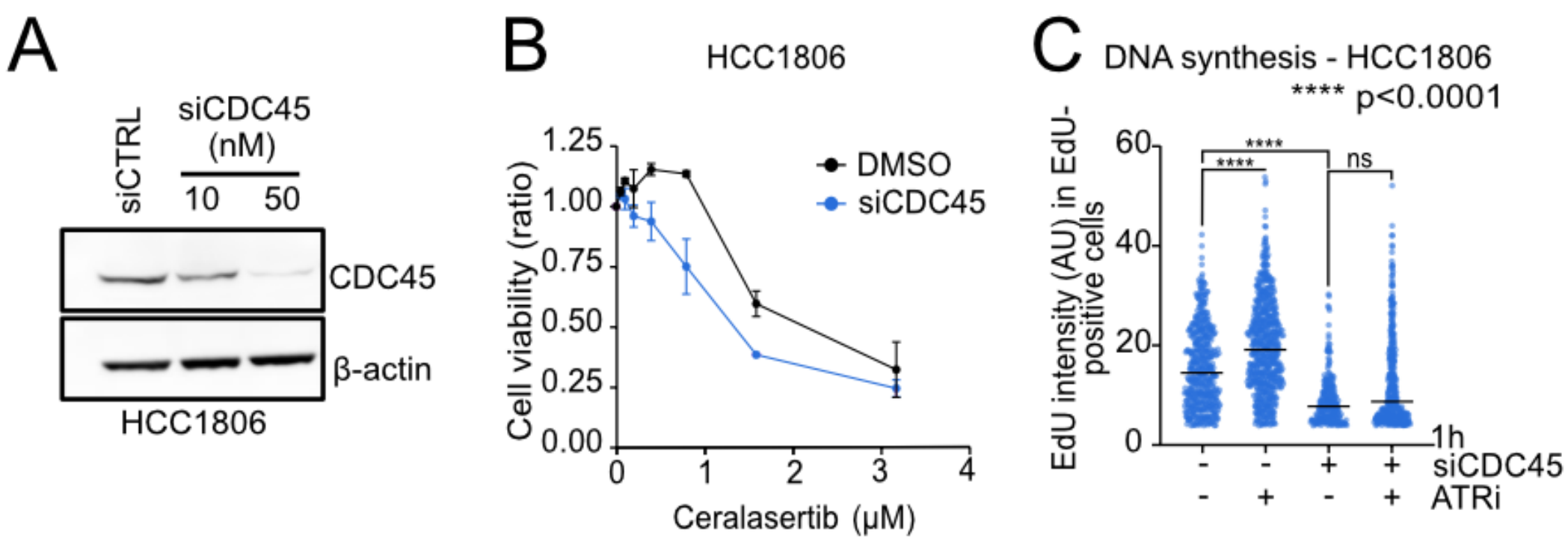

Rebuttal Figure 1. (**A**) CDC45 expression in HCC1806 48h after transfection with siCTRL or siCDC45 (24h post-transfection). (**B**) Cell viability after 72 hours of ATRi in HCC1806 siCTRL and siCDC45 using CellTiterGlo. Results are representative of two independent experiments. (**C**) EdU intensity per cell in HCC1806 siCTRL and siCDC45 treated with DMSO or ATRi for 1h. Results are representative of two independent experiments.

These observations explain our initial confusing observations with CDC45 depletion presented in the previous rebuttal. In combination with the new CDK2 inhibitor experiments that provide orthogonal validation of our central hypothesis that reducing hyper-increased origin firing upon ATRi treatment rescues sensitivity, we have not pursued this line of investigation further and no data relating to CDC45 depletion are in the manuscript.

Inspired by the comment of a different reviewer, the authors performed the opposite experiment (CDC45 OE), but the results they obtained (Fig. 3i-l) are unconvincing. Although the statistics seems to reveal significant differences between the displayed curves (which is surprising per se), the effects are once again marginal, and by no means sufficient to make a resistant cell line even close to the response observed in sensitive lines. Similarly, extending the use of CDC7i to the colorectal cell lines (Fig. 4d-i) generally showed marginal effects on sensitivity (in some cases even discrepant results, e.g. Fig. 4F).

Once again, these marginal effects of CDC7i also in this context can hardly explain in terms of origin firing the differential sensitivity observed among colorectal cancer cell lines.

> We agree the response to CDC45 overexpression is moderate. CDC45 is one of many DNA replication genes that we showed was upregulated in the sensitive lines, so we did not anticipate a full rescue using a single gene overexpression. Instead we are encouraged to see a modest, yet reproducible and statistically significant effect which also satisfied the reviewer who had specifically asked for this experiment.

> CDC7 inhibition indeed showed modest, but importantly reproducible effects in most CRC cell lines (we also note that RKO showed the highest rescue of cell viability of all tested cell lines). Combined with our new data with CDK2 inhibitors we are very confident that manipulating origin firing rates can modulate ATRi sensitivity, which is a central claim of our manuscript.

> Our new data using CDK2 inhibitors in breast cancer HCC1806 and colorectal RKO cell lines robustly confirm our previous experiments using CDC7i. Altogether, our manuscript shows through different cell lines and with three different inhibitors the contribution of origin firing in sensitivity to ATRi. We indeed propose that origin firing is *one* new predictive factor of sensitivity to ATRi, and we have shown that - even if the effect of CDC7i is modest is some cell lines - overall, the experimental decrease of origin firing improves cell survival in response to ATRi.

Overall, even considering all new data added in revision, the entire dataset in the manuscript at best suggests that origin firing has a minor contribution to ATRi sensitivity. However, it does not seem that any of the biomarkers or functional readouts explored by the authors for origin firing could effectively be used to predict ATRi response in cancer cells, which is the key conclusion proposed in the manuscript. Hence, the authors should be encouraged to drastically tone down their conclusions, while submitting a selection of their results to a more specialistic journal.

> We hope that with the additional data presented above the reviewer will be able to re-evaluate our manuscript as a whole. Our central claims are that (i) ATRi sensitivity is in part due to origin firing capacity, which we now support using three independent inhibitors to manipulate origin firing. (ii) Indicators of origin firing capacity such as gene expression of DNA replication factors have the potential to be developed into a clinically-applicable predictor of response to ATRi. Our data in Figures 4 and 5 show this association in several cancer types (colorectal cancer, breast cancer, oesophageal carcinoma, PDAC). We have discussed in the manuscript how the gene expression signature did not predict sensitivity well for certain cancer types, and suggested that cancer-specific signatures would be more appropriate for future biomarker investigations.

Reviewer #3 (Remarks to the Author)

The authors sufficiently addressed my comments and I believe the manuscript can now be published.

> We thank the Reviewer for their support

Reviewer #4 (Remarks to the Author)

I co-reviewed this manuscript with one of the reviewers who provided the listed reports. This is part of the Nature Communications initiative to facilitate training in peer review and to provide appropriate recognition for Early Career Researchers who co-review manuscripts.

Reviewer #5 (Remarks to the Author)

I co-reviewed this manuscript with one of the reviewers who provided the listed reports. This is part of the Nature Communications initiative to facilitate training in peer review and to provide appropriate recognition for Early Career Researchers who co-review manuscripts.

References

(1) Hahntow, I. N.; Schneller, F.; Oelsner, M.; Weick, K.; Ringshausen, I.; Fend, F.; Peschel, C.; Decker, T. Cyclin-Dependent Kinase Inhibitor Roscovitine Induces Apoptosis in Chronic Lymphocytic Leukemia Cells. *Leukemia* **2004**, *18* (4), 747–755. https://doi.org/10.1038/sj.leu.2403295.

(2) Bach, S.; Knockaert, M.; Reinhardt, J.; Lozach, O.; Schmitt, S.; Baratte, B.; Koken, M.; Coburn, S. P.; Tang, L.; Jiang, T.; Liang, D.; Galons, H.; Dierick, J.-F.; Pinna, L. A.; Meggio, F.; Totzke, F.; Schächtele, C.; Lerman, A. S.; Carnero, A.; Wan, Y.; Gray, N.; Meijer, L. Roscovitine Targets, Protein Kinases and Pyridoxal Kinase*[Boxs]. *Journal of Biological Chemistry* **2005**, *280* (35), 31208–31219. https://doi.org/10.1074/jbc.M500806200.

(3) Ghosh, A.; Ahmed, A.; Amoiradaki, K.; Balazs, A. Y. S.; Barlaam, B.; Bodnarchuk, M. S.; Collie, G. W.; Dale, I. L.; Denz, C. R.; Drew, L.; Edmondson, S. D.; Fan, J.; Fawell, S.; Goldberg, F. W.; Gopalsamy, A.; Grondine, M.; Guo, G.; Hande, S. M.; Hatoum-Mokdad, H.; Hird, A. W.; Howells, R.; Hao-Ru Hsu, J.; Hudson, J.; Jackson, A.; Lamb, M. L.; Lamont, G. M.; Lamont, S.; Lichtor, P. A.; McWilliams, L.; Milne, D.; Mlynarski, S. N.; Narasimhan, P.; Peters, M. F.; Pflug, A.; Pollard, H. K.; Qin, M.; Reimer, C.; Robbins, K. J.; Robinson, J.; Sha, L.; She, H.; Sheppeck, J. E. I.; Singh, B.; Song, K.; Su, Q.; Telmesani, R.; Throner, S.; Vasalou, C.; Wang, L.; Wang, Y.; Wilson, D. M.; Winlow, P.; Yang, W.; Yao, T.; Zhang, Y.; Zhang, Z.; Zindel, D.; Johannes, J. W. Discovery of AZD8421: A Potent CDK2 Inhibitor with Selectivity Against Other CDK Family Members and the Human Kinome. *J. Med. Chem.* **2025**, *68* (17), 18164–18177. https://doi.org/10.1021/acs.jmedchem.5c01478.
